# Supplementary material for: Influence of the Type of Amino Acid on the Permeability and Properties of Ibuprofenates of Isopropyl Amino Acid Esters
Source: Int J Mol Sci. 2022 Apr 9;23(8):4158. doi: 10.3390/ijms23084158 (PMC9025941; doi:10.3390/ijms23084158)
Supplement: Supplementary file 1 [file ijms-23-04158-s001.zip › ijms-1631267 Supplementary materials- author revision.pdf]

Supplementary Materials

Influence of the type of amino acid on the permeability and properties of ibuprofenates of isopropyl amino acid esters.

by Paula Ossowicz-Rupniewska [1*], Joanna Klebeko [1], Ewelina Świątek [1], Anna Nowak [2], Wiktoria Duchnik [3], Łukasz Kucharski [2], Łukasz Struk [4], Karolina Bilska [1], Karolina Wenelska [5], Adam Klimowicz [2], and Ewa Janus [1]

[1] Department of Chemical Organic Technology and Polymeric Materials, Faculty of Chemical Technology and Engineering, West Pomeranian University of Technology in Szczecin, Piastów Ave. 42, 71-065 Szczecin, Poland

[2] Department of Cosmetic and Pharmaceutical Chemistry, Pomeranian Medical University in Szczecin, Powstańców Wielkopolskich Ave. 72, 70-111 Szczecin, Poland

[3] Department of Farmaceutical Chemistry, Pomeranian Medical University in Szczecin, Powstańców Wielkopolskich Ave. 72, 70-111 Szczecin, Poland

[4] Department of Organic and Physical Chemistry, Faculty of Chemical Technology and Engineering, West Pomeranian University of Technology, Szczecin, Piastów Ave. 42, 71-065 Szczecin, Poland

[5] Department of Nanomaterials Physicochemistry, Faculty of Chemical Technology and Engineering, West Pomeranian University of Technology in Szczecin, Piastów Ave. 45, 70-311 Szczecin, Poland

[*]possowicz@zut.edu.pl

Number of pages: 90
Number of Figures: 102
Number of Tables: 1

Table of Contents

Materials and Methods	2-9
The NMR spectra of [AAOiPr][IBU]	10-37
The ATR-FTIR spectra of [AAOiPr][IBU]	38-51
The UV-Vis spectra of [AAOiPr][IBU]	52-58
The TG curves of [AAOiPr][IBU]	59-65
The DSC curves of [AAOiPr][IBU]	66-73
X-ray diffraction (XRD) patterns of [AAOiPr][IBU]	74-87
Skin permeation results	88-90

Materials and Methods

Materials

Unless otherwise noted, all the materials and solvents were purchased from commercial suppliers and used without further purification. Glycine (purity ≥99%), L-alanine (purity ≥98.5%), L-valine (purity ≥98.5%), L-leucine (purity ≥98.5%), L-cysteine (purity ≥99%), L-serine (purity ≥98.5%), L-methionine (purity ≥99%), L-phenylalanine (purity ≥98.5%), L-proline (purity ≥98.5%) were purchased from ROTH. L-isoleucine (purity ≥98%), L-threonine (purity ≥97%), and L-aspartic acid (purity ≥98%) were purchased from Fluorochem. L-lysine (purity ≥97%), 2-(4-isobutylphenyl)propanoic acid (ibuprofen, racemic mixture, purity ≥98%) were provided from AmBeed. Ibuprofen sodium salt (purity ≥98%), chlorotrimethylsilane (purity ≥98%), PBS tablets, and DMSO-d_6 (99.9 atom% D) were purchased from Sigma-Aldrich. $CDCl_3$ (purity 99.8%; containing 0.03% TMS) was purchased from Eurisotop. MeOH (performance liquid chromatography) was purchased from Alfa Aesar. Acetonitrile (performance liquid chromatography), n-hexane (purity 99%), ethanol (purity 99.8%), diethyl ether (purity 99.5%), toluene (ACS) were purchased from Avantor. Propan-2-ol (ACS), chloroform (ACS), dichloromethane (ACS), ethyl acetate (ACS), and ammonia solution (25%) were provided from Stanlab. DMSO (ACS), Na_2SO_4 (ACS), and NaCl (ACS) were purchased from Chempur. Porcine skin was provided from a local slaughterhouse.

Methods

Nuclear magnetic resonance spectroscopy (NMR)

^{1}H NMR and ^{13}C NMR were recorded on a BRUKER DPX-400 spectrometer (400 MHz and 100 MHz). Signal positions were recorded in ppm with the abbreviations s, d, dd, ddd, t, q, hept, and m, denoting singlet, doublet, doublet of doublets, doublet of doublet of doublets, triplet, quartet, heptuplet, and multiplet, respectively. All NMR chemical shifts were reported with the solvent resonance as the internal standard. For ^{1}H NMR: $CDCl_3$ = δ 7.26 ppm, DMSO-d_6= δ 2.50 ppm. For ^{13}C NMR: $CDCl_3$ = δ 77.1 ppm, DMSO-d_6 = δ 39.8 ppm

Total reflectance – Fourier transform infrared spectroscopy (ATR-FTIR)

ATR-FTIR spectra data were recorded on Thermo Scientific Nicolet 380 spectrometer equipped with an ATR diamond plate. The spectra were recorded in transmission mode in the range of 4000 – 400 cm^{-1} at 4 cm^{-1}.

UV-Vis spectroscopy (UV-Vis)

UV-Vis spectra were collected on a Spectroquant® Pharo 300 Spectrophotometer from Merck (Darmstadt, Germany). The solutions were prepared in absolute ethanol of concentration range 10^{-4} – 10^{-5} M. The measurements were performed in a 10 mm quartz cell in the wavelength range of 190 – 400 nm with the accuracy of ± 1 nm.

Elemental analysis

The content of elements, i.e. nitrogen, carbon, hydrogen, and oxygen, was determined by CHNS/O elemental analysis. The elemental analysis was performed using an elemental analyzer (Thermo Scientific™ FLASH 2000 CHNS/O Analyzer).

The Elemental Analyser operates according to the dynamic flash combustion of the sample. For CHNS determination, samples are weighed in tin containers. After the combustion, the resulting gases are carried by a helium flow to a layer filled with copper, then swept through a GC column that provides the separation of the combustion gases. Finally, they are detected by a Thermal Conductivity Detector (TCD). For oxygen determination, the system operates in pyrolysis mode. Samples are weighed in silver containers and introduced via the MAS Plus Autosampler into the pyrolysis chamber. The reactor contains nickel-coated carbon maintained at 1060 ˚C. The oxygen in the sample, combined with the carbon, forms carbon monoxide, then gas chromatographically separated from other products and detected by the TCD Detector.

Thermogravimetric analysis (TG)

The thermogravimetric (TG) analysis was determined on a Netzsch Proteus Thermal Analysis TG 209 F1 Libra apparatus (Selb, Germany). Measurements were performed under an oxidizing atmosphere, the nitrogen flow was 10 $cm^3 \cdot min^{-1}$, and the airflow was 25 $cm^3 \cdot min^{-1}$. The temperatures ranges were 25 – 1000 °C. The tests were carried out using alumina crucibles (Al_2O_3), and the sample weight was in the field of about 5 mg.

Differential scanning calorimetry (DSC)

DSC was performed using TA Instruments, model Q-100 DSC. The sample was loaded on an aluminum pan with a pierced lid. The analysis was carried out in a nitrogen atmosphere. The sample was heated from 20 °C to a specified temperature (individual for each compound temperature, determined from TG analysis, and it was at least 10 °C lower than the onset decomposition temperature. Indium and mercury were used as standards to calibrate the temperature. Heat calibration used indium.

Specific rotation

The specific rotation was tested using the Autopol IV automatic polarimeter from Rudolph Research Analytical at the temperature of 20 °C and in 589 nm the wavelength. The specific rotation was determined using equation 1, and the molar one using equation 2:

$$[\alpha]_{\lambda}^{T} = \frac{\alpha_{\lambda}^{T}}{c \cdot l} \quad (1)$$

$$[M]_{\lambda}^{T} = \frac{M \cdot [\alpha]_{\lambda}^{T}}{100} \quad (2)$$

where:

α_{λ}^{T} - optical rotation at temperature T and wavelength λ [°Arc]

$[\alpha]_{\lambda}^{T}$ - specific rotation at temperature T and wavelength λ

c – concentration [g/cm^3]

l – wavelength [dm].

M – the molar mass of the tested substance [g/mol].

Partition coefficient

The n-octanol – water partition coefficient was investigated by the shake flask method. For this purpose, 10 mg of the substance, weighed with an accuracy of 0.01 mg, was added to 5 cm^3 of water (or buffer) saturated with n-octanol and 5 cm^3 of n-octanol saturated with water. The mixture was vigorously agitated at 25 °C for 3 hours, followed by centrifugation at 7500 rpm, at 25 °C for 10 min for better phase separation. Then aqueous centrifugation layer was decanted and analysed by HPLC to determine the concentration of the compound.

The concentration of the substance in the aqueous layer was determined by high-performance liquid chromatography SHIMADZU Nexera-i LC-2040C 3D High Plus liquid chromatograph with a DAD/FLD detector. As a mobile phase, a mixture of 50% acetonitrile and 50% water was used

under isocratic conditions, with a flow rate of 1 $cm^3 \cdot min^{-1}$. A Kinetex® 2.6 µm F5 100 Å column with dimensions 150 × 4.6 mm^2 from Phenomenex and heated to 30 °C was used. The detection wavelength was 210 nm. Data acquisition and processing were performed using a LabSolutions/LC Solution System (software). The injection volume for these samples was 50 µL. Each measurement was performed in triplicate, and the results were averaged. The concentration of ibuprofen and its salts were calculated on peak area measurements using a calibration curve (Figure S1) method.

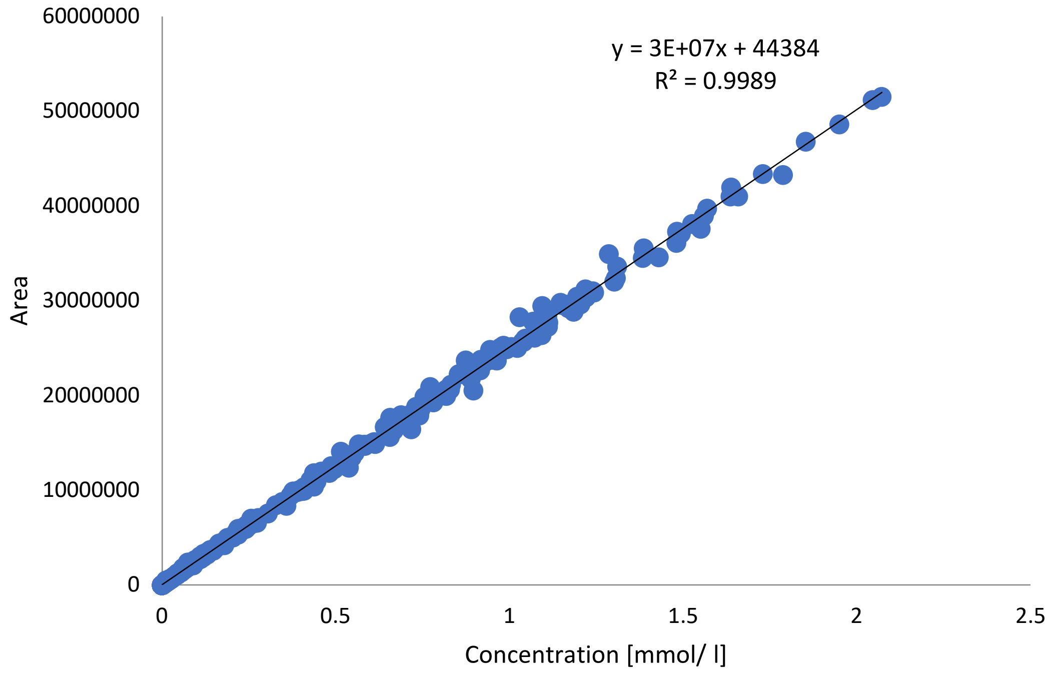

Figure S1. Calibration curve plot for amino acid isopropyl ester ibuprofenates.

The concentration of the compound dissolved in octanol was calculated with equation 3, and the partition coefficient log P was calculated by equation 4:

$$c_o = c_0 - c_w \qquad (3)$$

$$\log P = log c_o - log c_w \qquad (4)$$

where:

c_w - concentration of the substance dissolved in an aqueous layer [$mg \cdot dm^{-3}$]

c_o - concentration of the substance dissolved in the octanol layer [$mg \cdot dm^{-3}$]

c_0 - total concentration, calculated based on the mass of the compound used in the experiment [$mg \cdot dm^{-3}$]

Solubility

Solubility was evaluated in polar and nonpolar solvents by modified Vogel's method [42] at the temperature of 25 °C. This method classified the compound as soluble, partly soluble, and practically insoluble. Dimethyl sulfoxide, ethanol, chloroform, ethyl acetate, diethyl ether, toluene, and n-hexane were used as solvents.

Solubility (saturation concentration), defined as a maximum quantity of a substance that may be dissolved at a given temperature in the unit of solvent volume, was determined in deionised water and PBS (7.4). An excess substance was added to 2 cm^3 of water or PBS in the screwed vial and was stirred vigorously at 25.00 ± 0.05 °C (for water solutions) or 32.00 ± 0.05 °C (for PBS solutions), for 24 hours. Then the mixture was centrifuged at respective temperatures, and the liquid above the solid was taken, diluted, and analysed by the HPLC method to determine the concentration of the substance. The HPLC conditions are detailed in the partition coefficient part.

XRD

X-ray diffraction (XRD) established the crystallographic information regarding obtained ibuprofenates was confirmed by X-ray diffraction (XRD), performed using an AERIS PANalytical X-ray diffractometer with Cu-Kα radiation.

SEM

Morphologies were studied using scanning electron microscopy on Tescan Vega 3, operating at 30 kV.

Statistical Analysis

Results are presented as the mean ± standard deviation (SD). A one-way analysis of variance (ANOVA) was used. In the case of the cumulative mass after 24 h permeation and the cumulative mass in the skin, the significance of differences between individual groups was evaluated with Newman-Keuls's test ($\alpha < 0.05$). The significant differences in the cumulative permeation mass, taking into account all time points throughout the 24 h permeation, were estimated by the Mann–Whitney test, where each derivative compared to the control. A cluster analysis was carried out to determine similarities between all compounds tested, taking into account all time points. On this

basis, presented groups of compounds with a similar permeation. Statistical calculations were done using *Statistica 13* PL software (StatSoft, Kraków, Polska).

Method Validation

Linearity

Linearity was determined on solutions of ibuprofen and its salts working standards within the $2.3 \cdot 10^{-3}$ – 2.0 mmol/l range. The solutions were made by dissolving ibuprofen or its salt in acetonitrile-water (50:50 v/v) and analysed in triplicate. The data obtained from the linearity experiments were subjected to linear regression analysis, with the concentration of injected standard being plotted against the peak areas obtained.

The linear regression equation was $y = 3 \cdot 10^7 x + 38765$ with a coefficient of determination (R^2) of 0.9989, where y = peak area and x = concentration of the solution.

Sensitivity

Limit of detection (LOD) and limit of quantitation (LOQ) were calculated according to equations 5 and 6, respectively.

$$LOD = \frac{3\sigma}{S} \qquad (5)$$

$$LOQ = \frac{10\sigma}{S} \qquad (5)$$

where:

σ - the standard deviation of the regression line,

S - the slope of the calibration curve.

LOD was $1.24 \cdot 10^{-5}$ mmol/l, while LOQ was $4.12 \cdot 10^{-6}$ mmol/l. Thus, the LOD and LOQ results demonstrated that the developed method was adequately sensitive for determining ibuprofen and its organic salts.

Accuracy

Accuracy was evaluated by analyzing solutions with known quantities of ibuprofen. Triplicate determinations at three concentration levels were used. The mean recoveries of the assays were assessed for compliance with the International Conference on Harmonization (ICH) guidelines.

The results for the recovery of naproxen indicate that the developed method is accurate with mean recovery in line with the ICH guidelines for the validation of analytical procedures with a mean recovery of 100% ± 1.5%

Precision

According to ICH guidelines, the parameters of repeatability and intermediate precision were evaluated as indicators of precision. Repeatability was determined by a six times injection of ibuprofen working solution, while intermediate precision was evaluated by injecting five replicate solutions of ibuprofen working standard over three consecutive days. A coefficient of variation (CV) of <1.1% of the replicate injections was used as an acceptance criterion for method precision. In all the determinations, the CV was <1.5%, and thus the proposed method was found to be precise.

Synthesis of amino acid isopropyl esters hydrochlorides [AAOiPr][HCl]

About 5 g of amino acid was dispersed into 50 mL of alkyl alcohol at room temperature. Then, two molar equivalents of TMSCl were added to the mixture. The solution was stirred thoroughly at 60ºC for complete conversion as manifested by the dissolution of substrates. Then the excess of TMSCl and alcohol and formed by-products (TMSOH or TMSR) were removed by evaporation at 60°C under a vacuum. The product was purified from the residue by washing with diethyl ether. The obtained hydrochloride was dried in a vacuum dryer at 60°C, 5 mbar for 24 h. As a result, the L-valine alkyl ester hydrochloride (ValOR·HCl) was obtained with a good yield (94-99%).

Synthesis of amino acid isopropyl esters [AAOiPr]

Obtained in the first step [AAOiPr][HCl] was added to a small amount of distilled water and neutralised by adding one to three molar equivalents of 25% ammonium hydroxide aqueous solution. The solution was intensively mixed, and then the product was extracted with diethyl ether. The organic layer was dried using anhydrous Na_2SO_4 and then concentrated under a vacuum to receive [AAOiPr].

The NMR spectra of [AAOiPr][IBU]

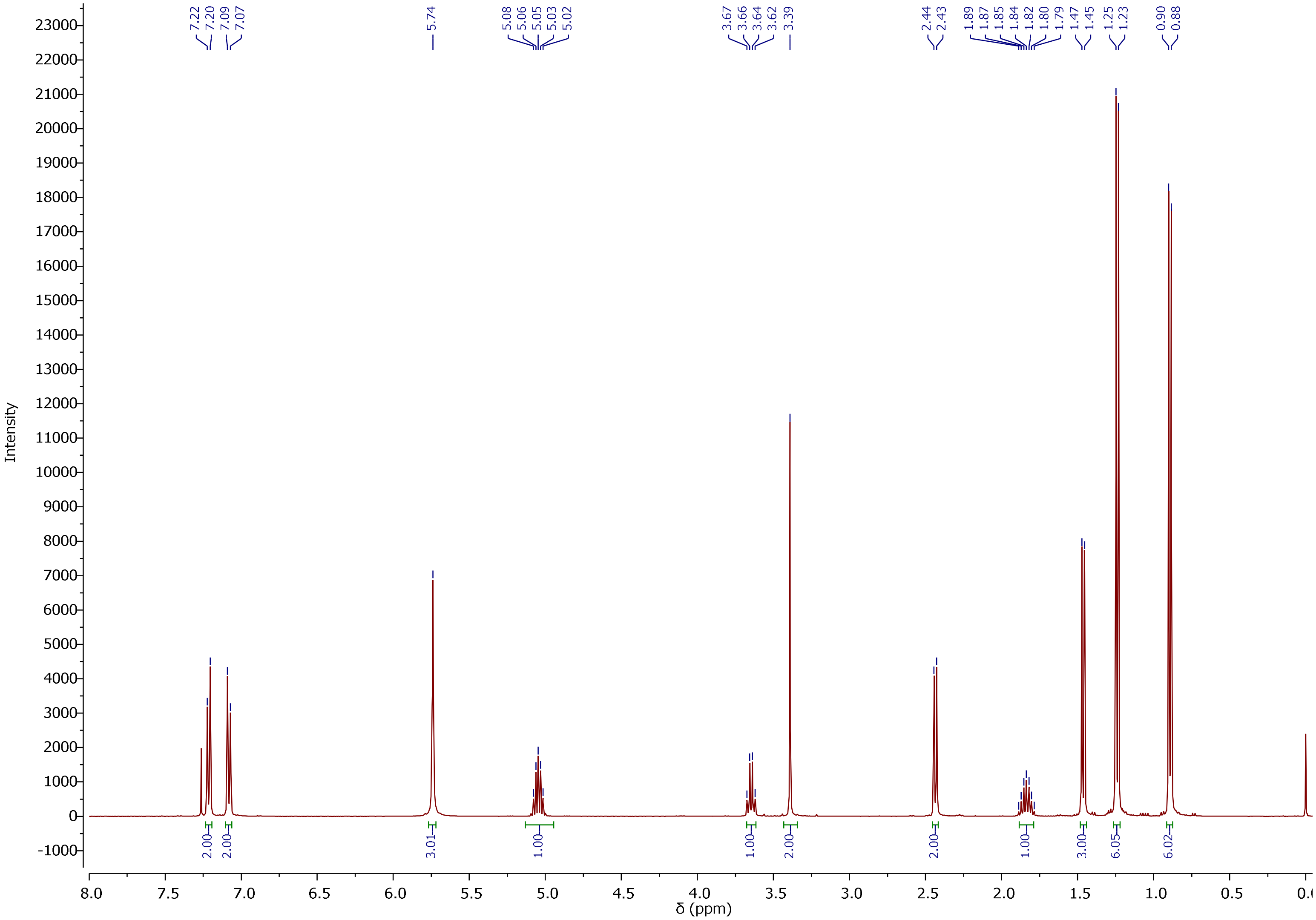

Figure S2. ^1H NMR spectra of [GlyOiPr][IBU].

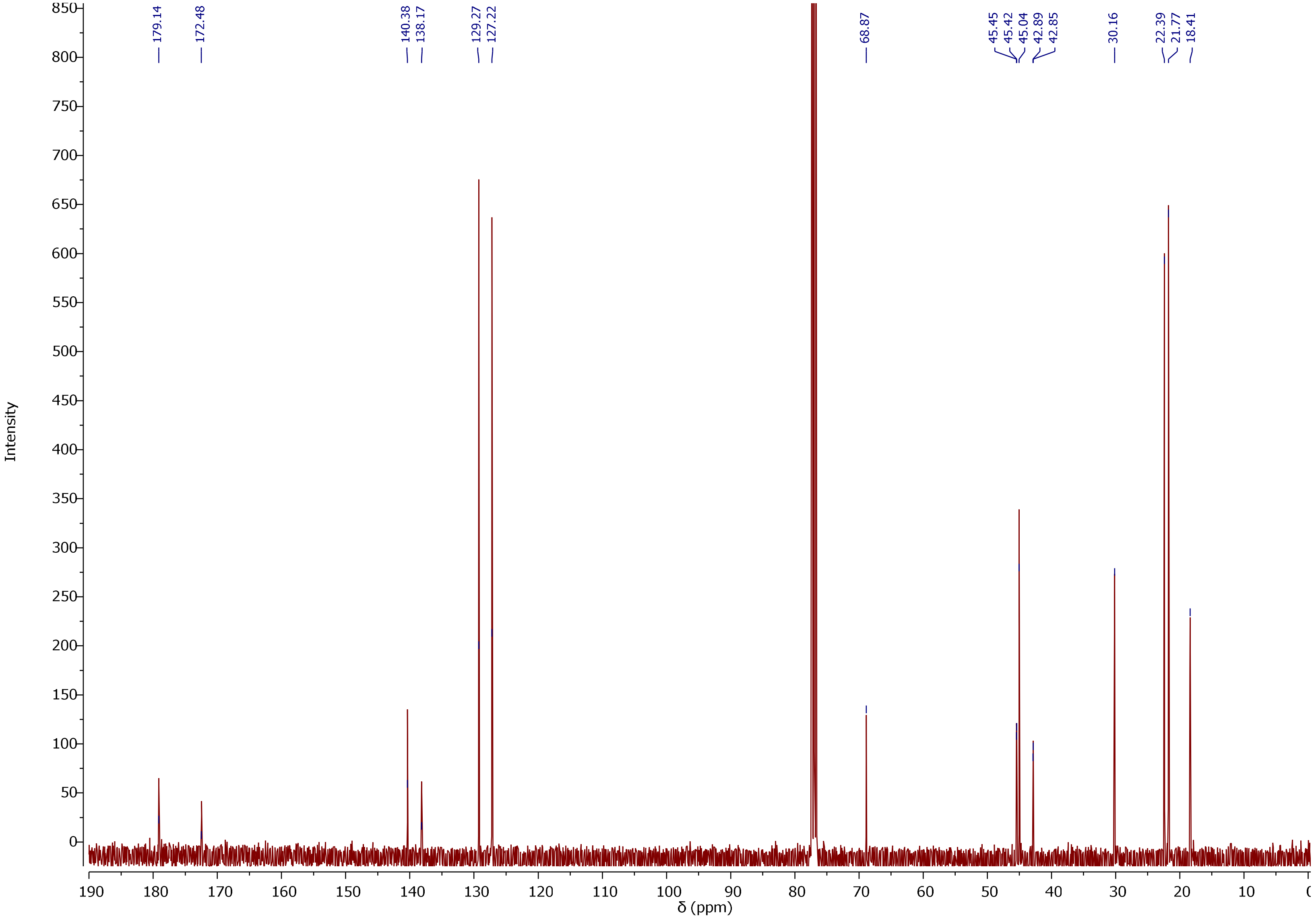

Figure S3. ^{13}C NMR spectra of [GlyOiPr][IBU].

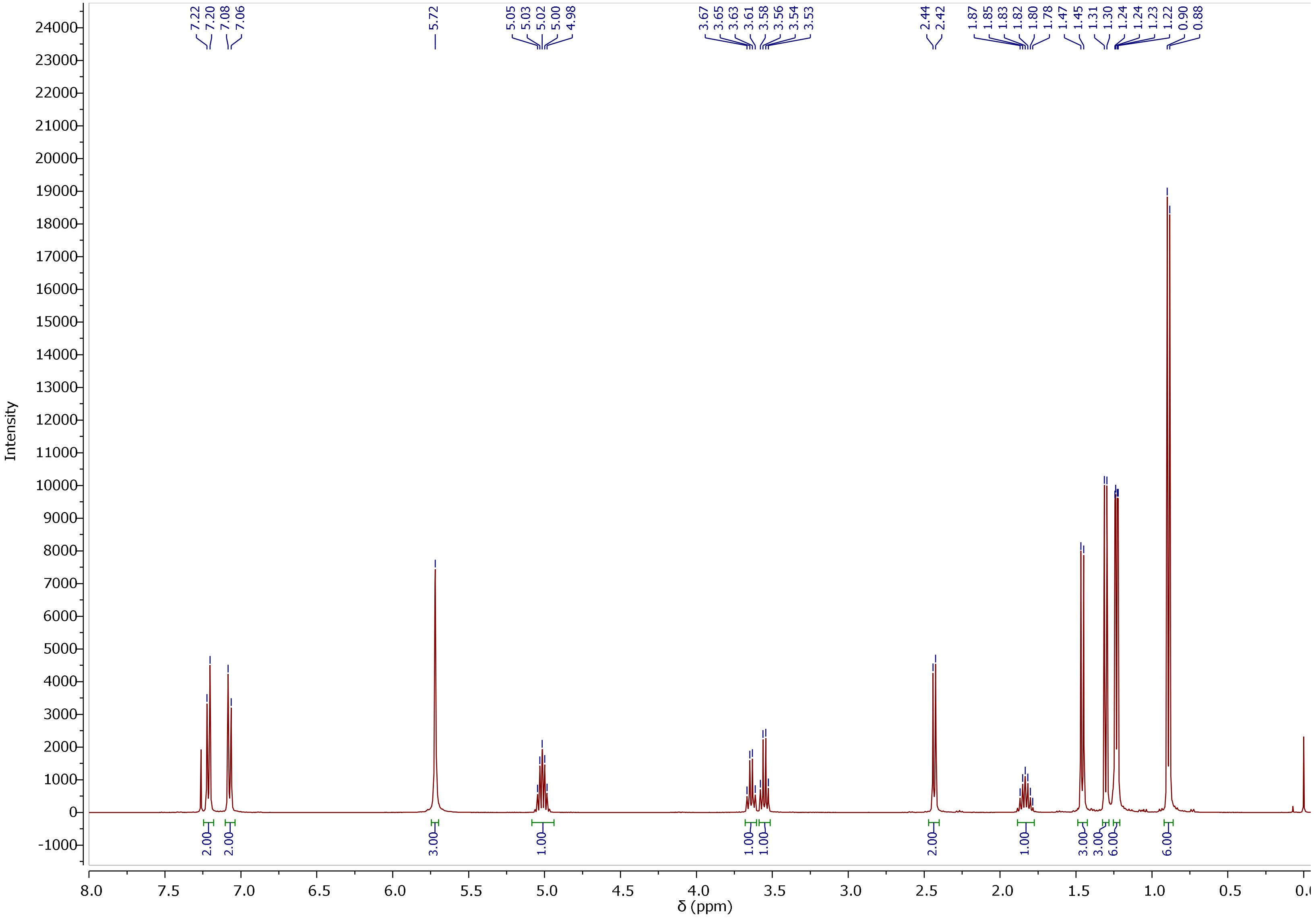

Figure S4. ¹H NMR spectra of [L-AlaOiPr][IBU].

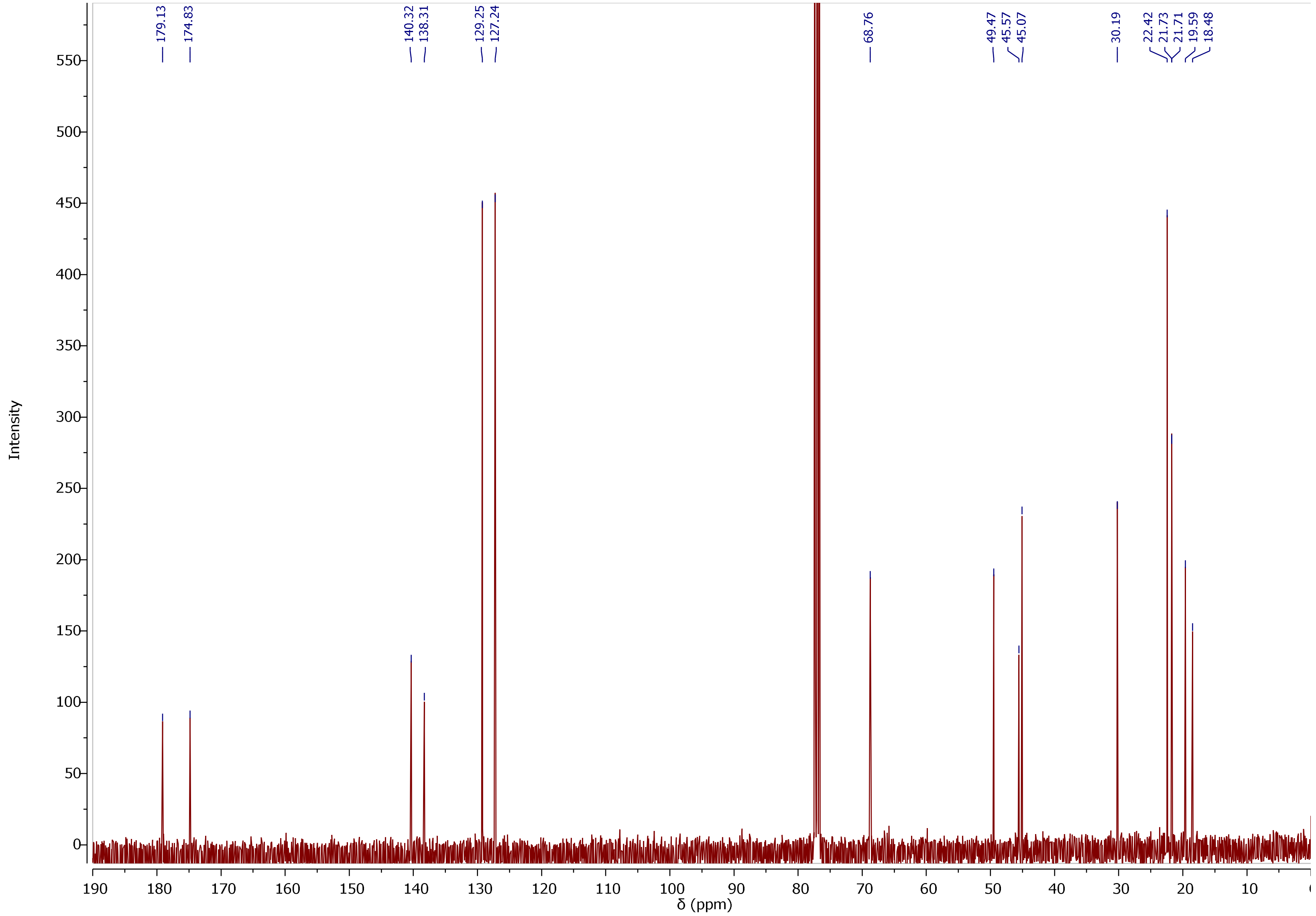

Figure S5. ^{13}C NMR spectra of [L-AlaOiPr][IBU].

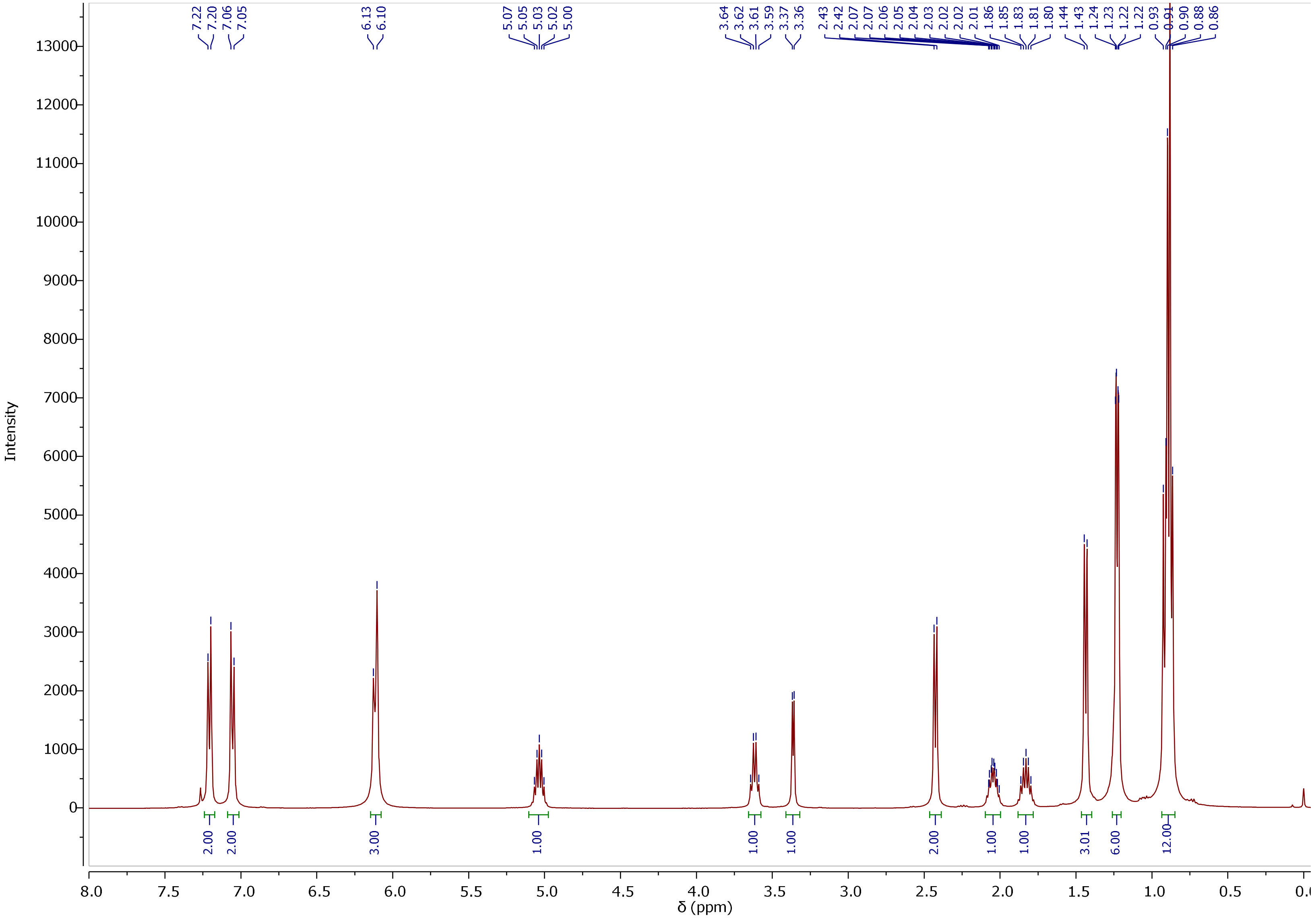

Figure S6. ^{1}H NMR spectra of [L-ValOiPr][IBU].

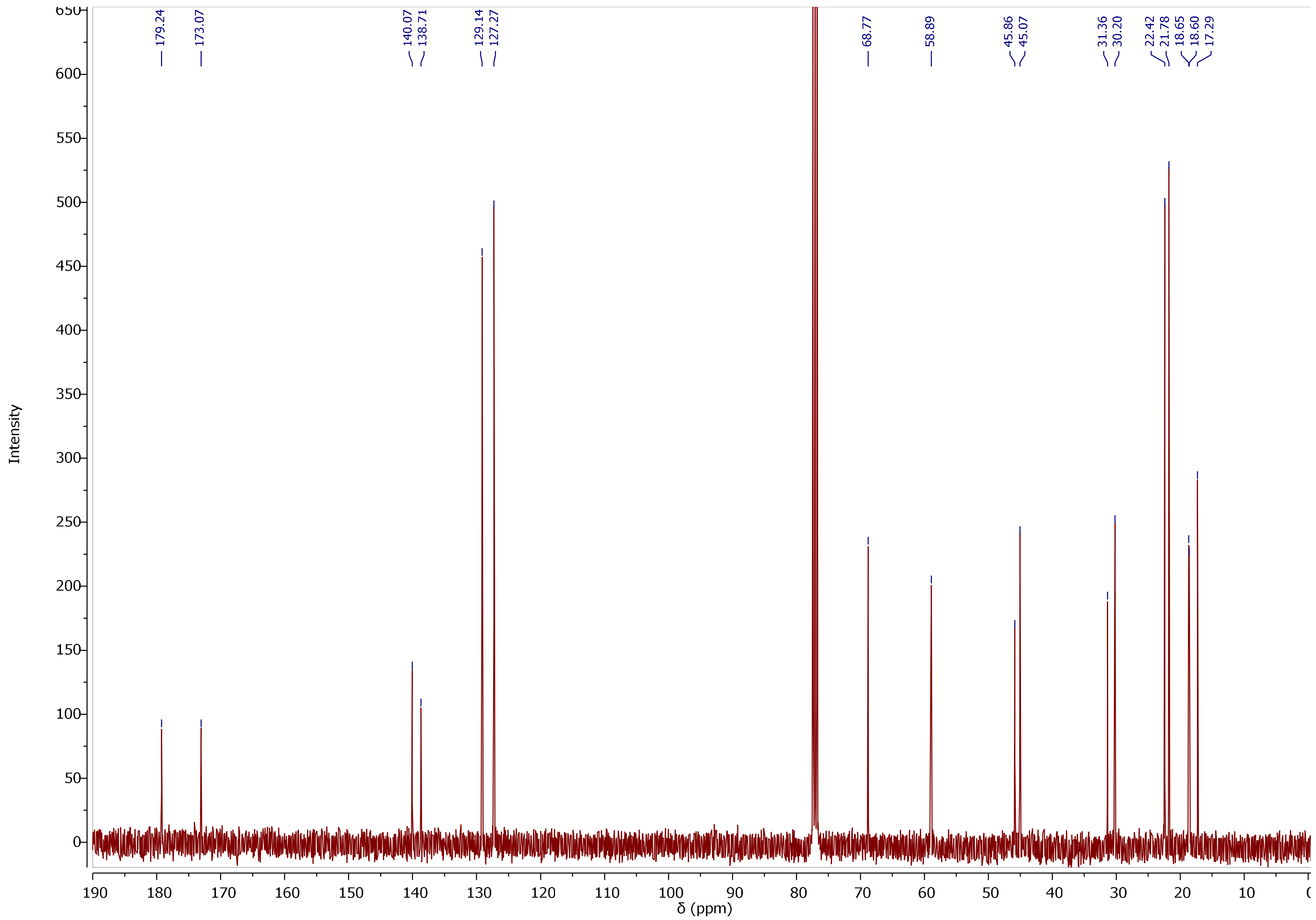

Figure S7. ^{13}C NMR spectra of [L-ValOiPr][IBU].

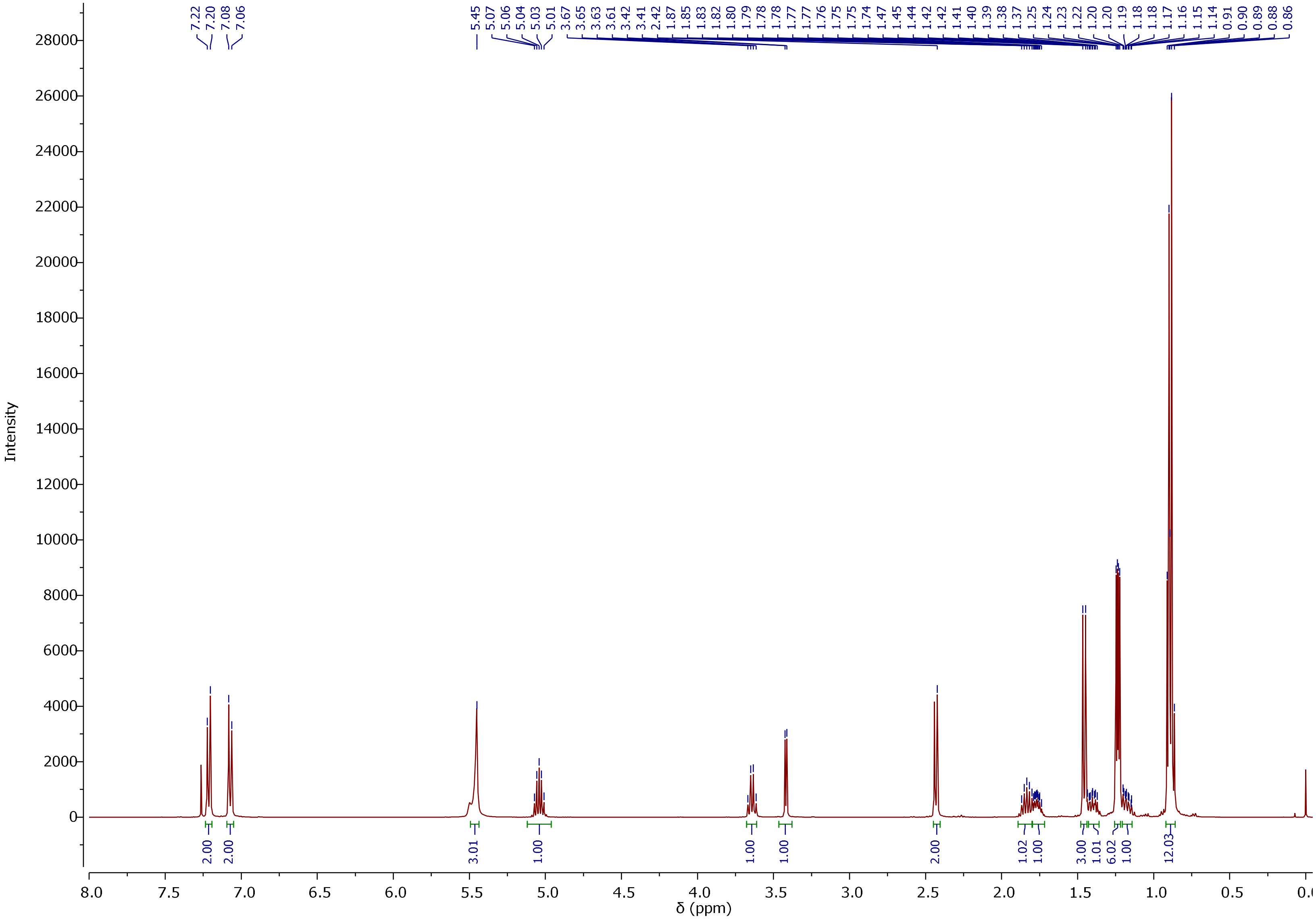

Figure S8. ^{1}H NMR spectra of [L-IleOiPr][IBU].

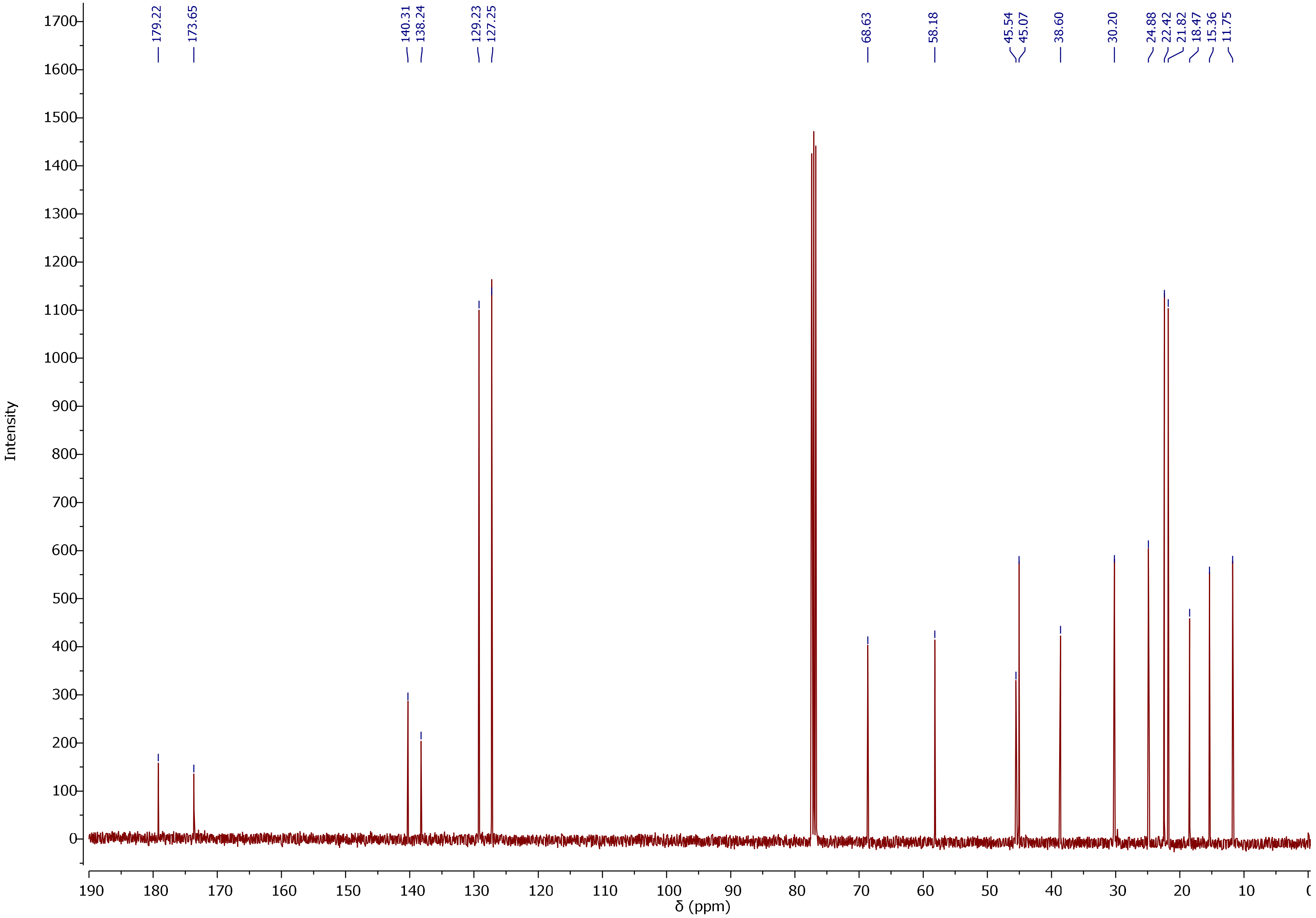

Figure S9. ^{13}C NMR spectra of [L-IleOiPr][IBU].

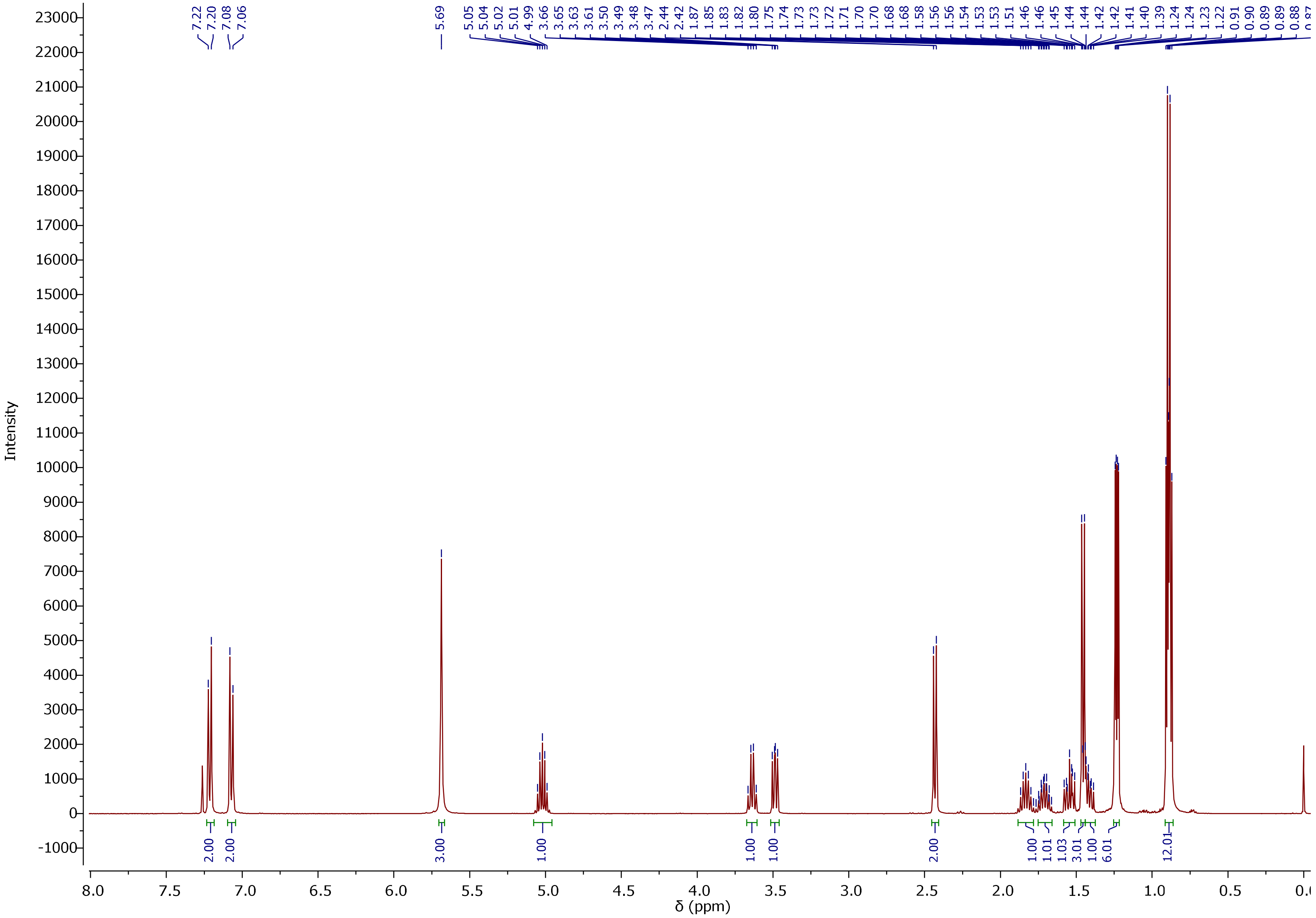

Figure S10. ^{1}H NMR spectra of [L-LeuOiPr][IBU].

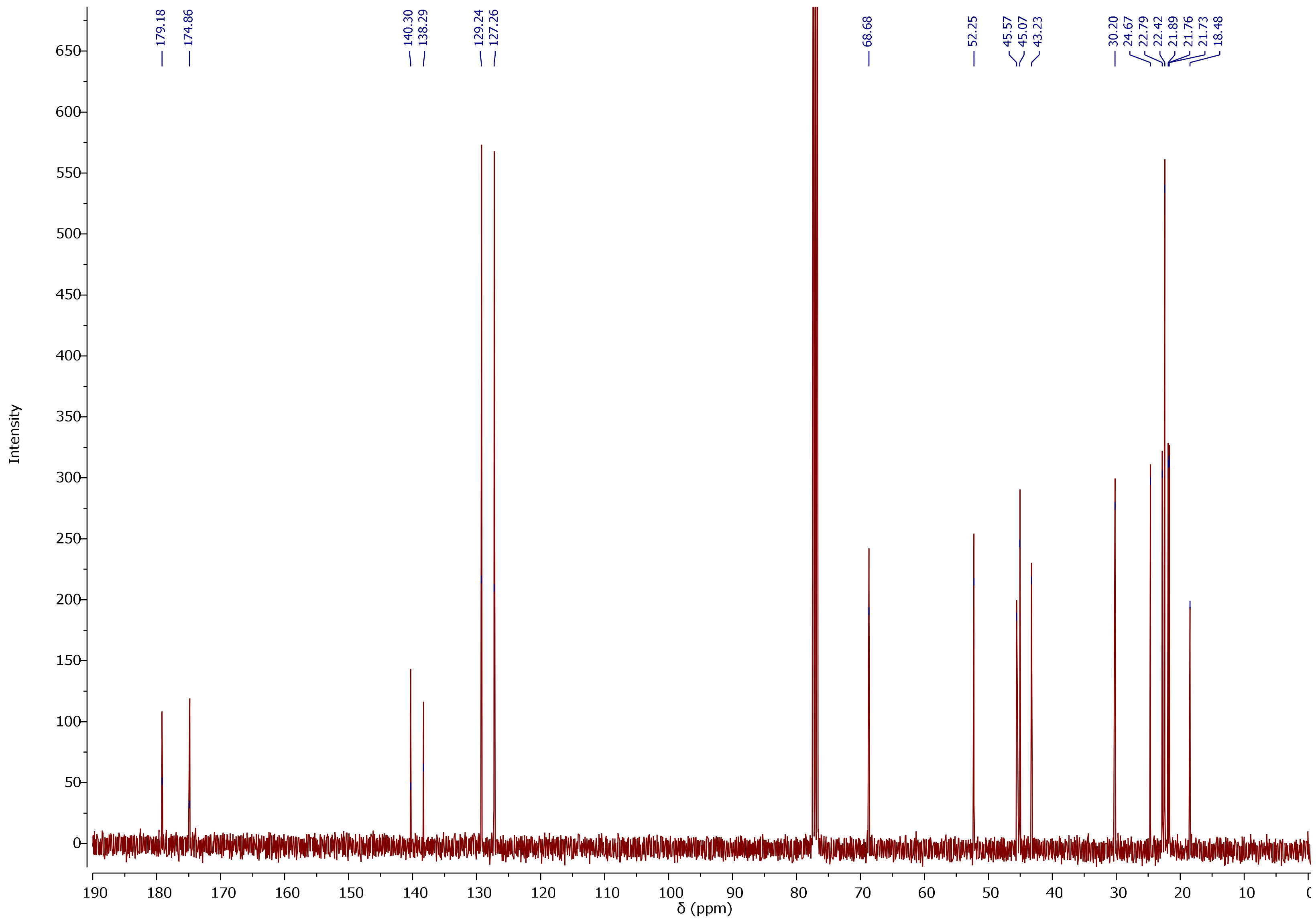

Figure S11. ^{13}C NMR spectra of [L-LeuOiPr][IBU].

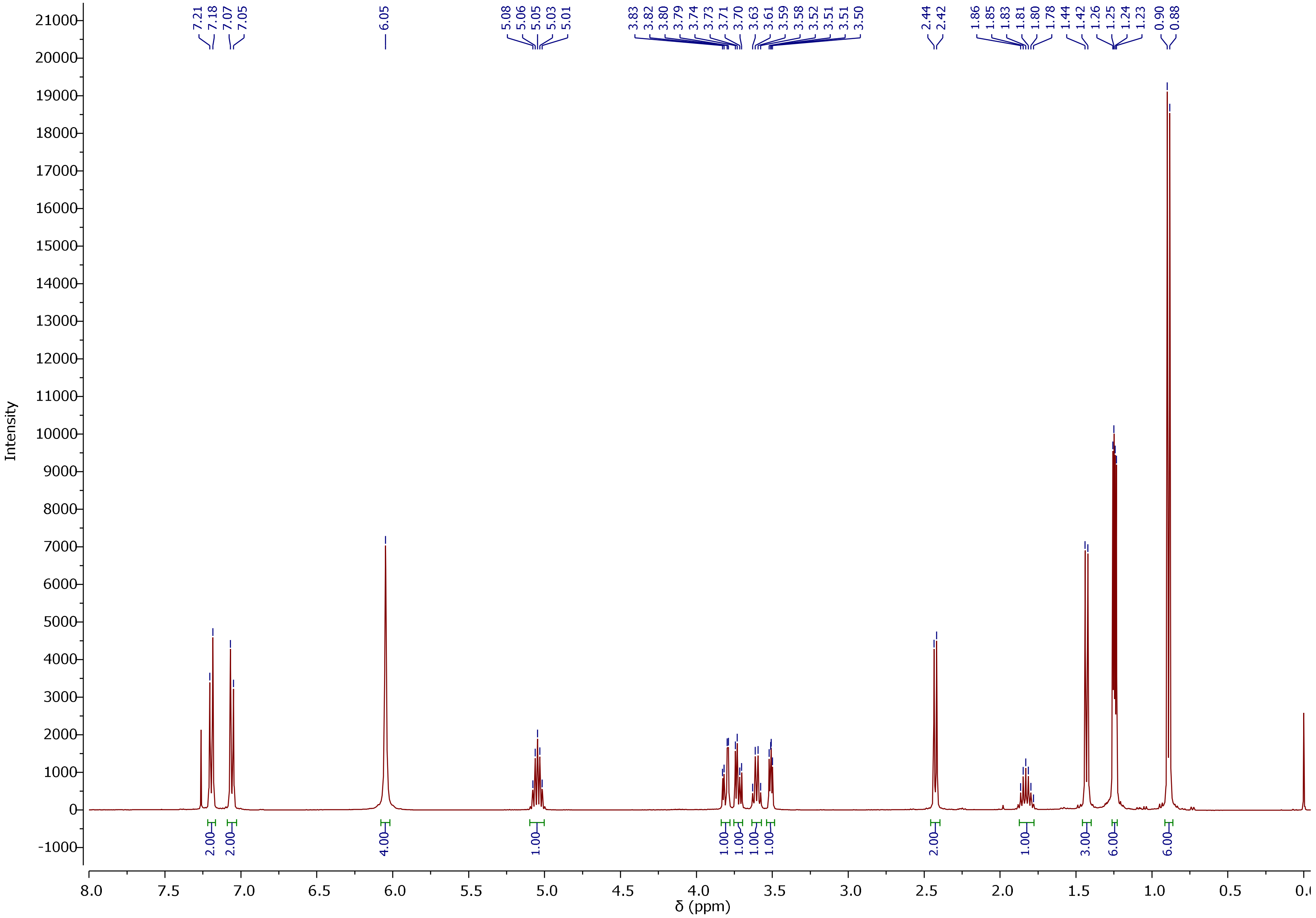

Figure S12. ^{1}H NMR spectra of [L-SerOiPr][IBU].

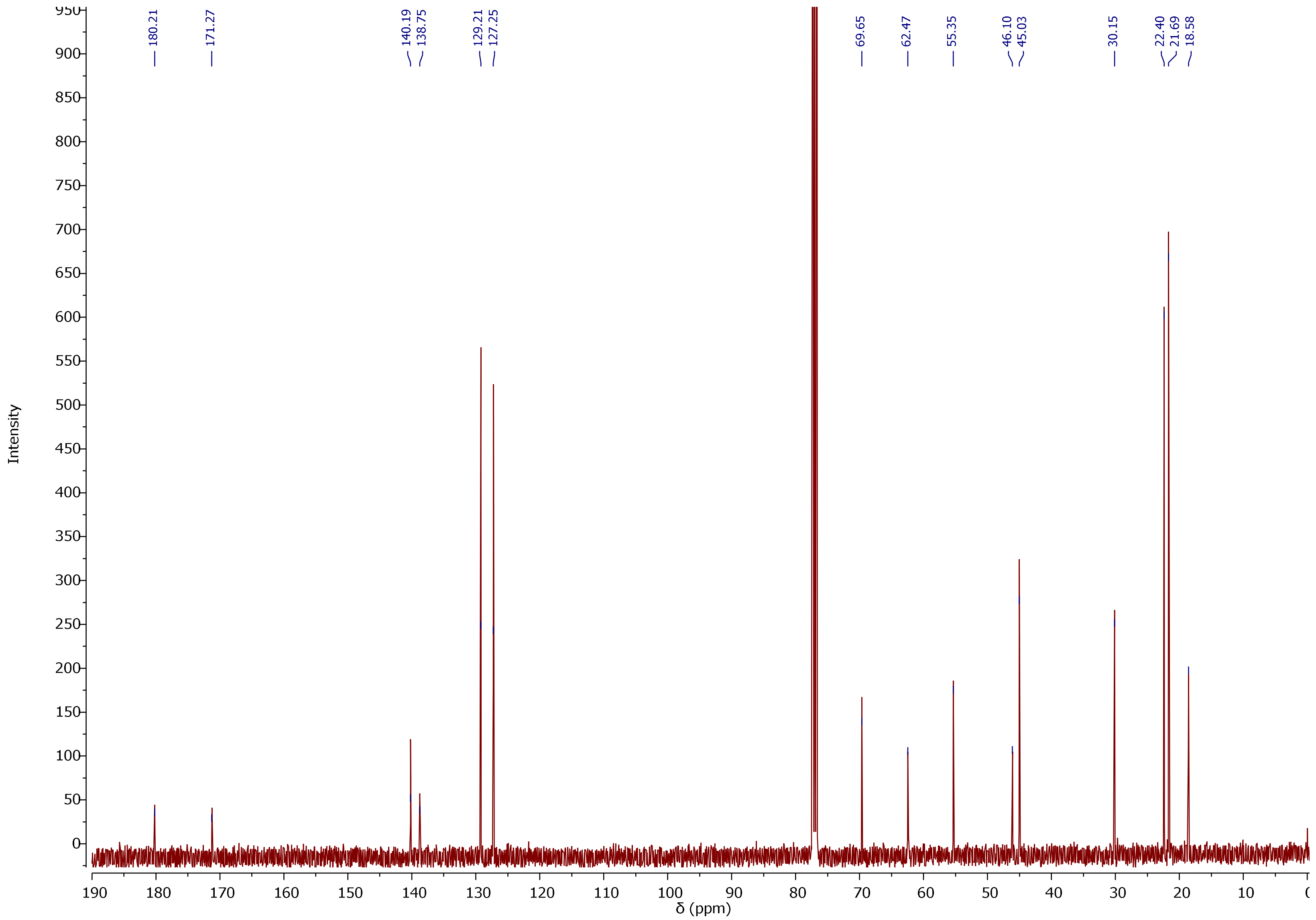

Figure S13. ^{13}C NMR spectra of [L-SerOiPr][IBU].

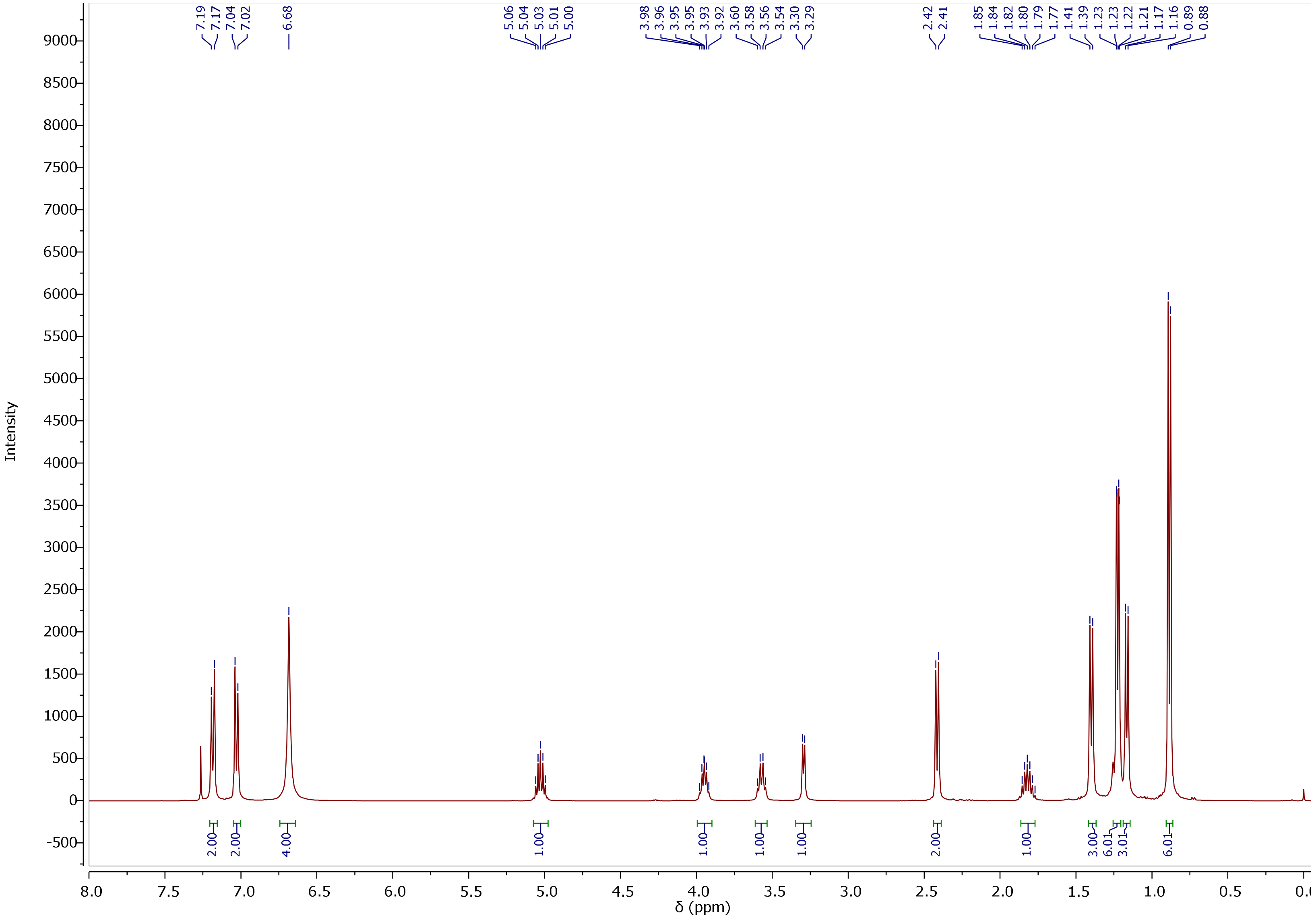

Figure S14. ^{1}H NMR spectra of [L-ThrOiPr][IBU].

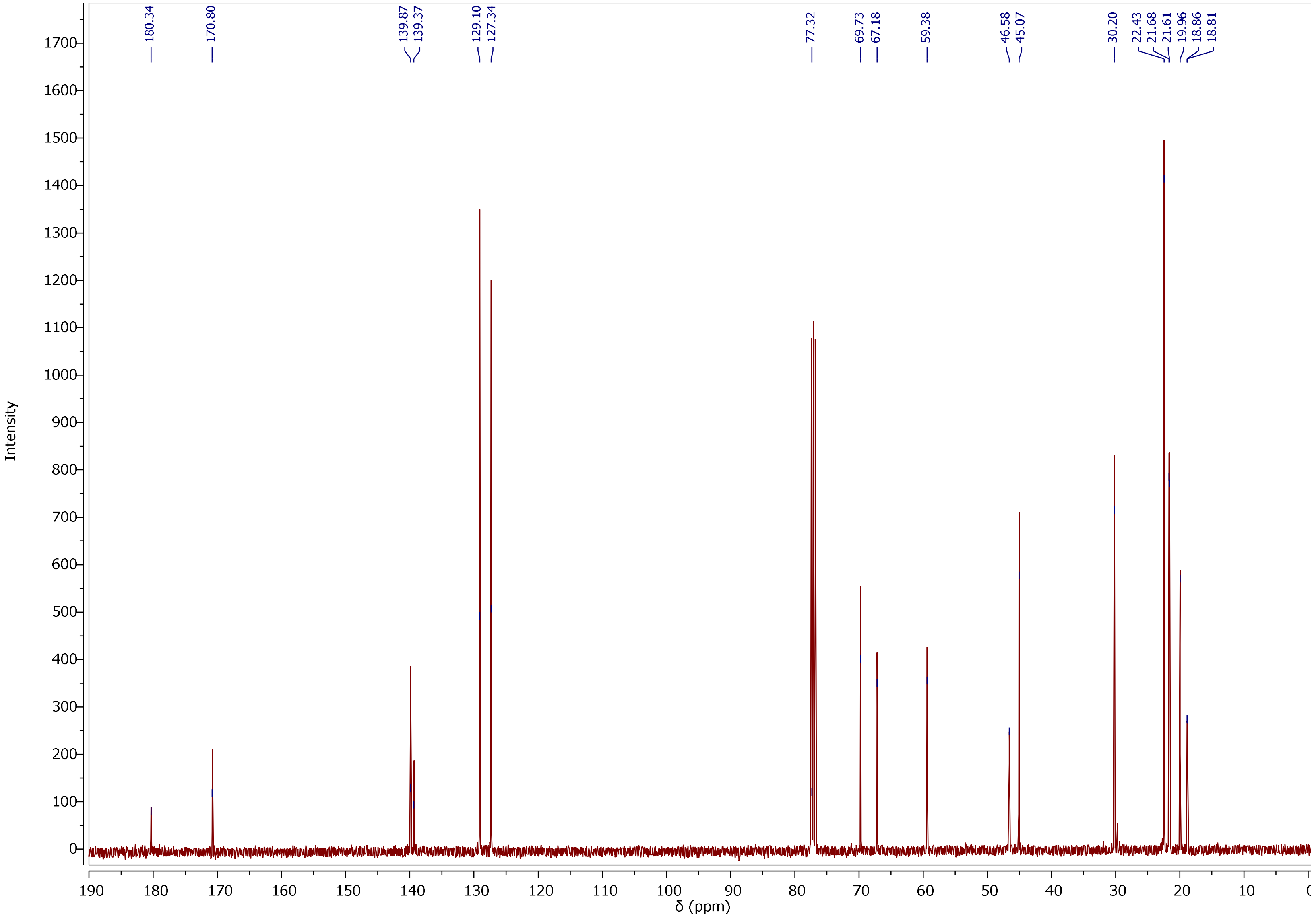

Figure S15. ^{13}C NMR spectra of [L-ThrOiPr][IBU].

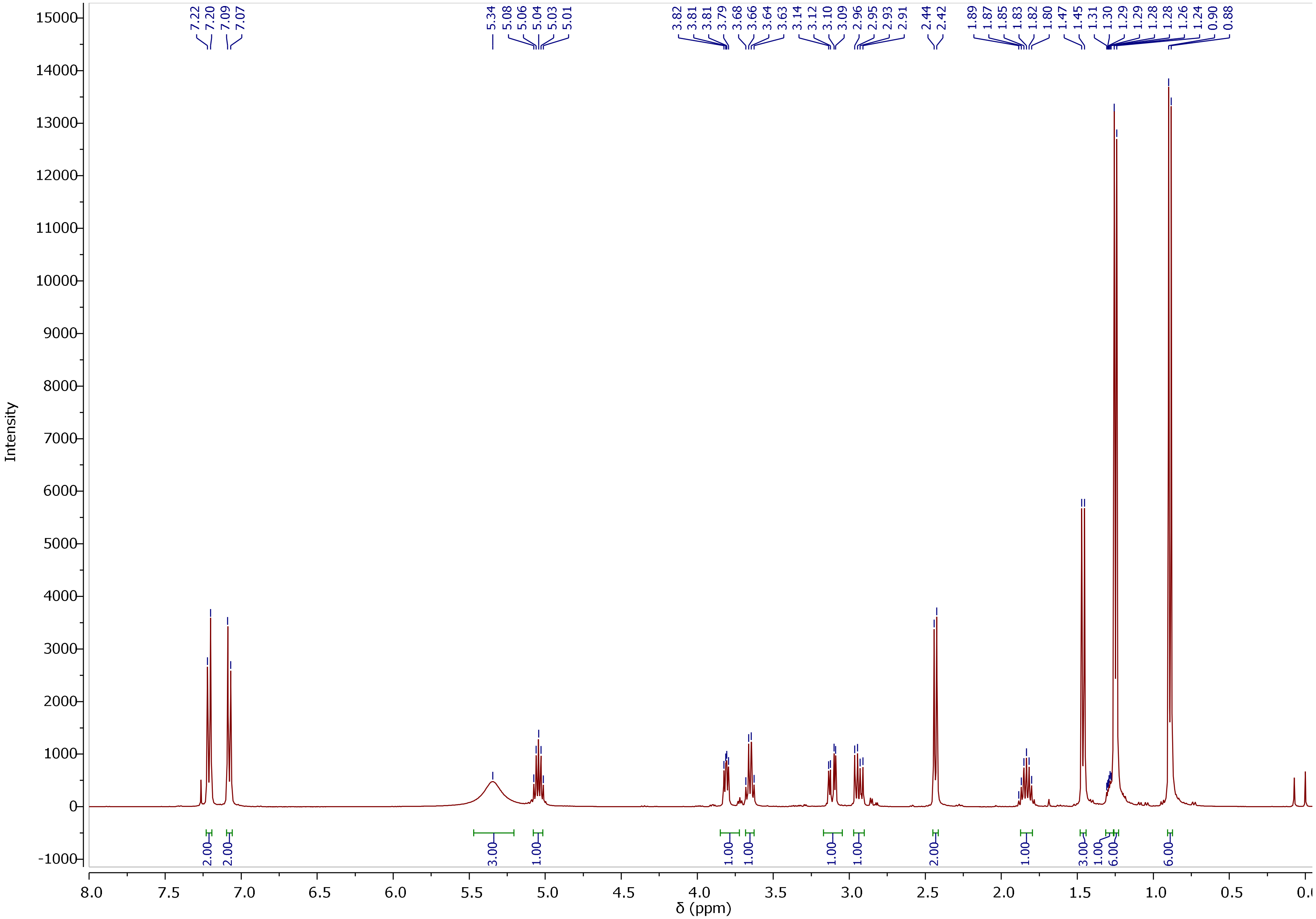

Figure S16. ¹H NMR spectra of [L-CysOiPr][IBU].

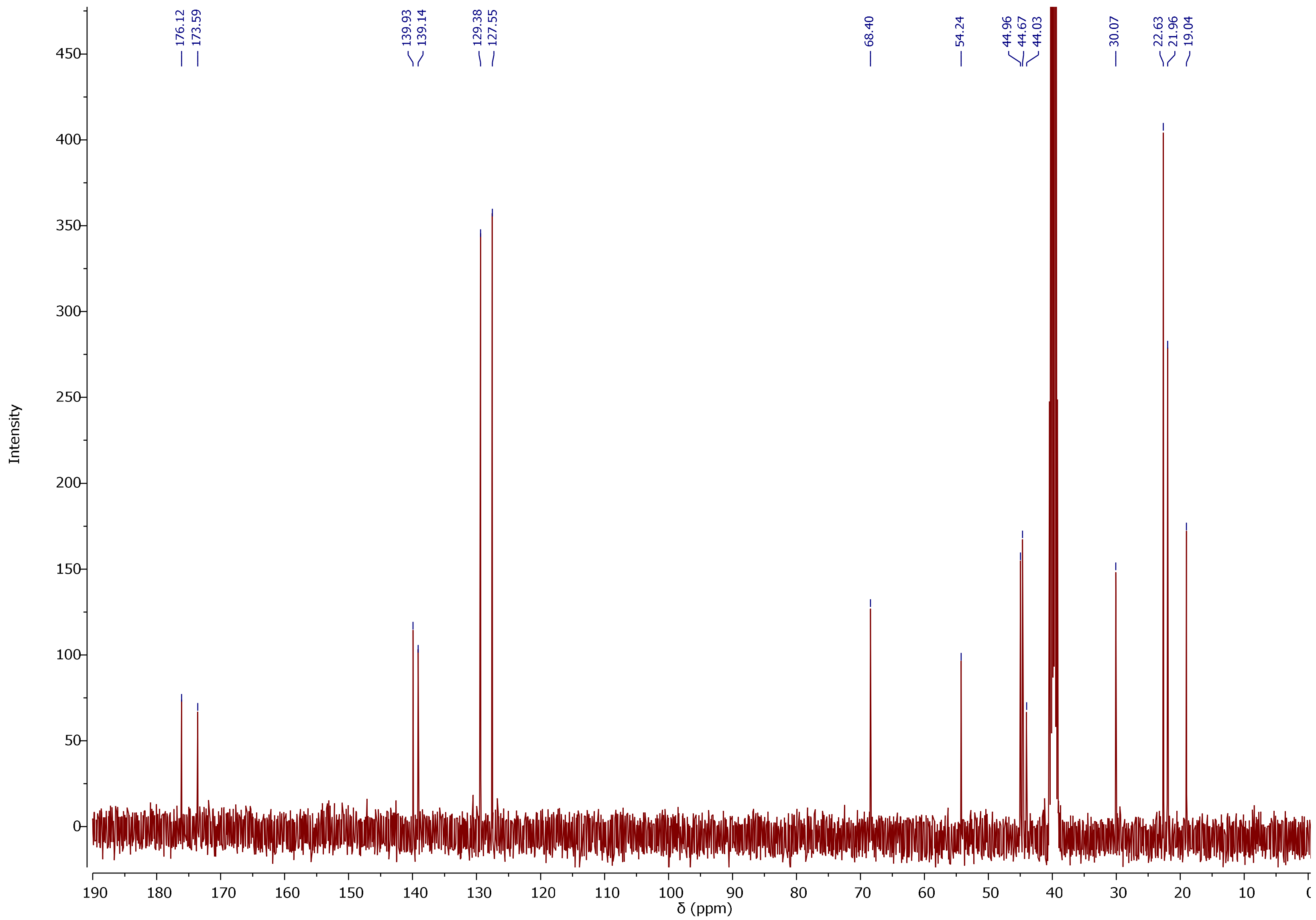

Figure S17. ^{13}C NMR spectra of [L-CysOiPr][IBU].

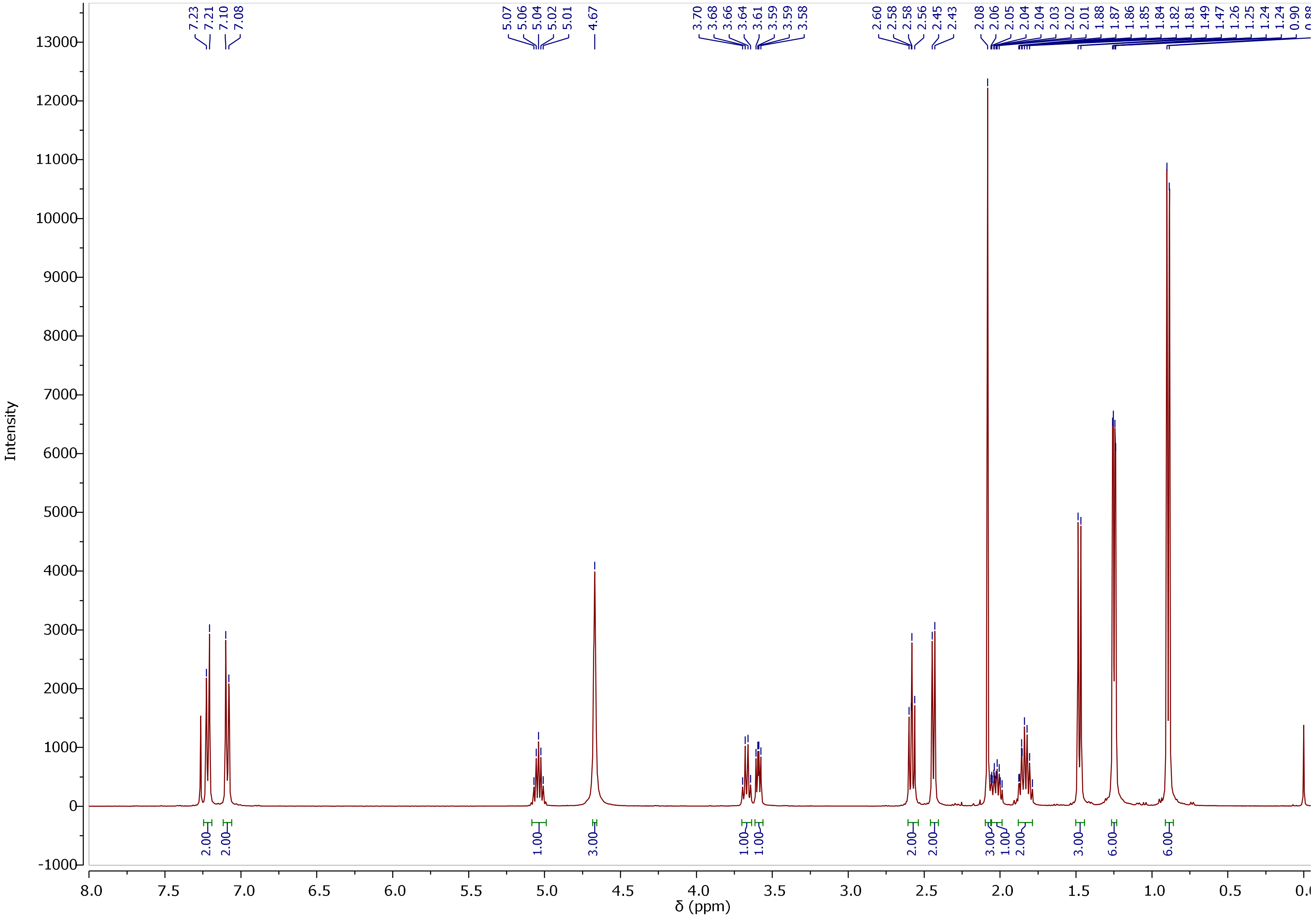

Figure S18. ^{1}H NMR spectra of [L-MetOiPr][IBU].

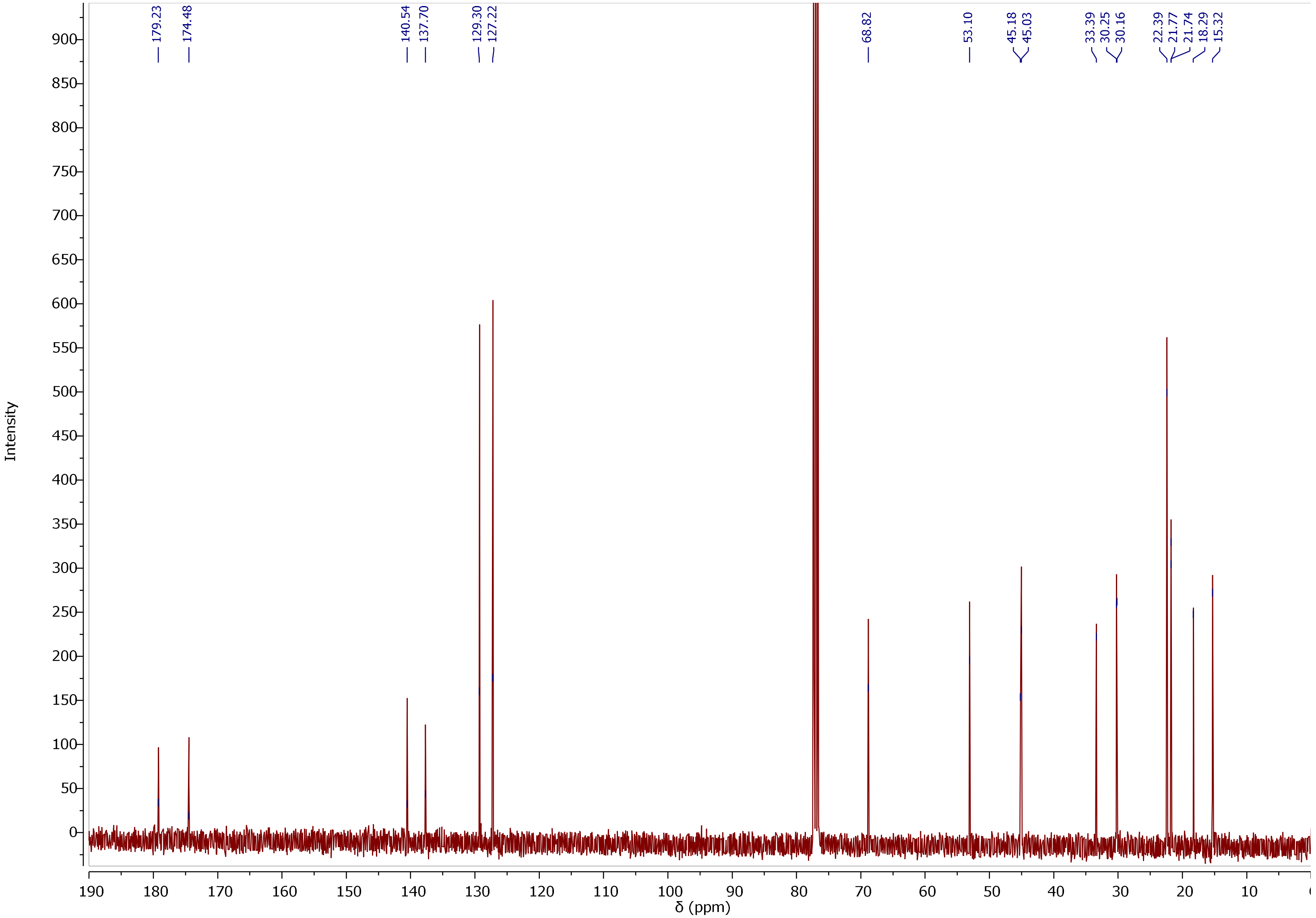

Figure S19. ^{13}C NMR spectra of [L-MetOiPr][IBU].

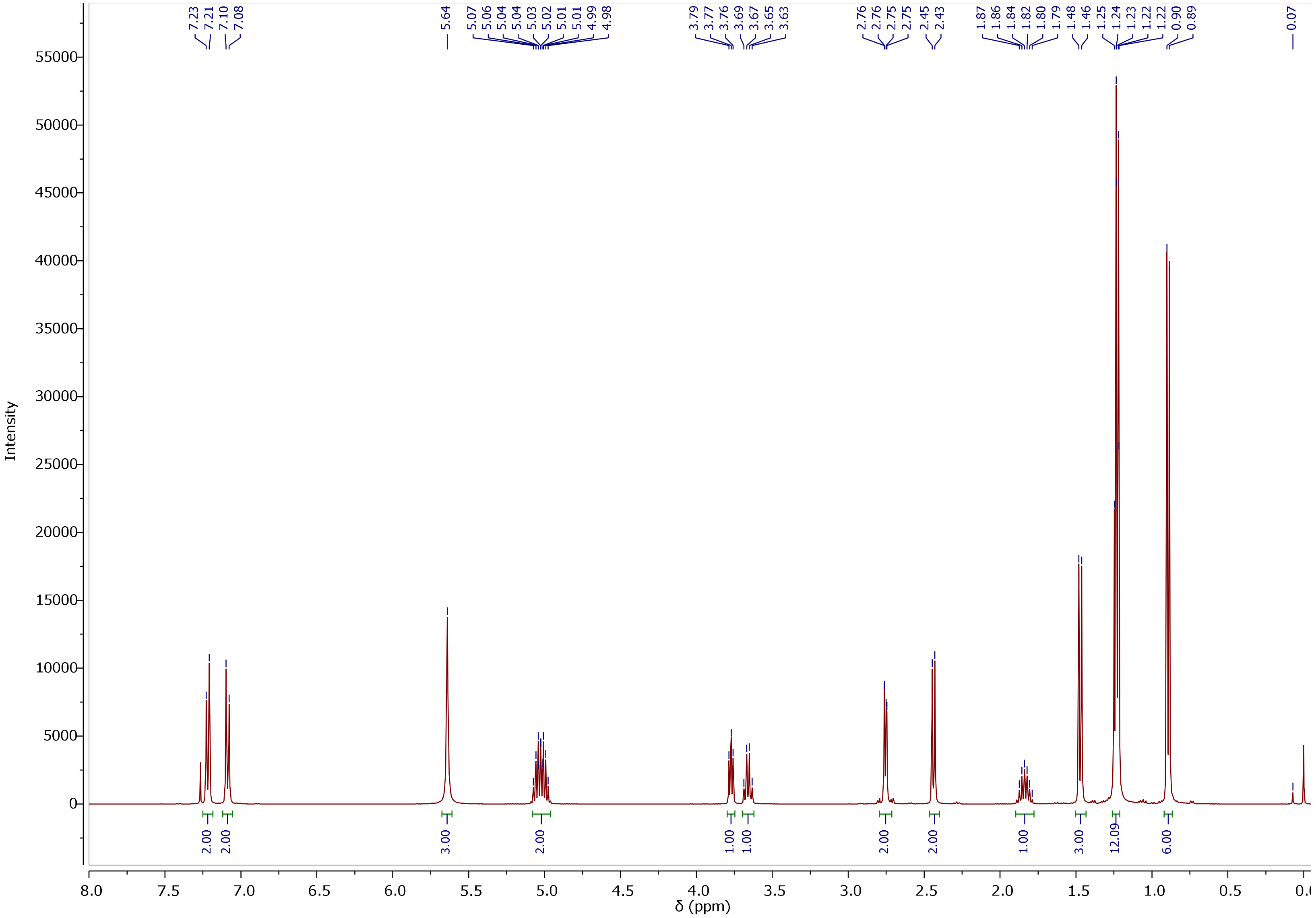

Figure S20. ^{1}H NMR spectra of [L-Asp(OiPr)$_2$][IBU].

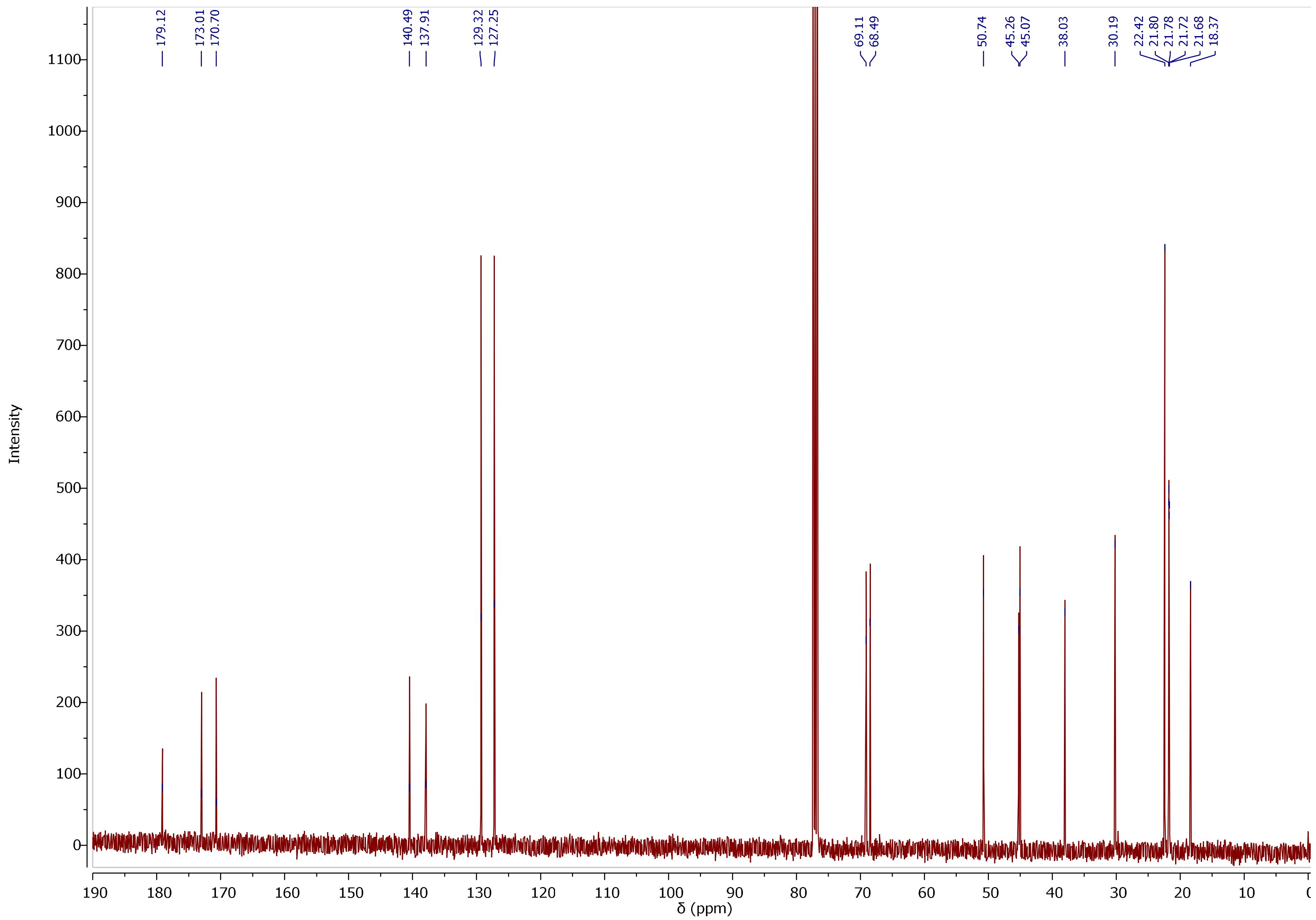

Figure S21. ^{13}C NMR spectra of [L-Asp(OiPr)$_2$][IBU].

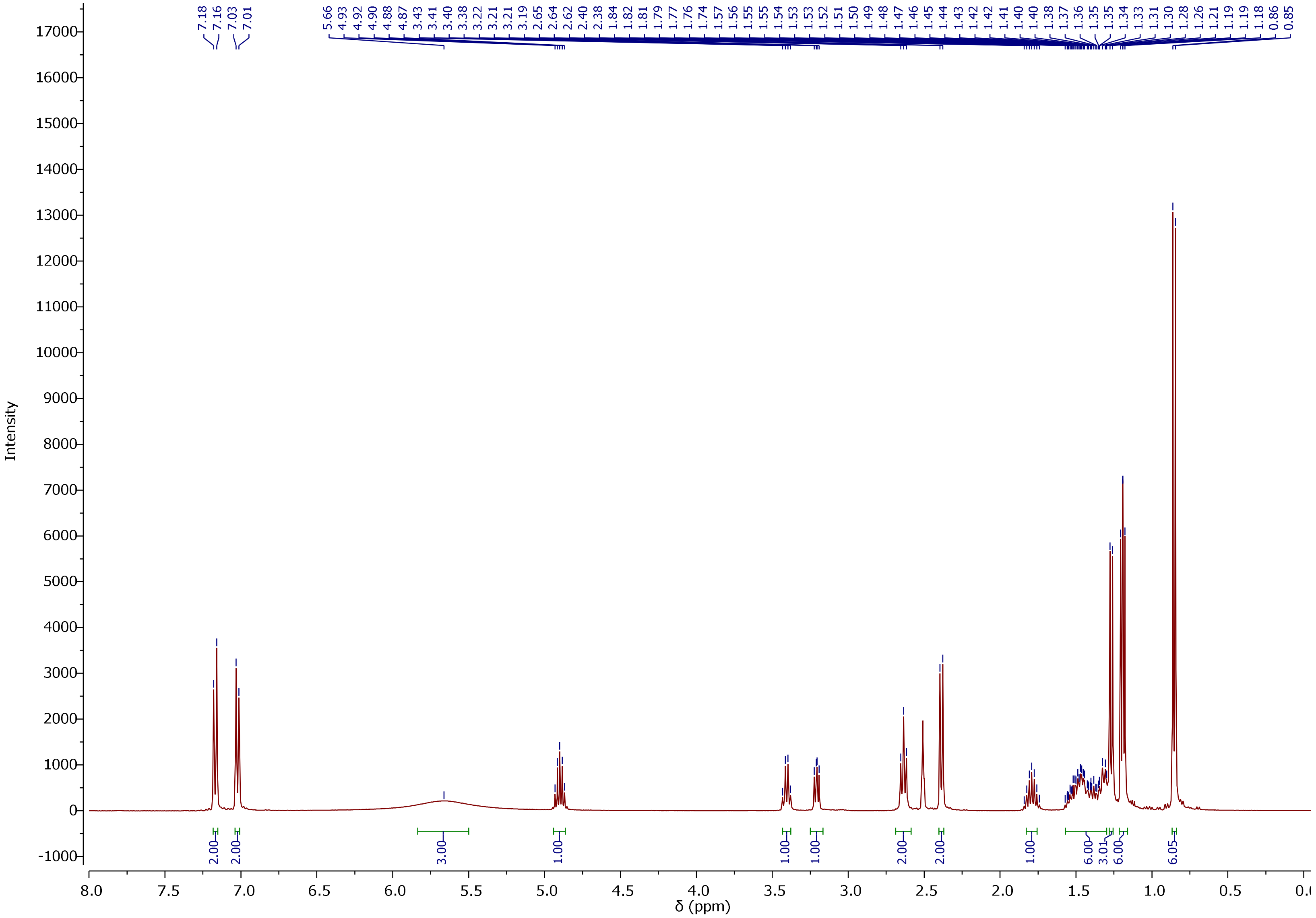

Figure S22. ^1H NMR spectra of [L-LysOiPr][IBU].

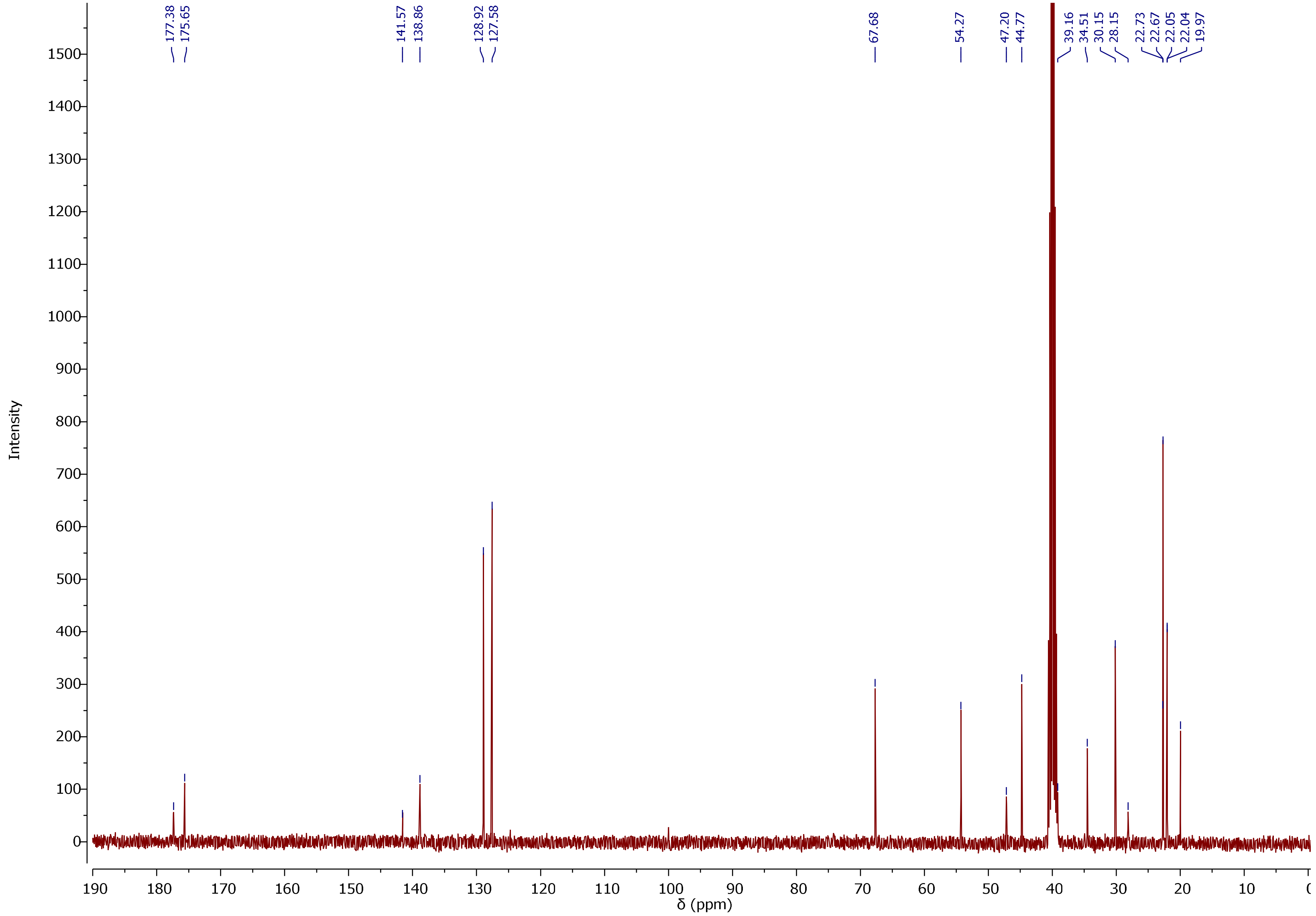

Figure S23. ^{13}C NMR spectra of [L-LysOiPr][IBU].

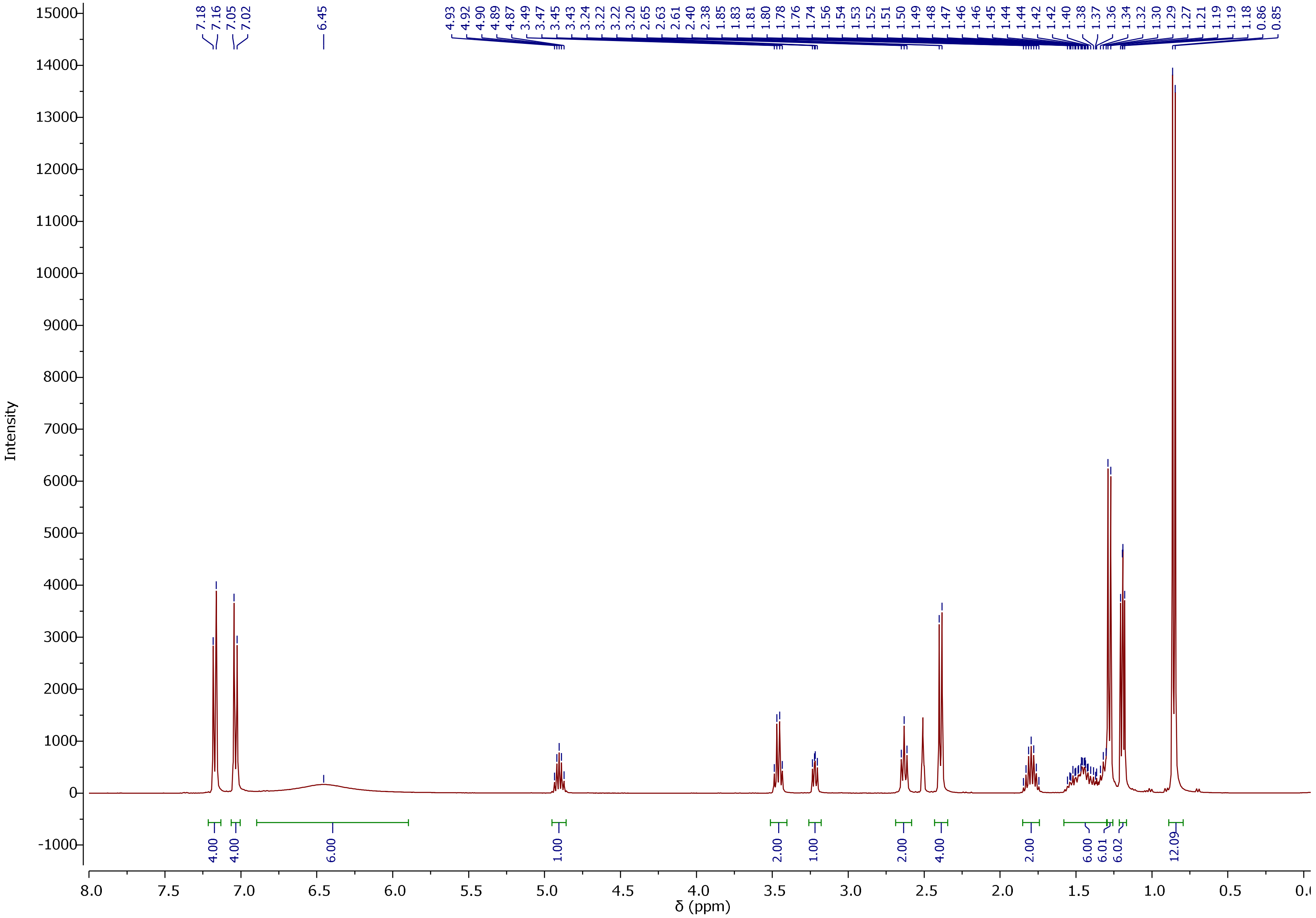

Figure S24. ^{1}H NMR spectra of [L-LysOiPr][IBU]$_2$.

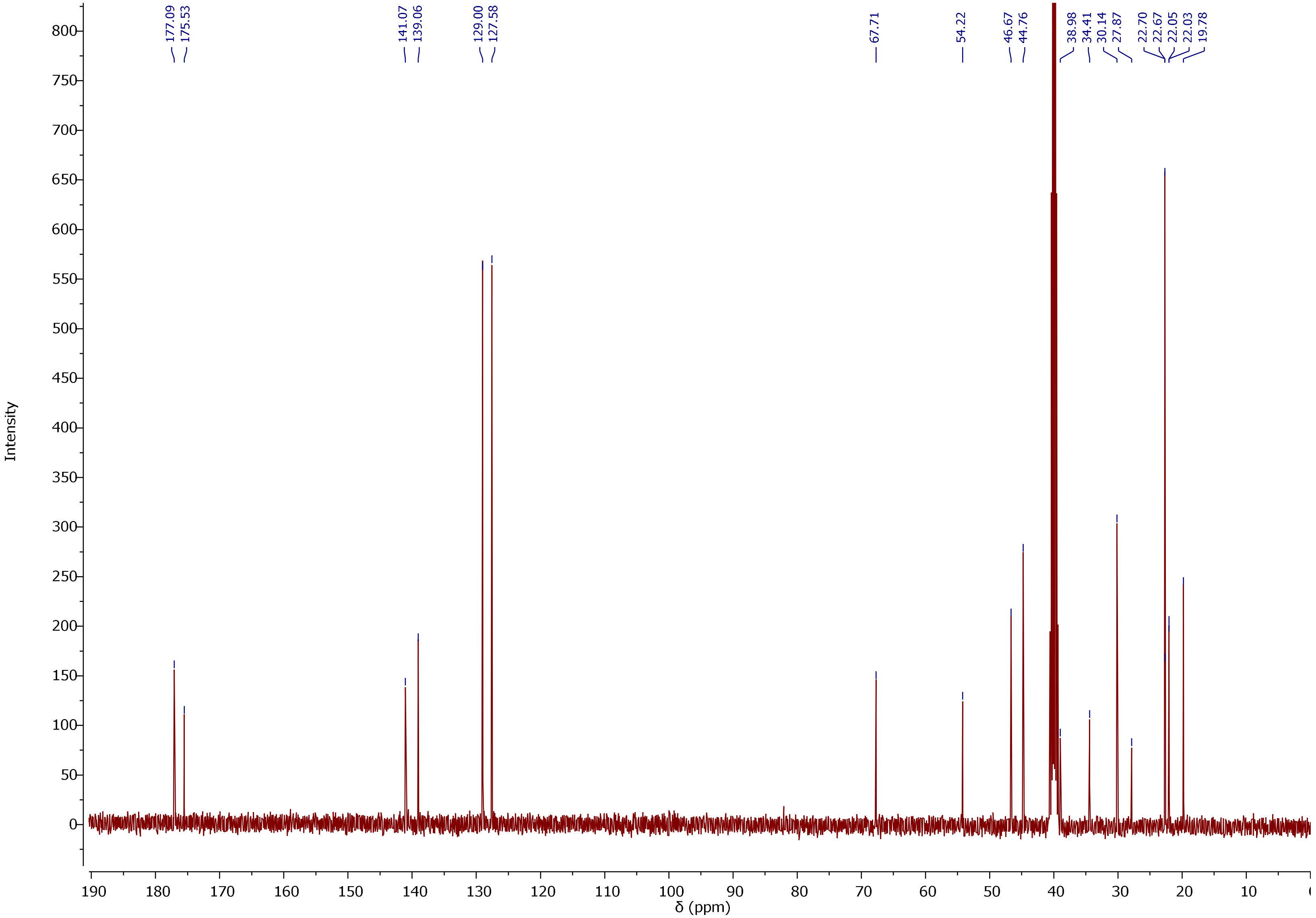

Figure S25. ^{13}C NMR spectra of [L-LysOiPr][IBU]$_2$.

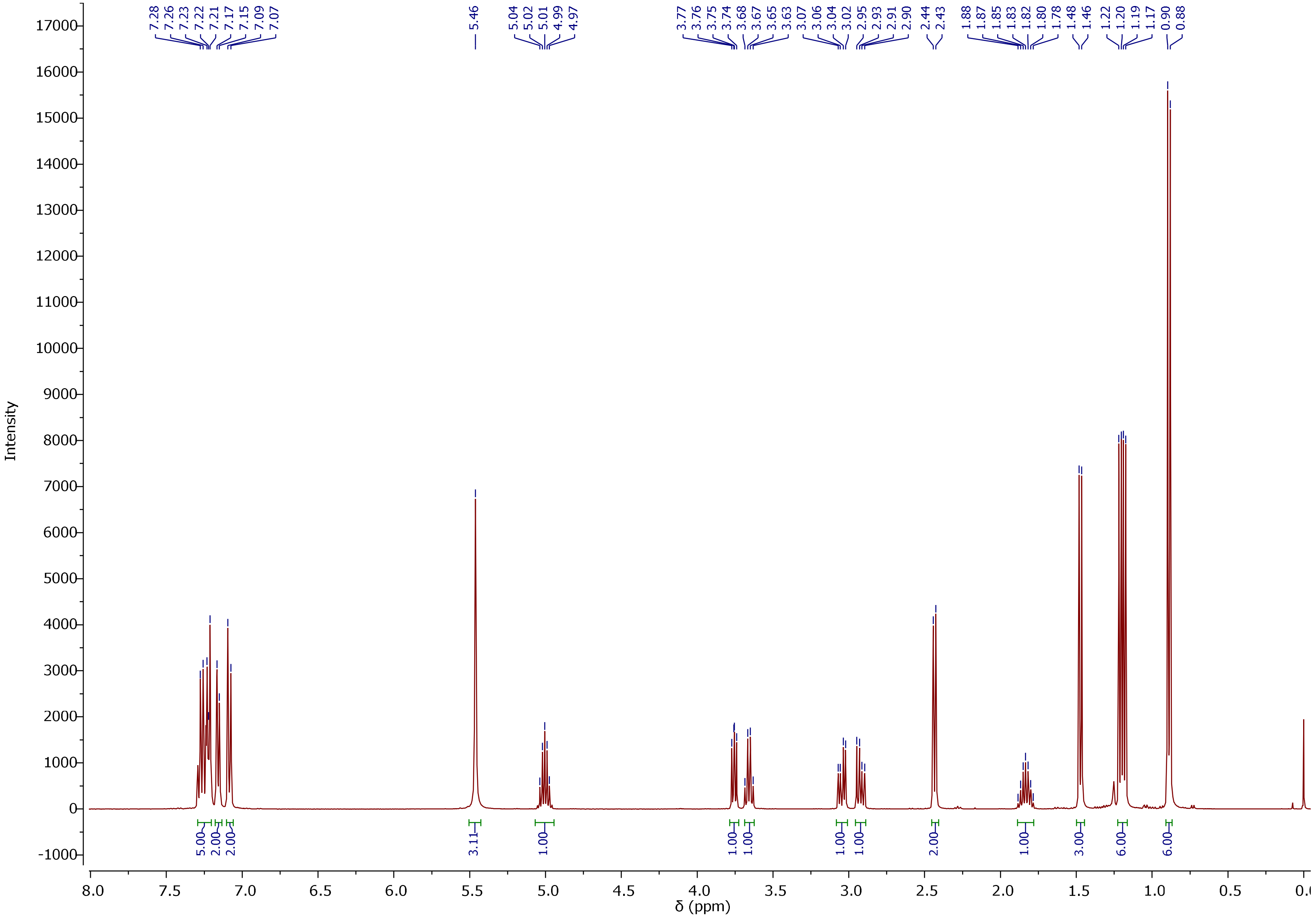

Figure S26. ^{1}H NMR spectra of [L-PheOiPr][IBU].

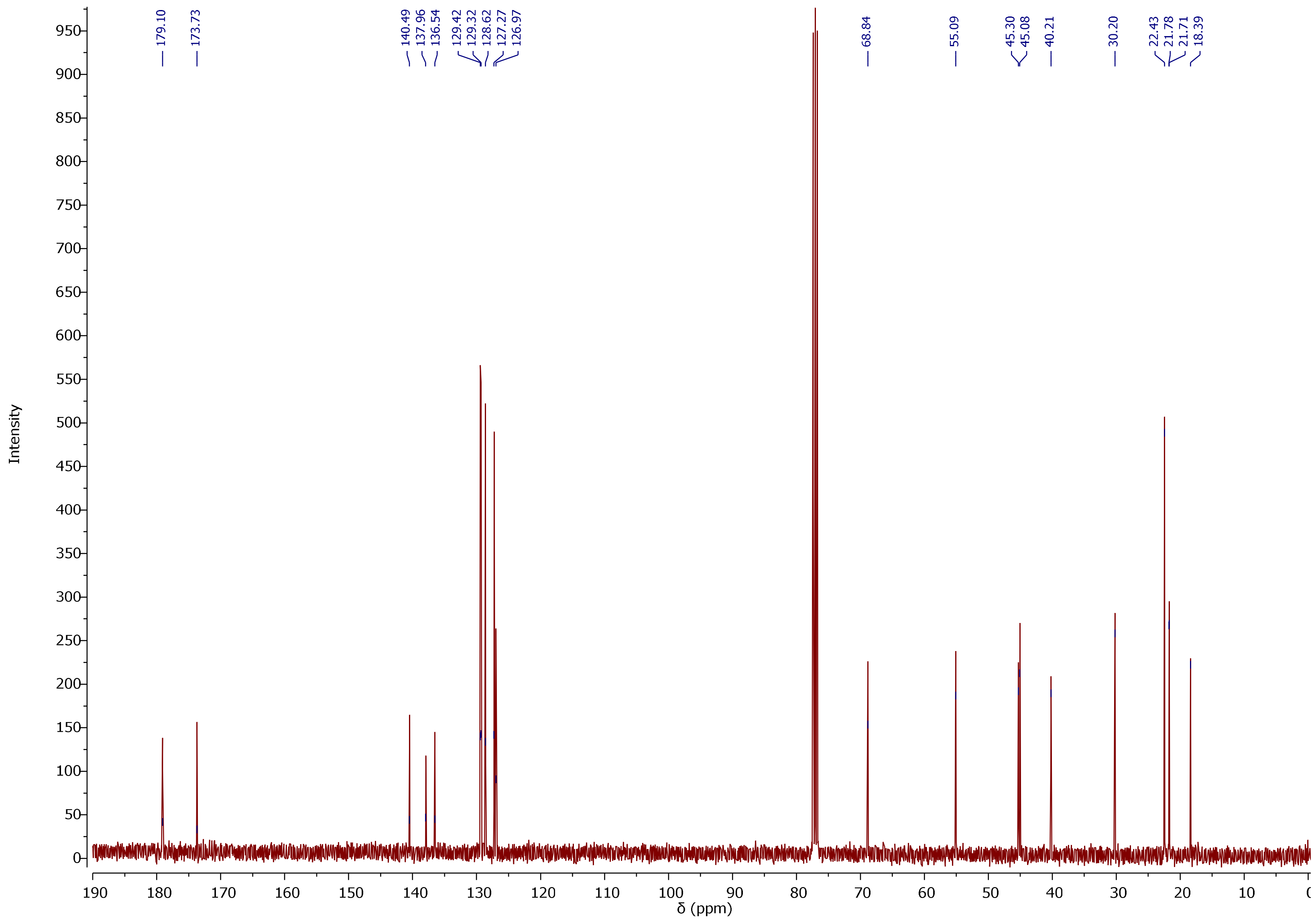

Figure S27. ^{13}C NMR spectra of [L-PheOiPr][IBU].

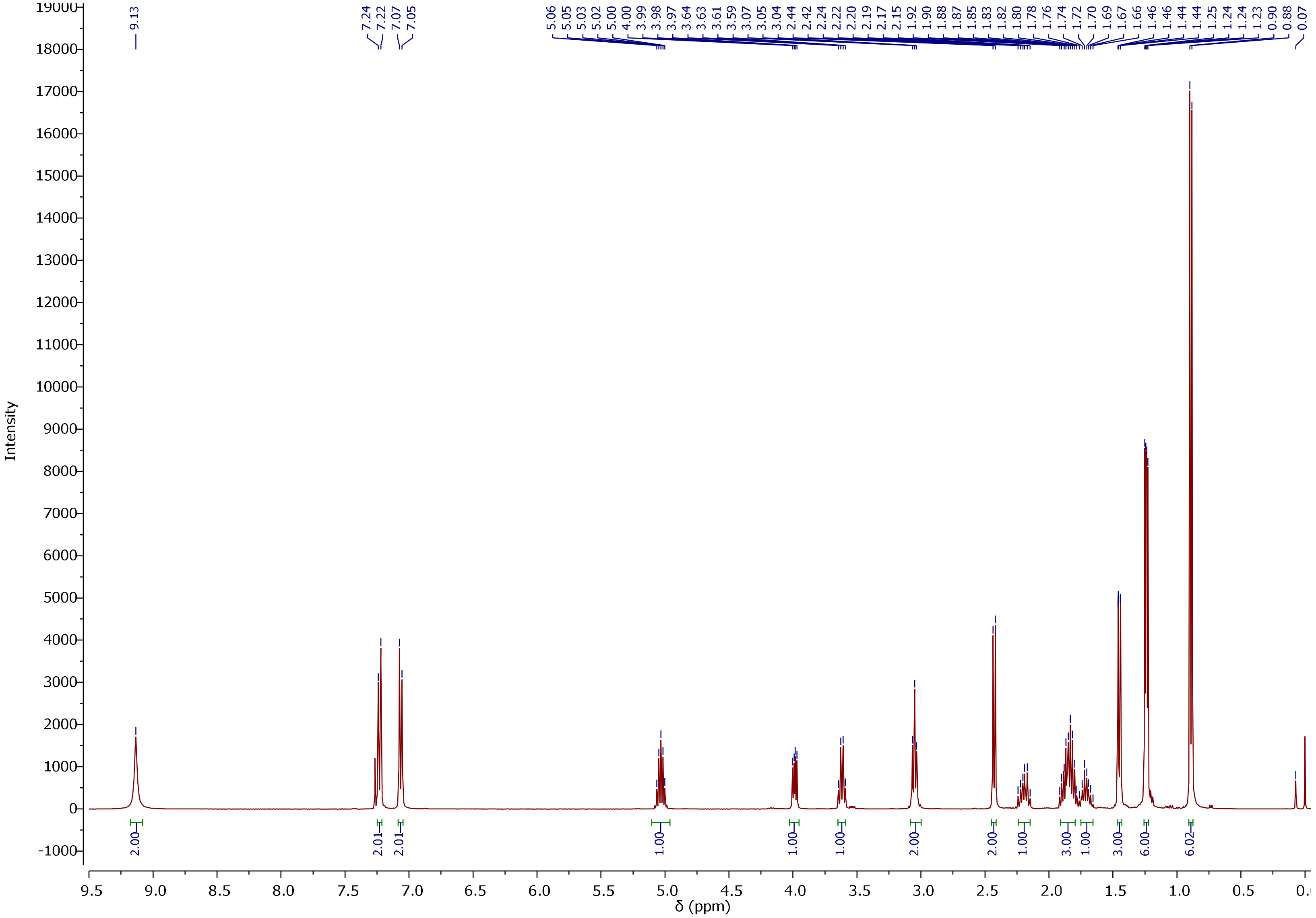

Figure S28. ^1H NMR spectra of [L-ProOiPr][IBU].

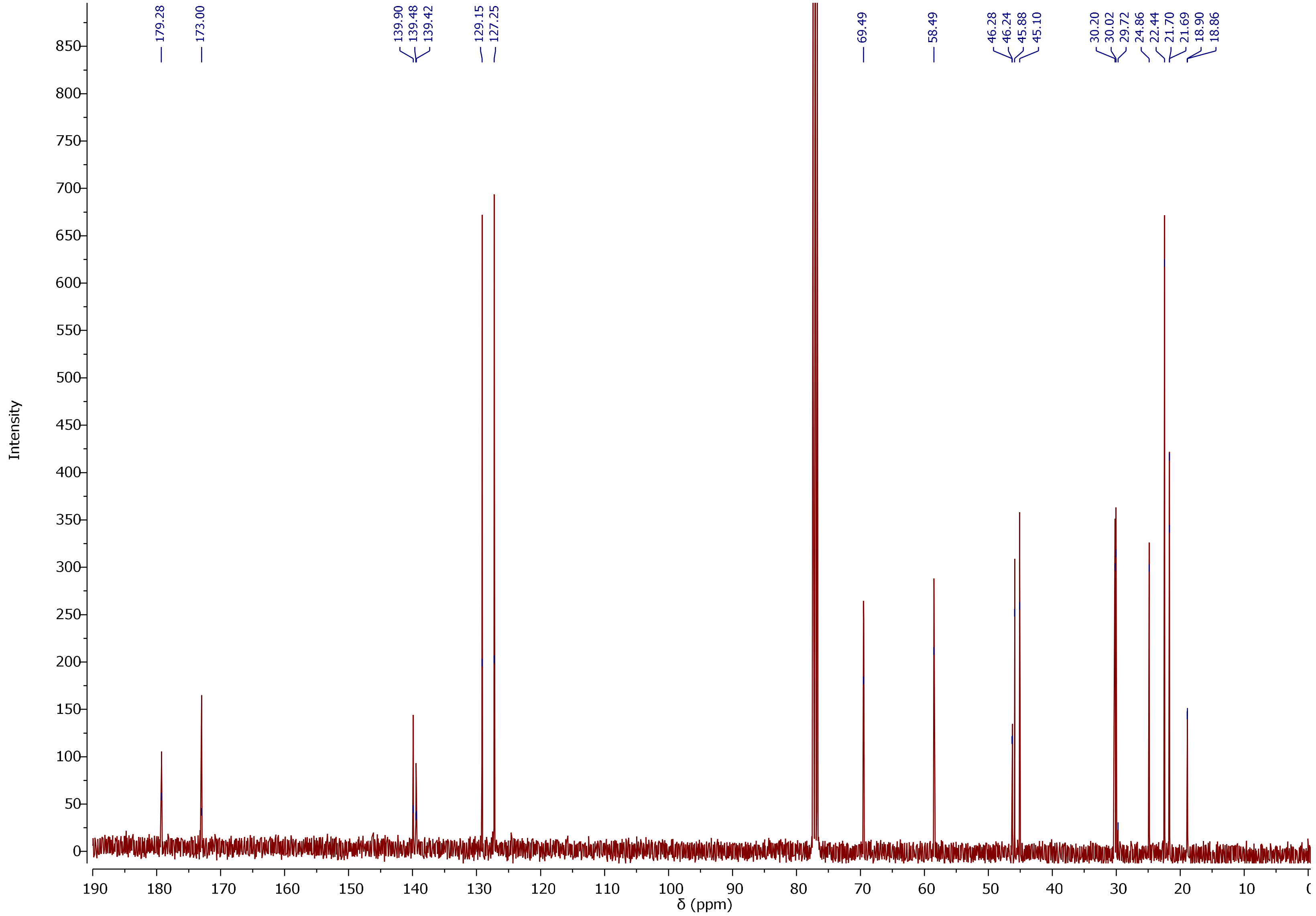

Figure S29. ^{13}C NMR spectra of [L-ProOiPr][IBU].

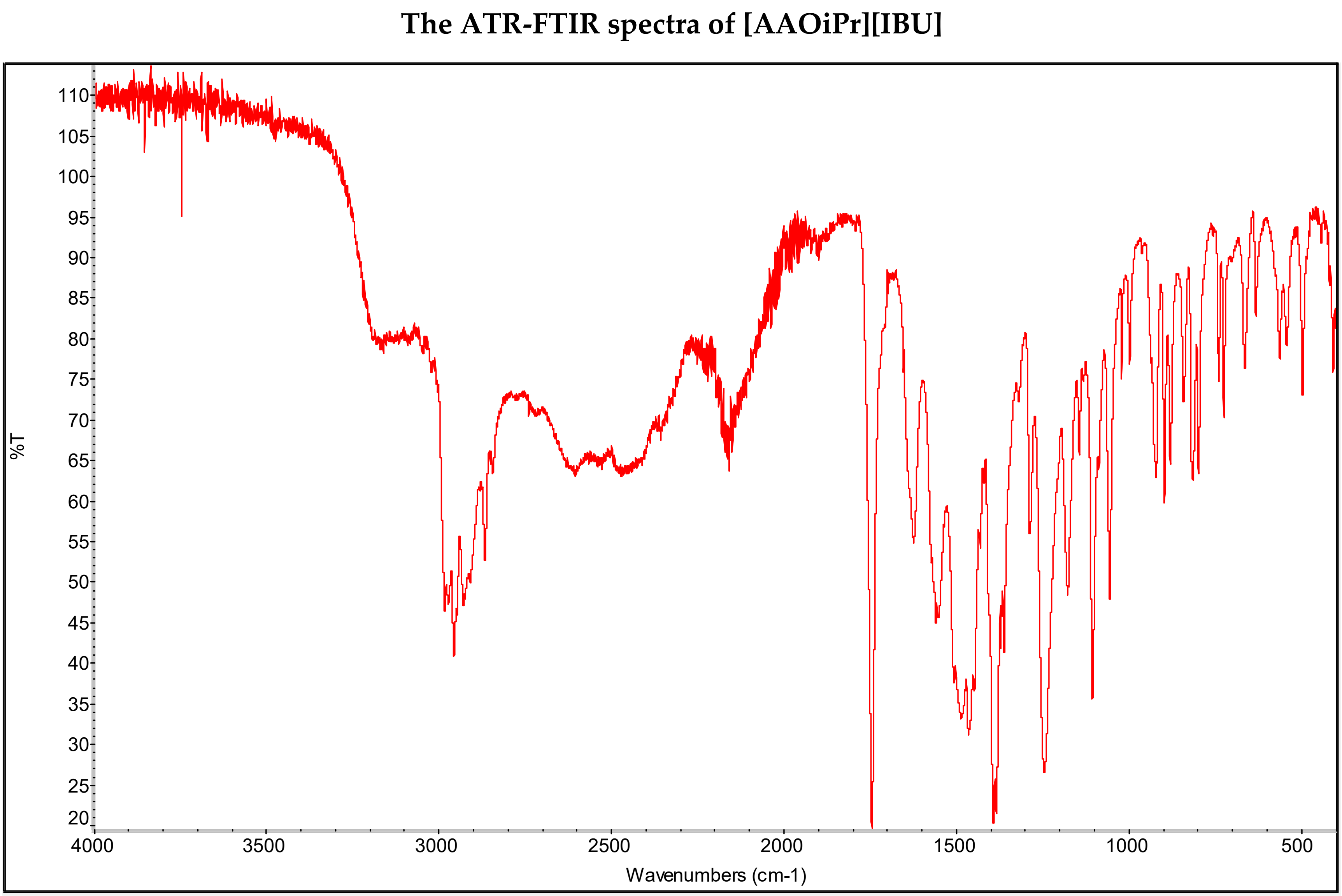

Figure S30. ATR-FTIR spectra of [GlyOiPr][IBU].

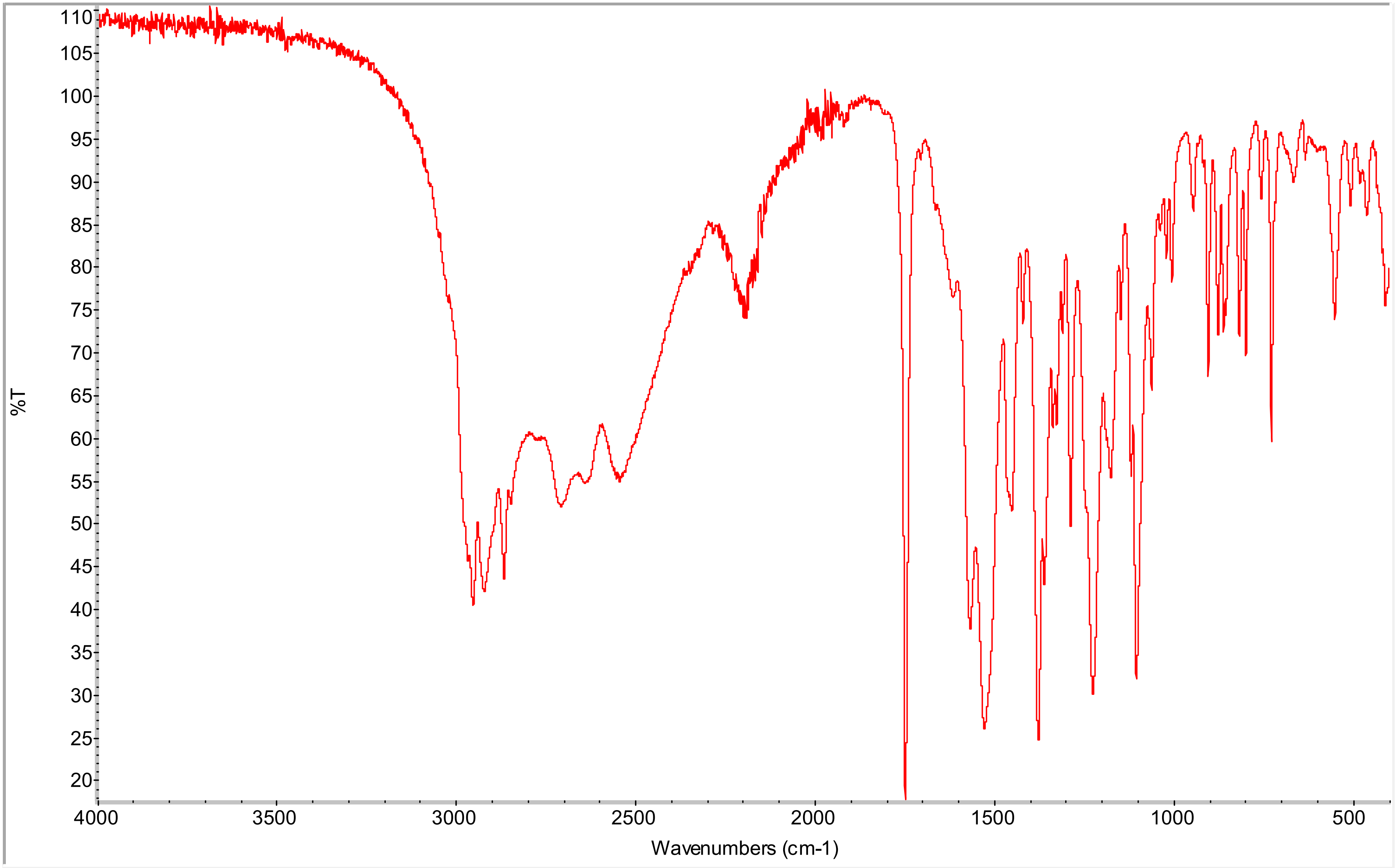

Figure S31. ATR-FTIR spectra of [L-AlaOiPr][IBU].

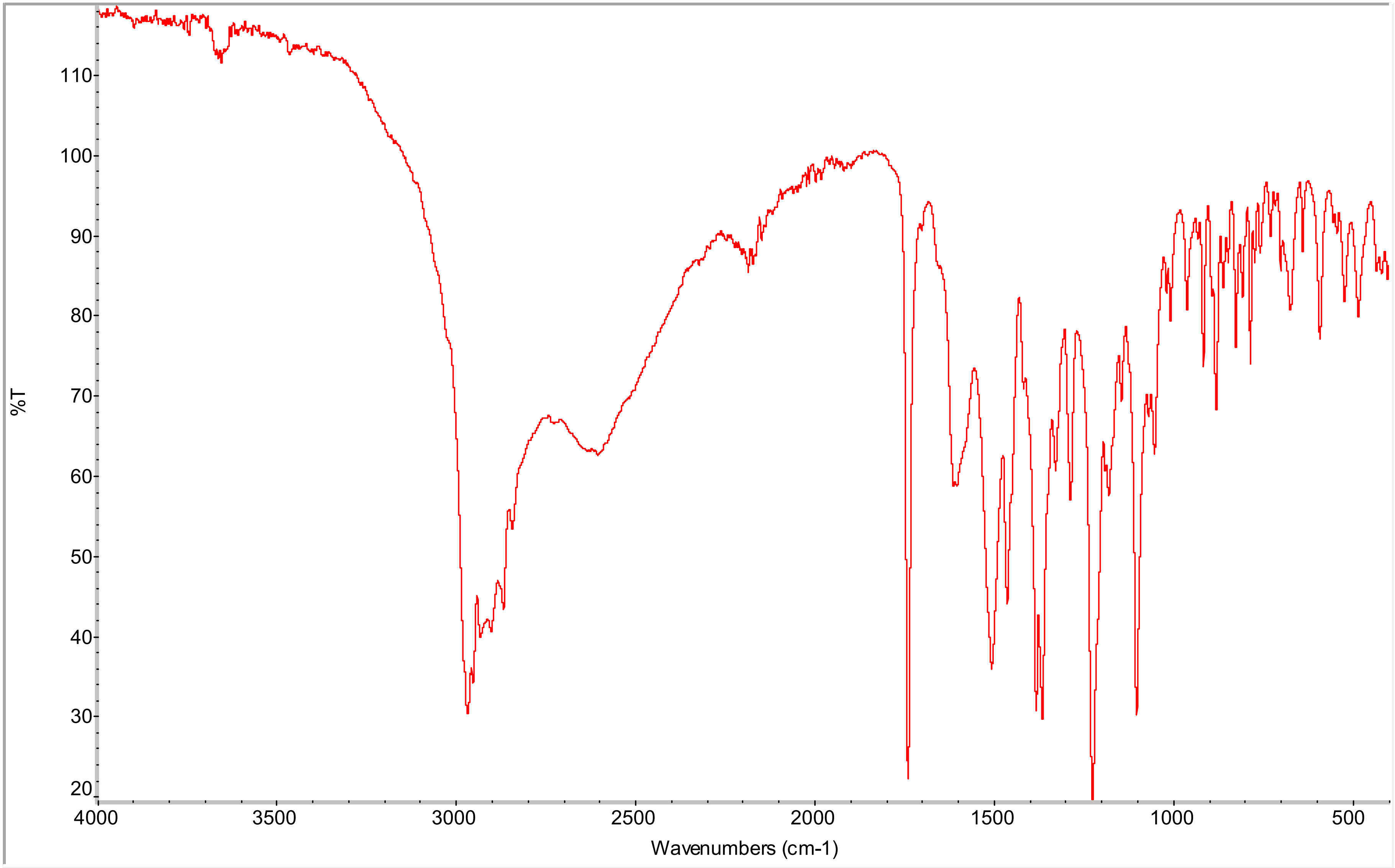

Figure S32. ATR-FTIR spectra of [L-ValOiPr][IBU].

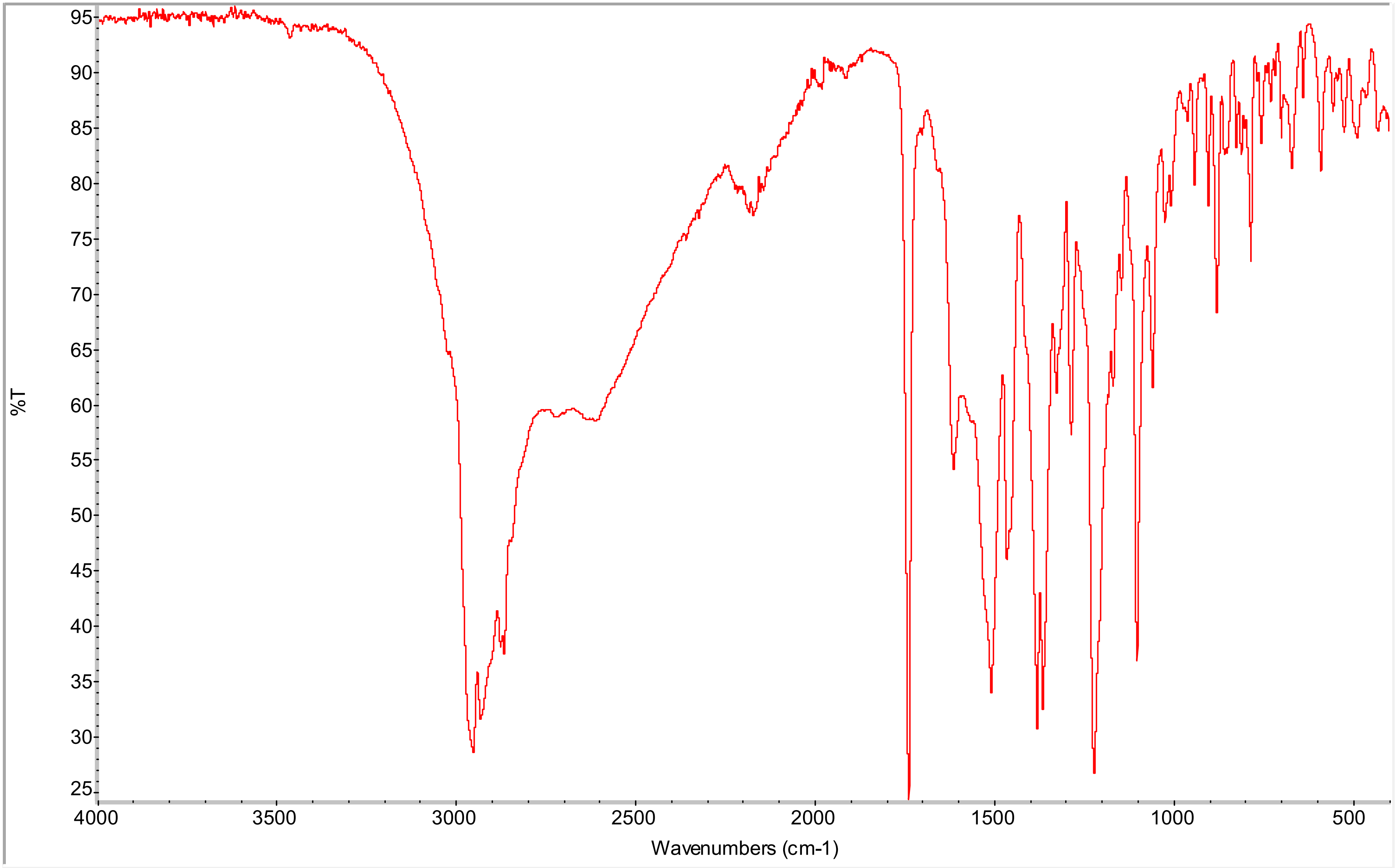

Figure S33. ATR-FTIR spectra of [L-IleOiPr][IBU].

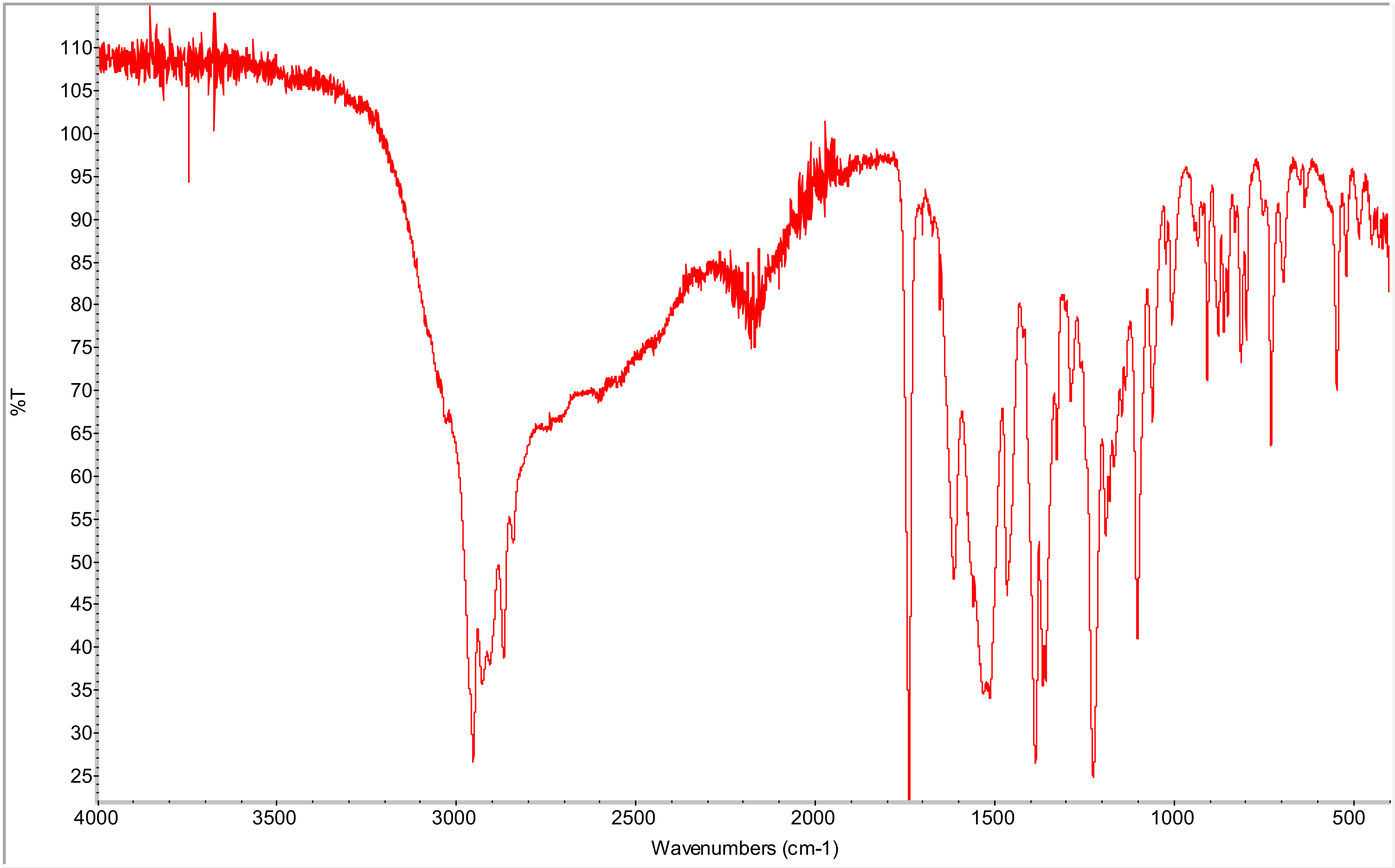

Figure S34. ATR-FTIR spectra of [L-LeuOiPr][IBU].

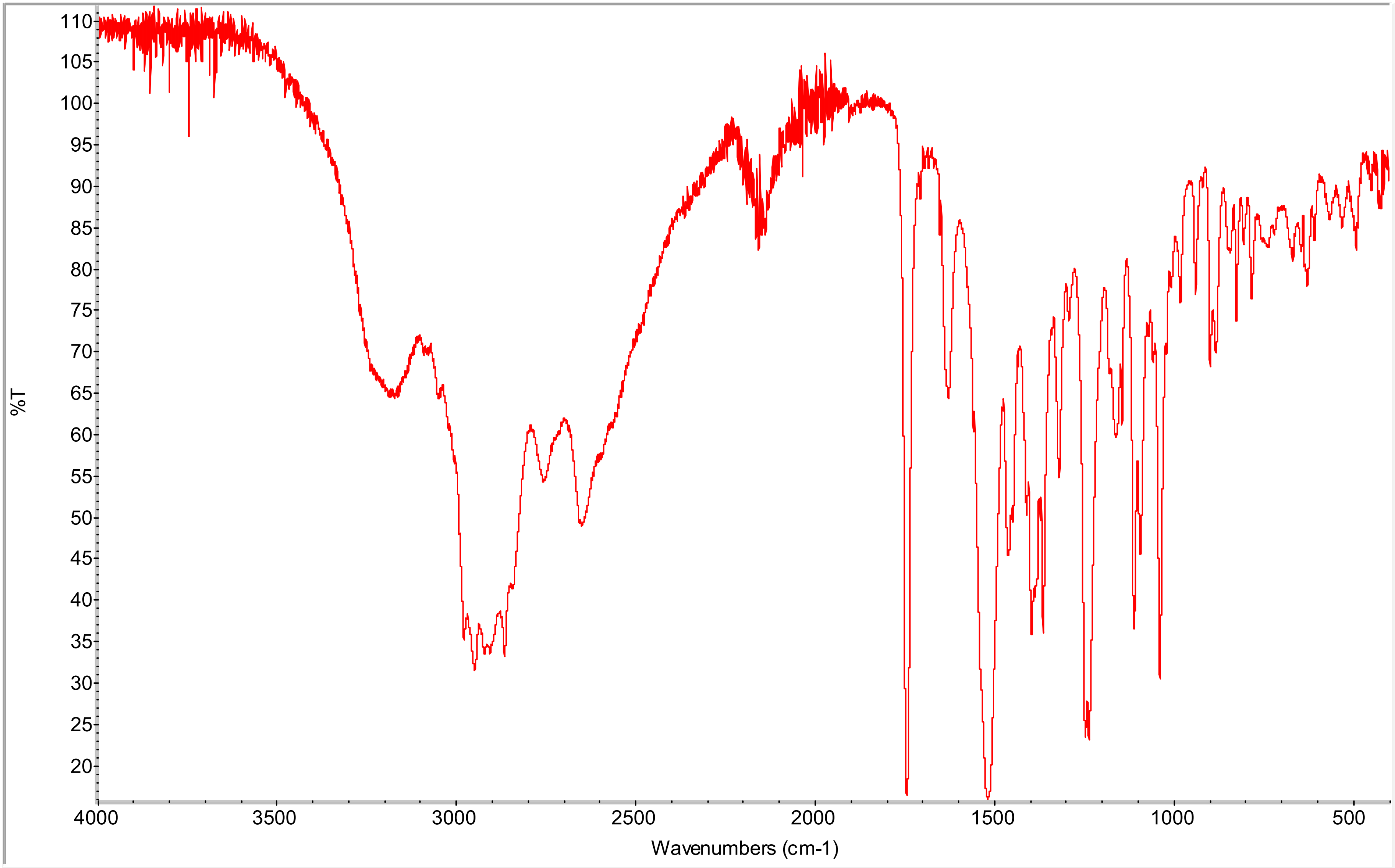

Figure S35. ATR-FTIR spectra of [L-SerOiPr][IBU].

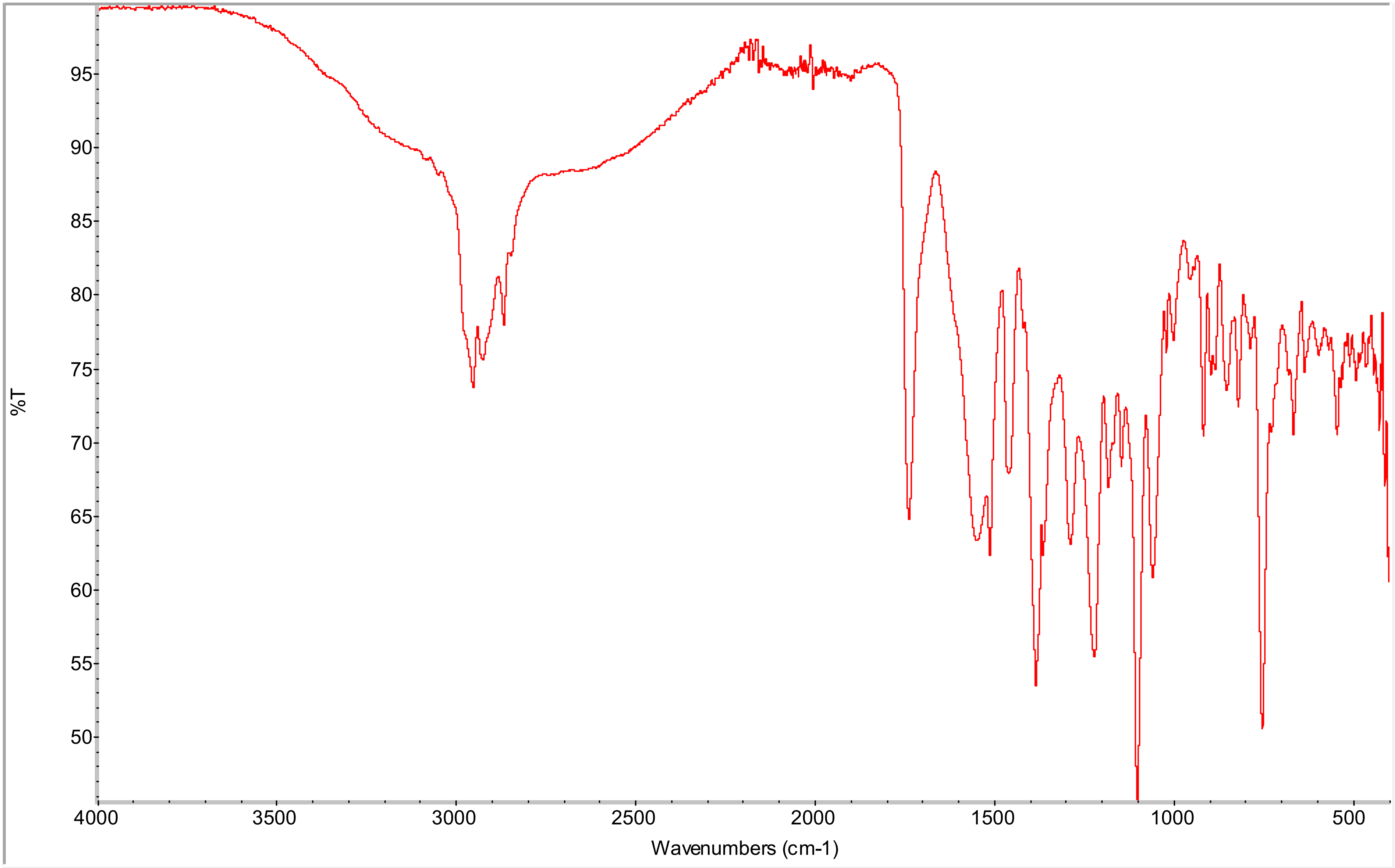

Figure S36. ATR-FTIR spectra of [L-ThrOiPr][IBU].

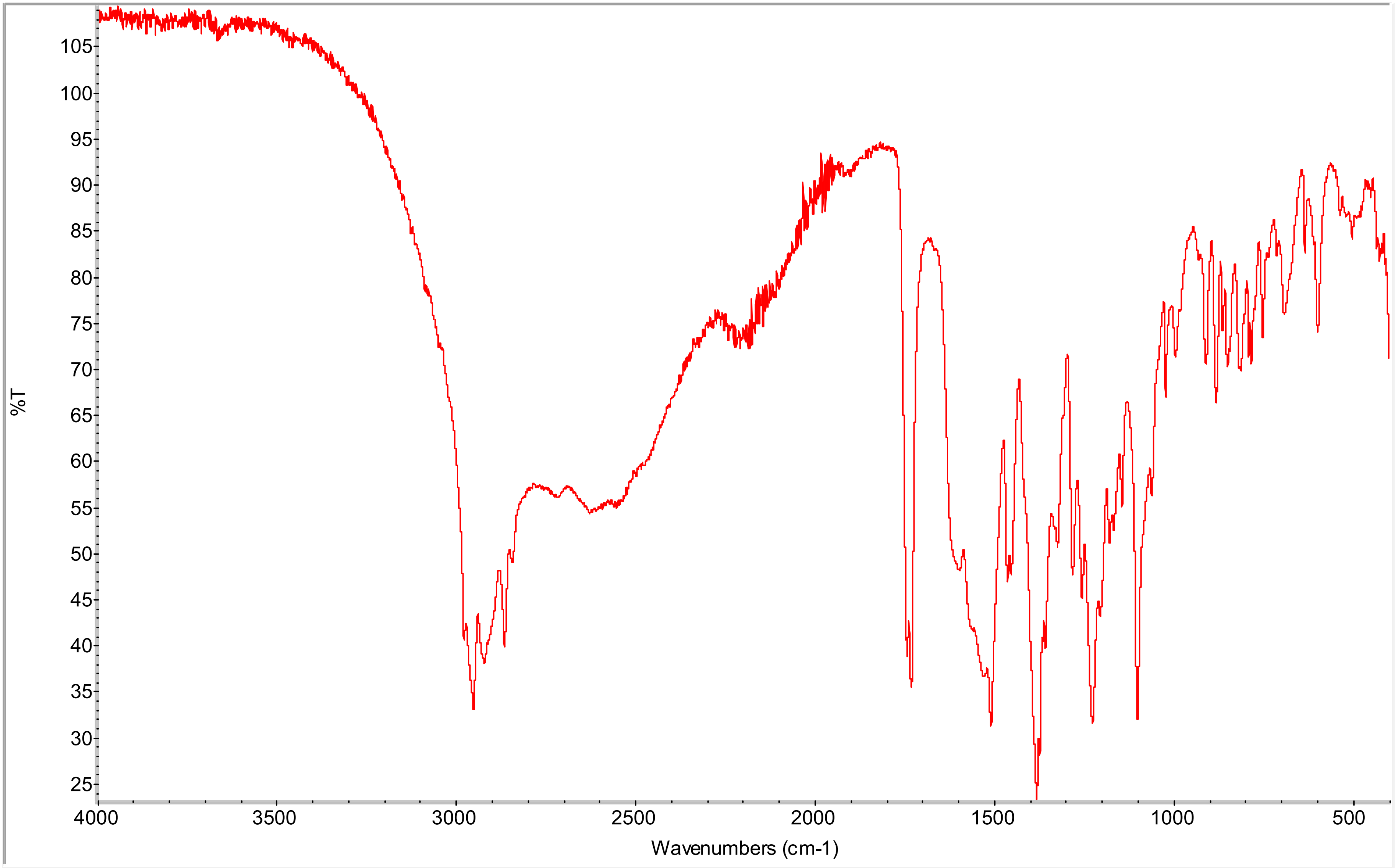

Figure S37. ATR-FTIR spectra of [L-CysOiPr][IBU].

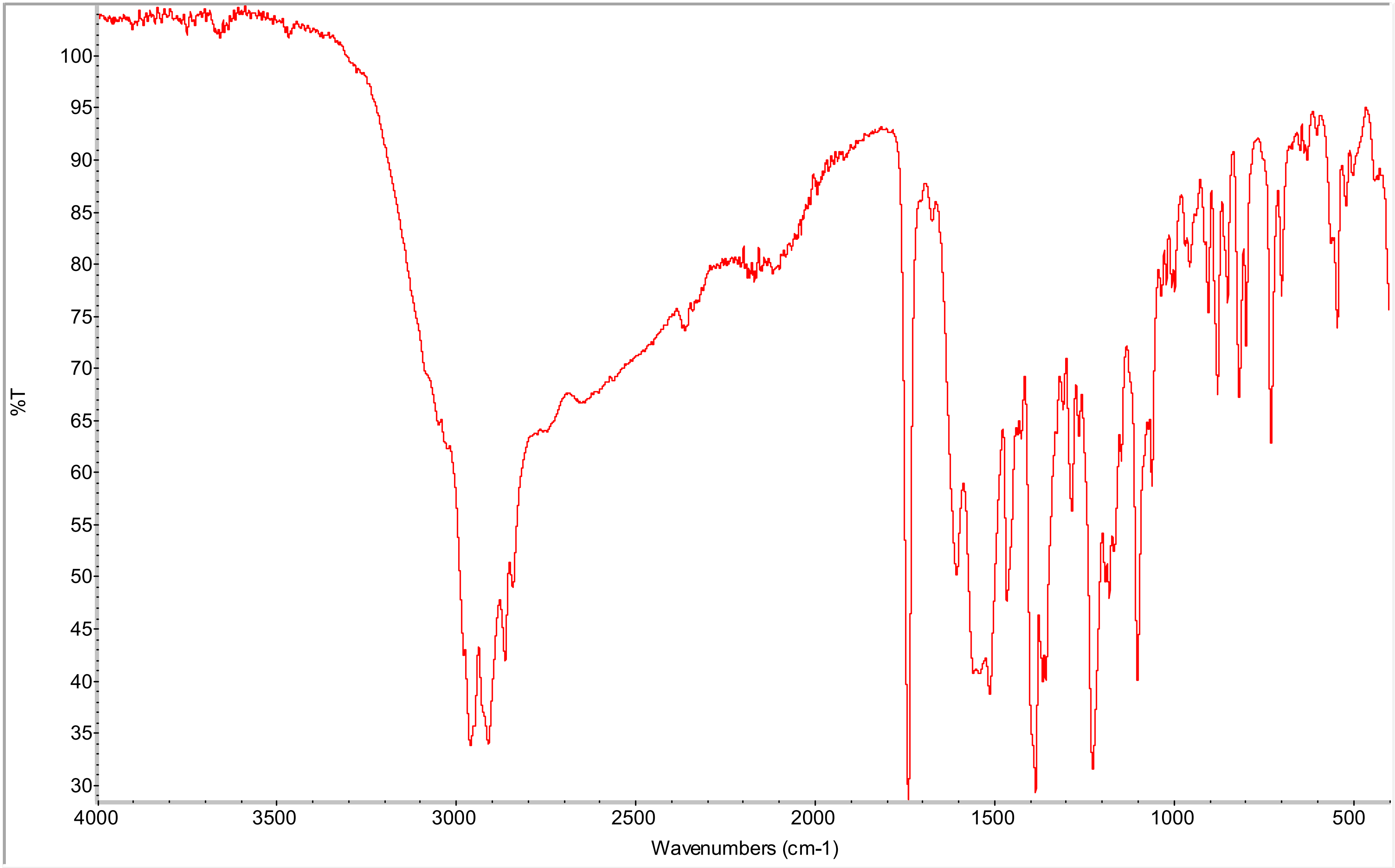

Figure S38. ATR-FTIR spectra of [L-MetOiPr][IBU].

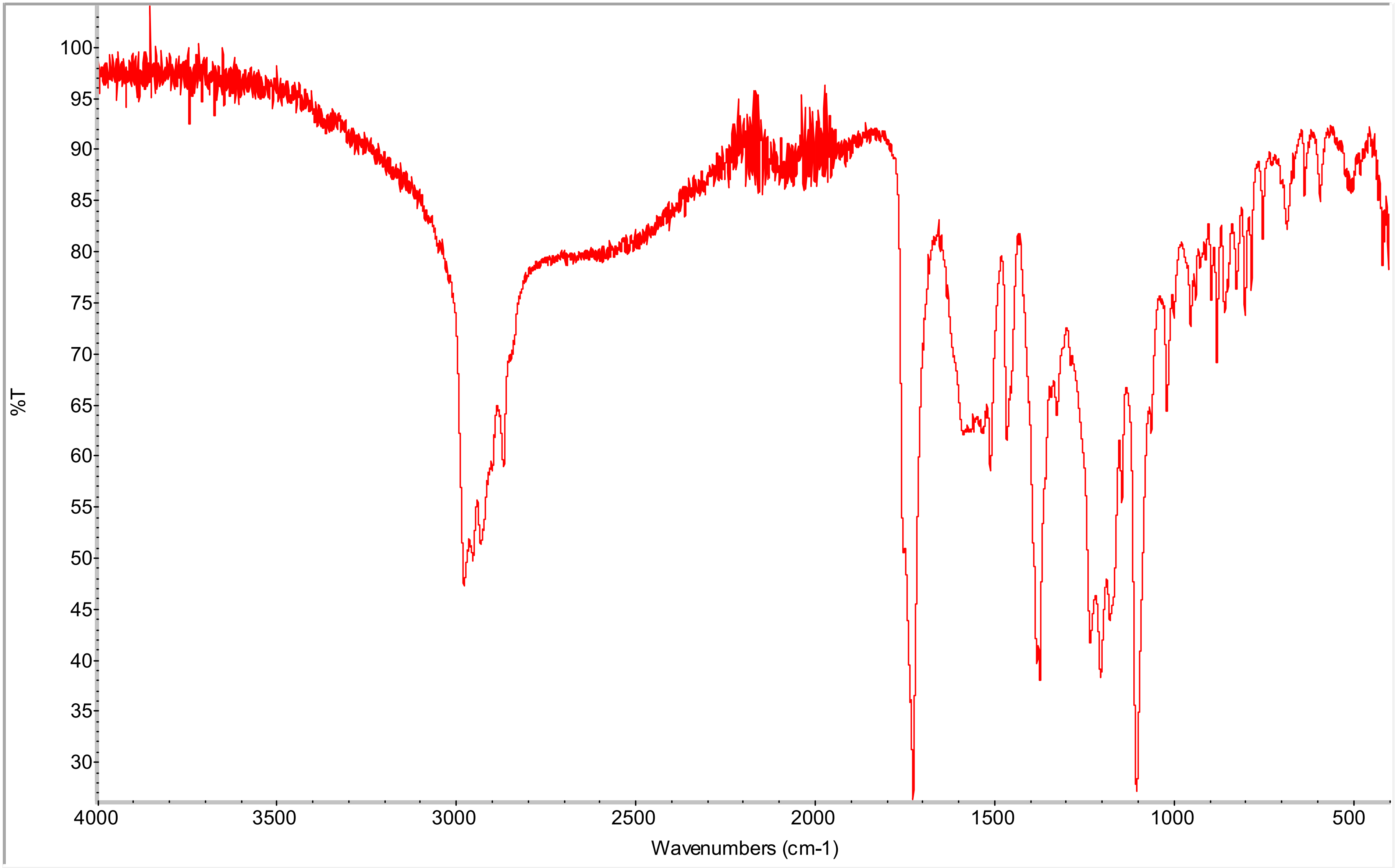

Figure S39. ATR-FTIR spectra of [L-Asp(OiPr)$_2$][IBU].

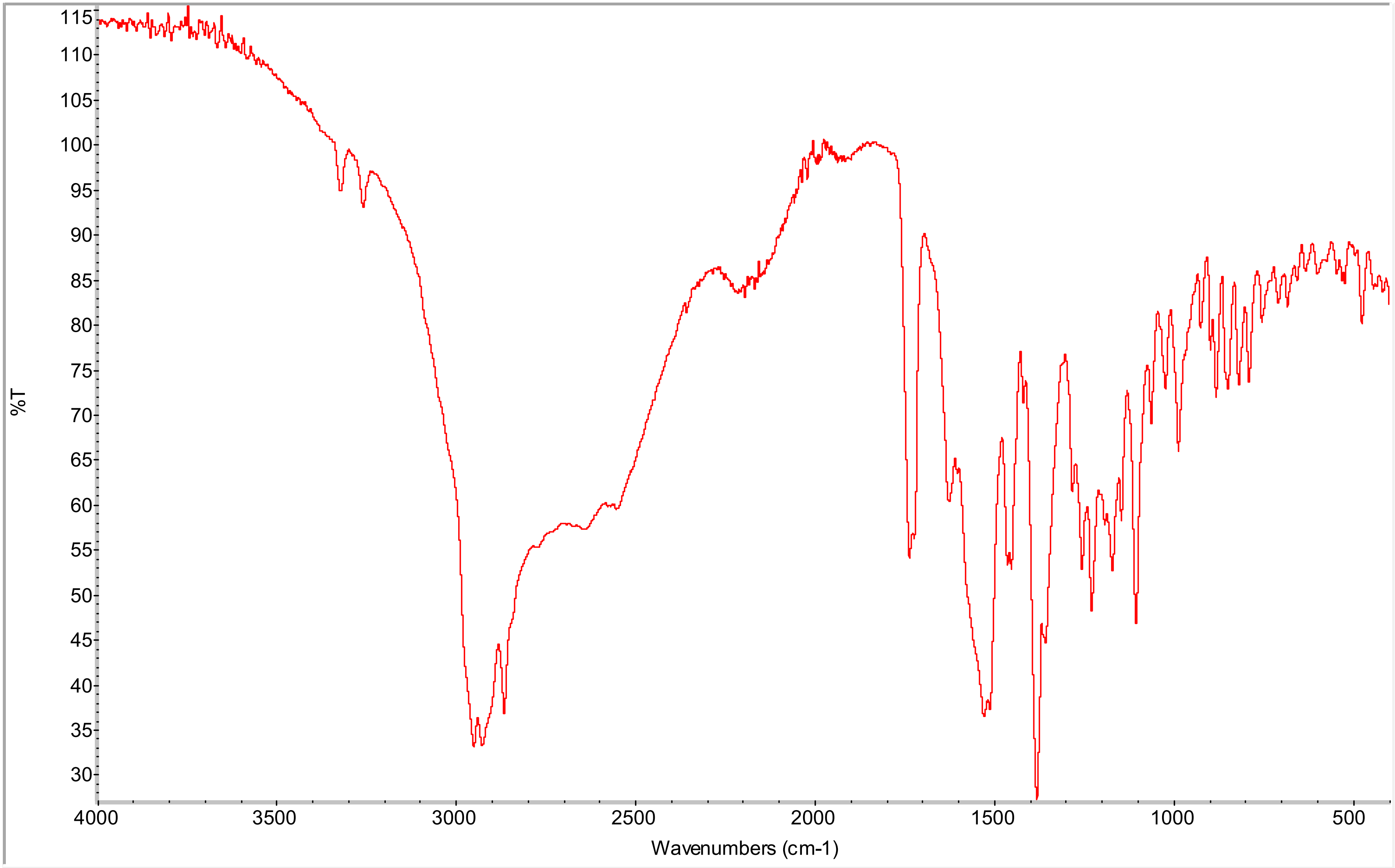

Figure S40. ATR-FTIR spectra of [L-LysOiPr][IBU].

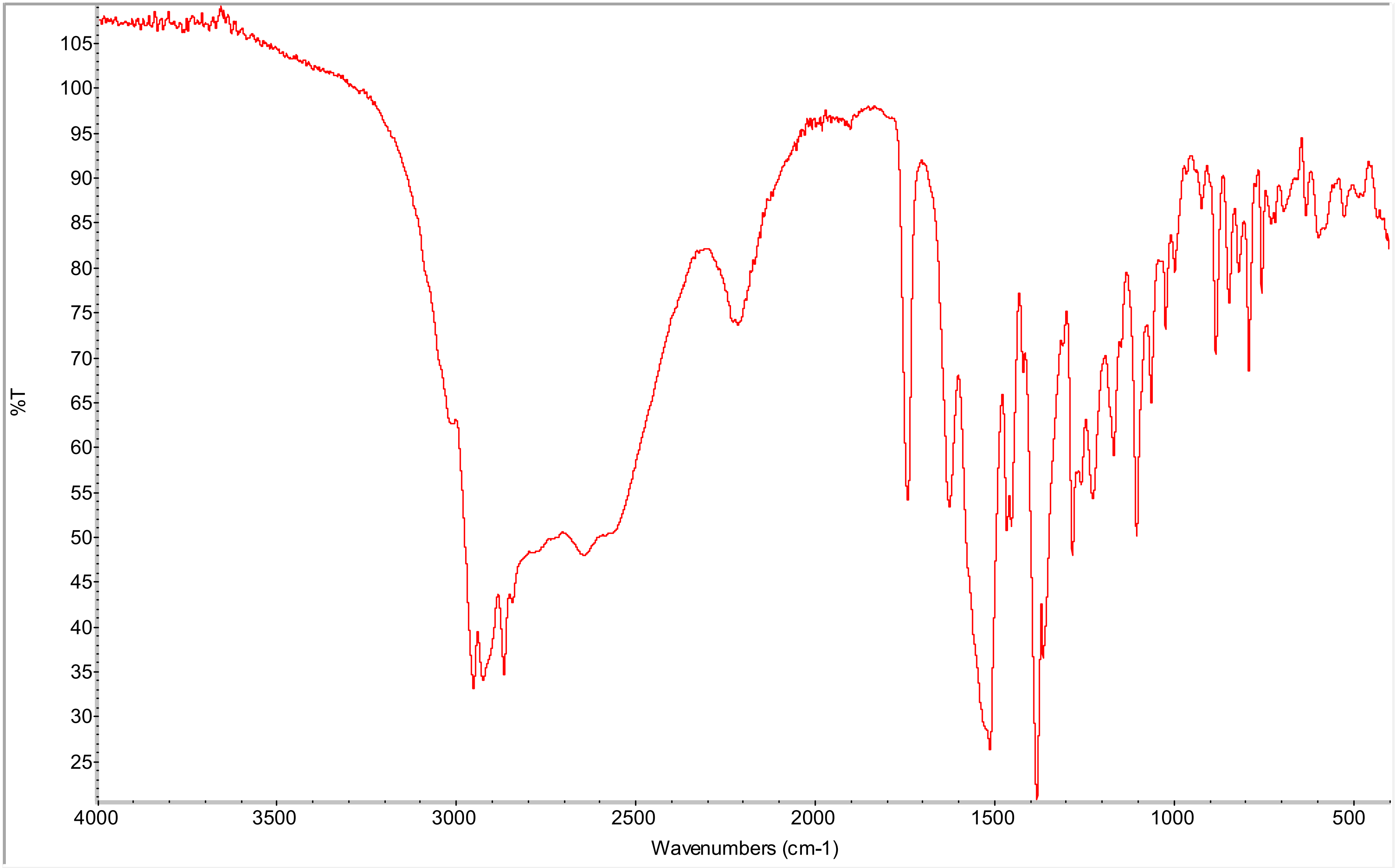

Figure S41. ATR-FTIR spectra of [L-LysOiPr][IBU]2.

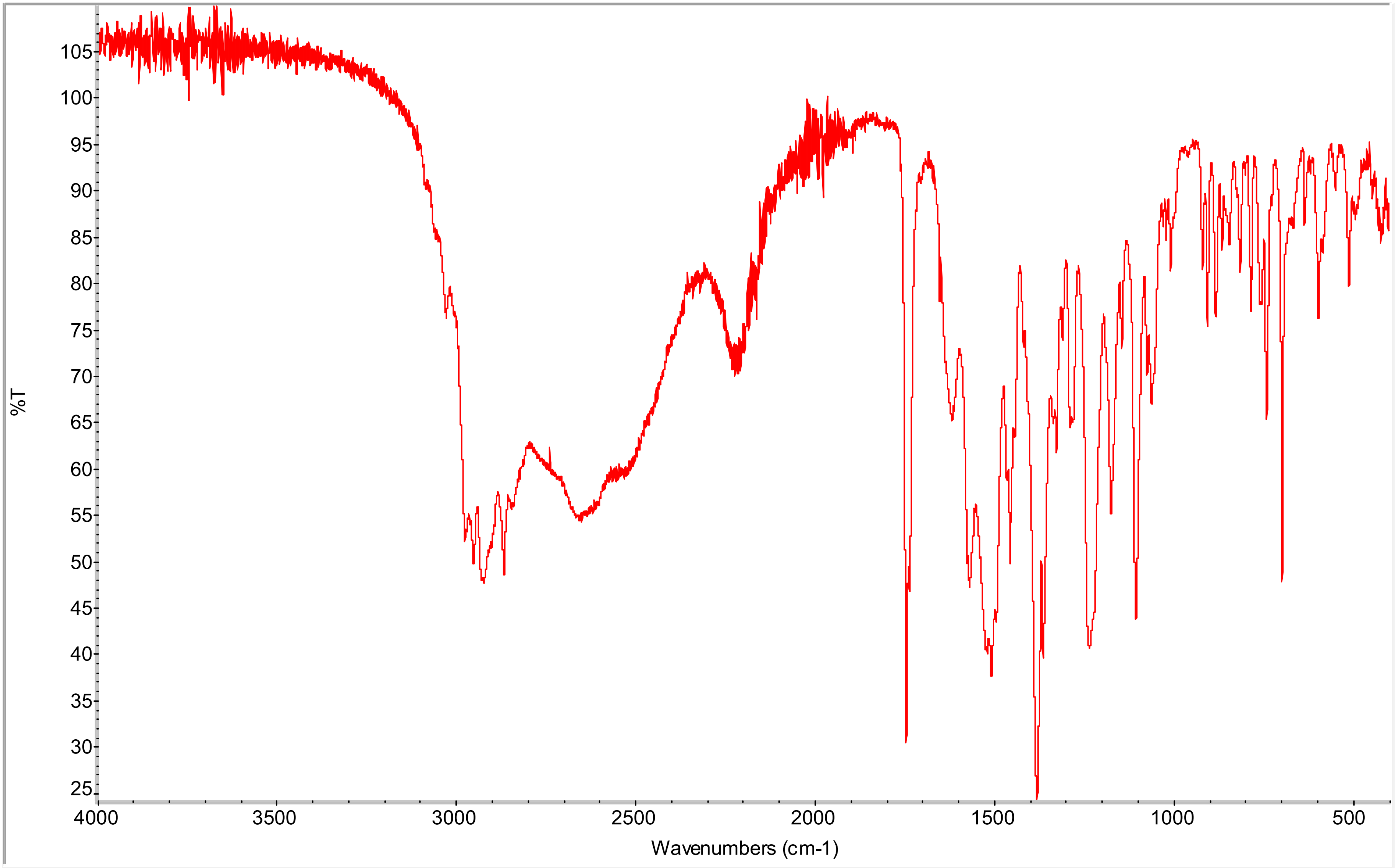

Figure S42. ATR-FTIR spectra of [L-PheOiPr][IBU].

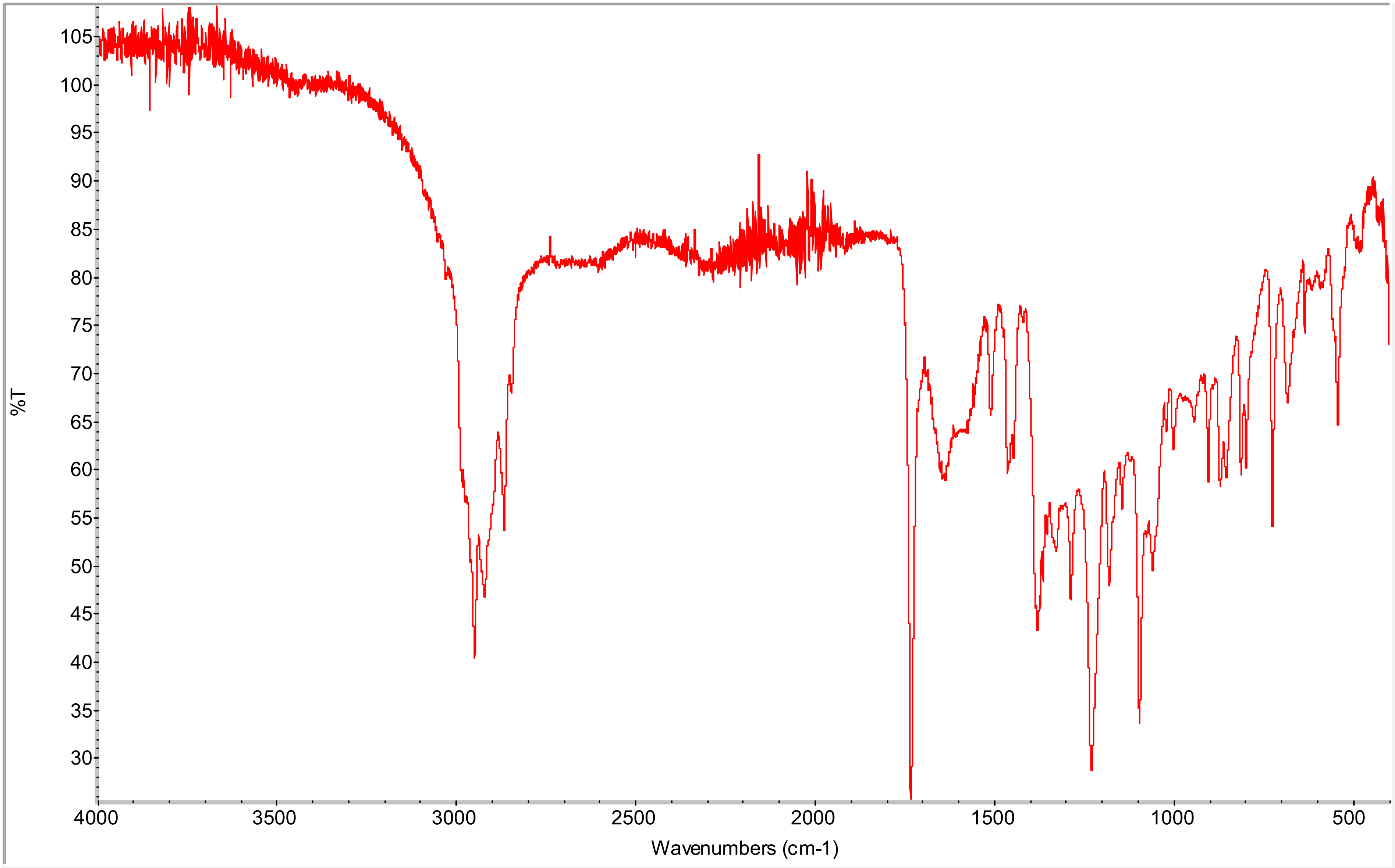

Figure S43. ATR-FTIR spectra of [L-ProOiPr][IBU].

The UV-Vis spectra of [AAOiPr][IBU]

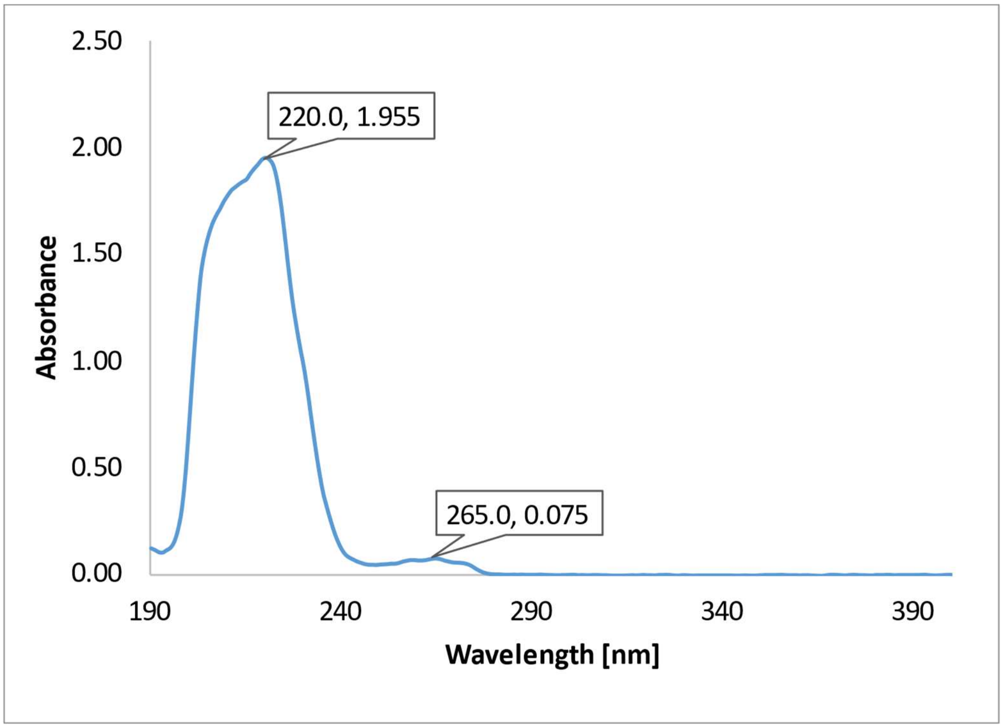

Figure S44. UV-Vis spectra of [GlyOiPr][IBU].

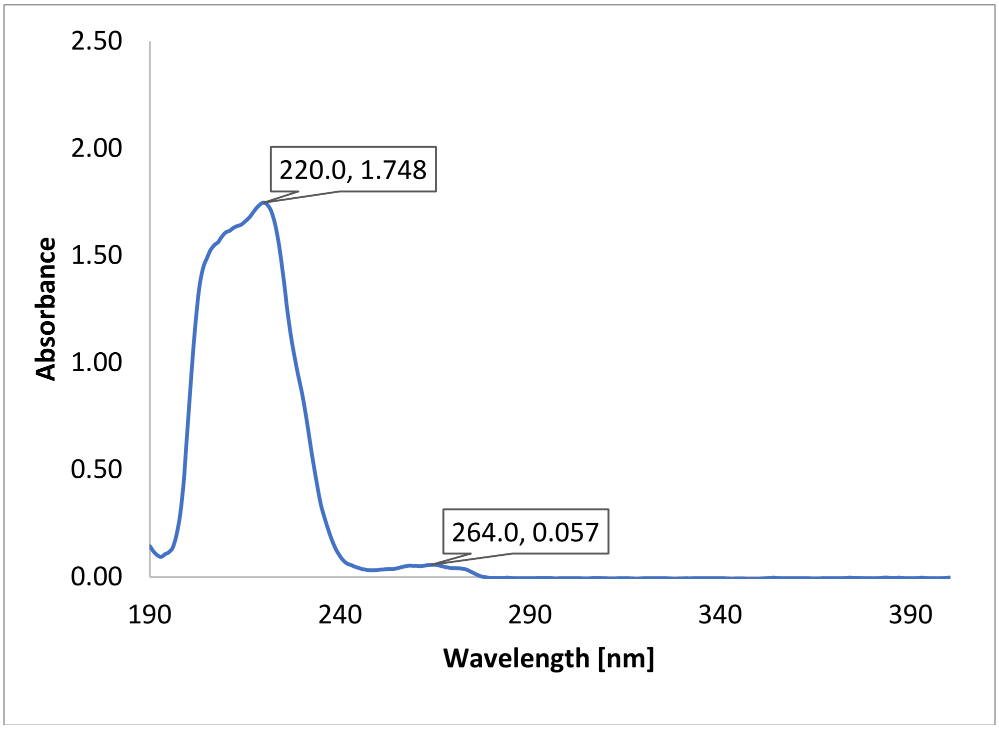

Figure S45. UV-Vis spectra of [L-AlaOiPr][IBU].

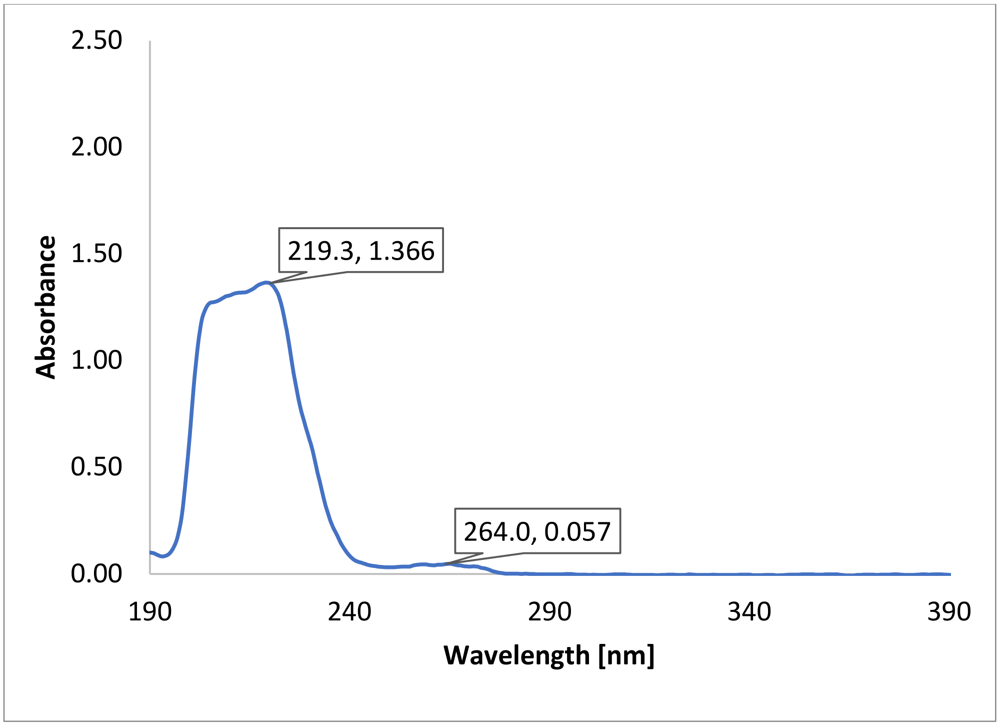

Figure S46. UV-Vis spectra of [L-ValOiPr][IBU].

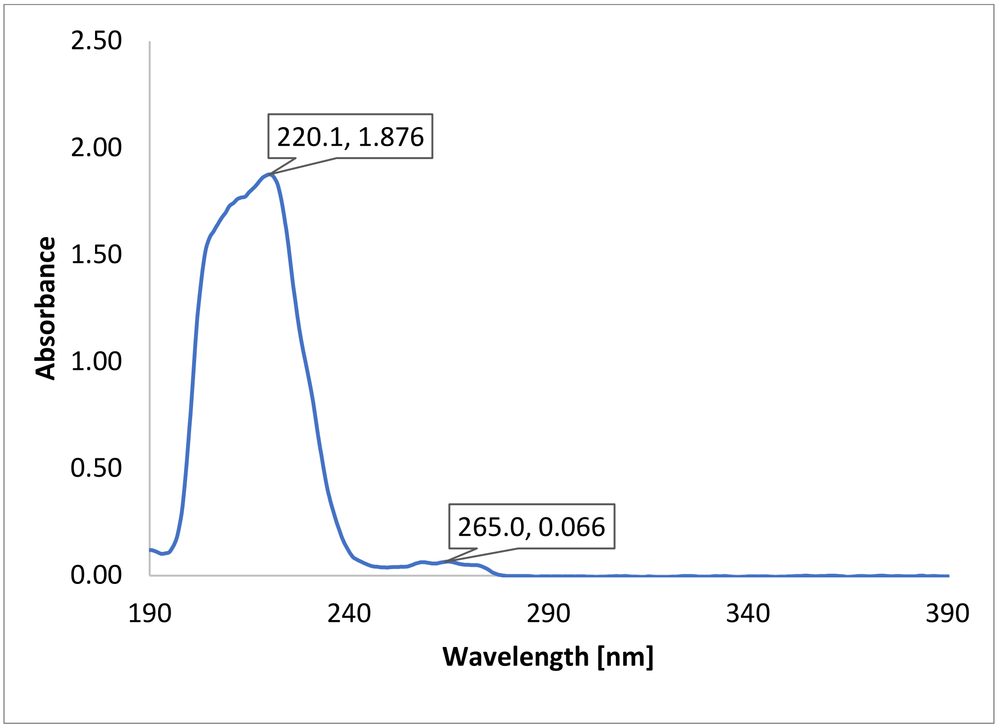

Figure S47. UV-Vis spectra of [L-IleOiPr][IBU].

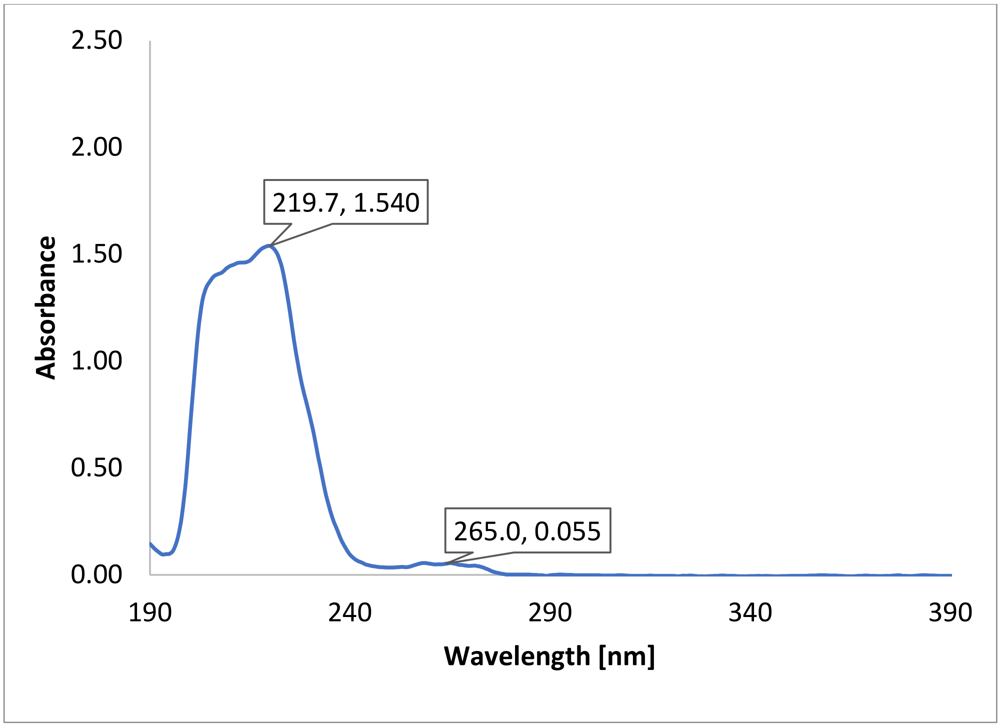

Figure S48. UV-Vis spectra of [L-LeuOiPr][IBU].

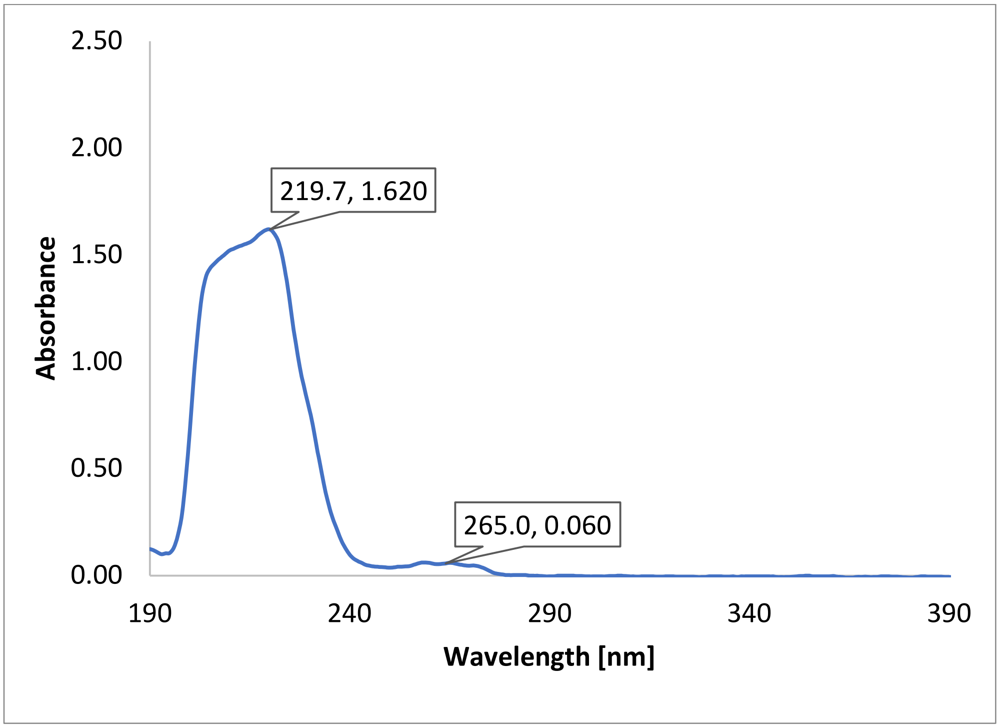

Figure S49. UV-Vis spectra of [L-SerOiPr][IBU].

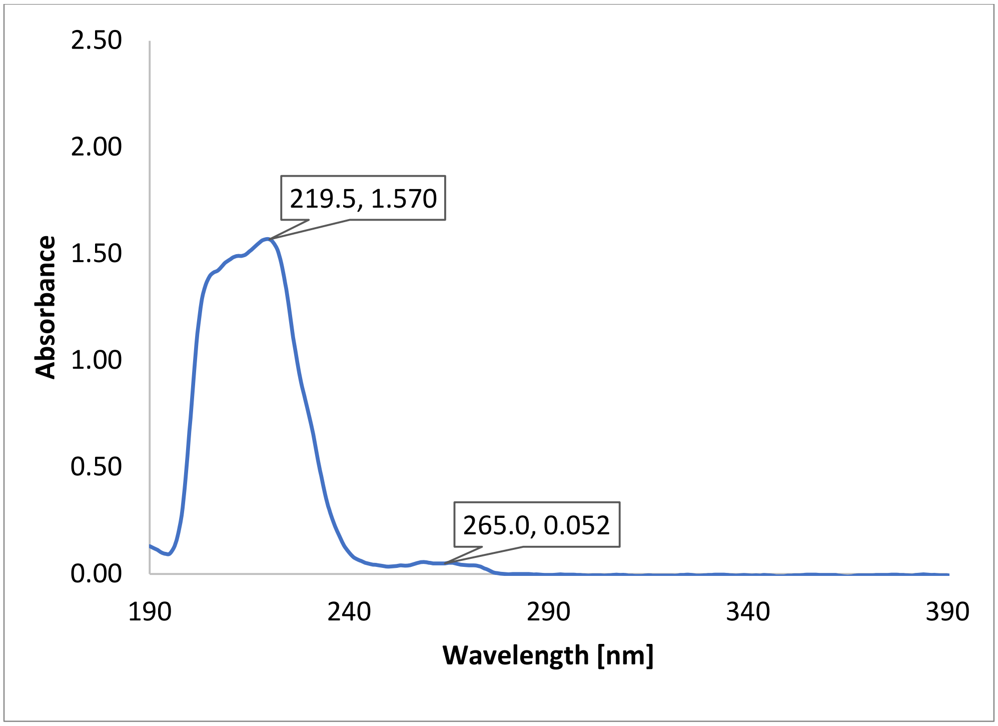

Figure S50. UV-Vis spectra of [L-ThrOiPr][IBU].

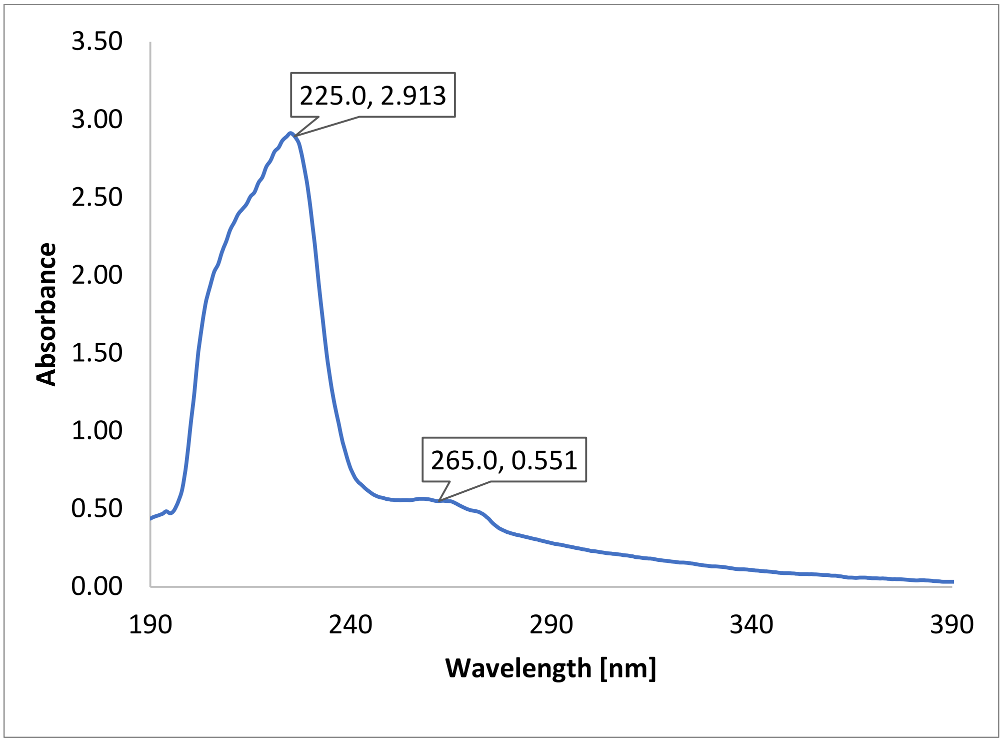

Figure S51. UV-Vis spectra of [L-CysOiPr][IBU].

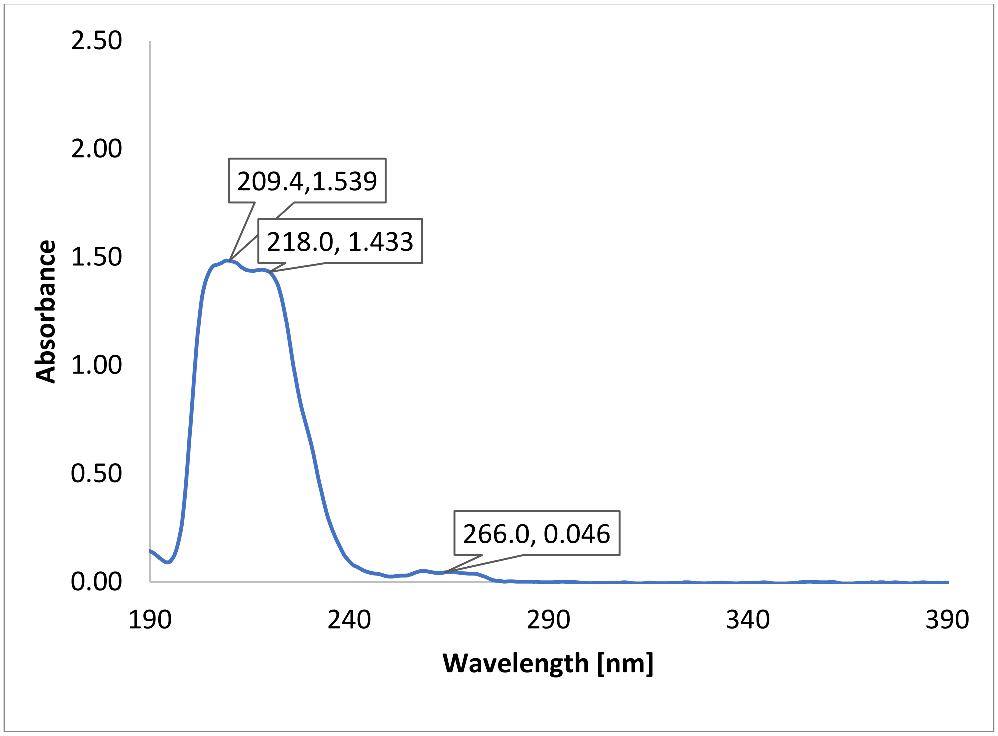

Figure S52. UV-Vis spectra of [L-MetOiPr][IBU].

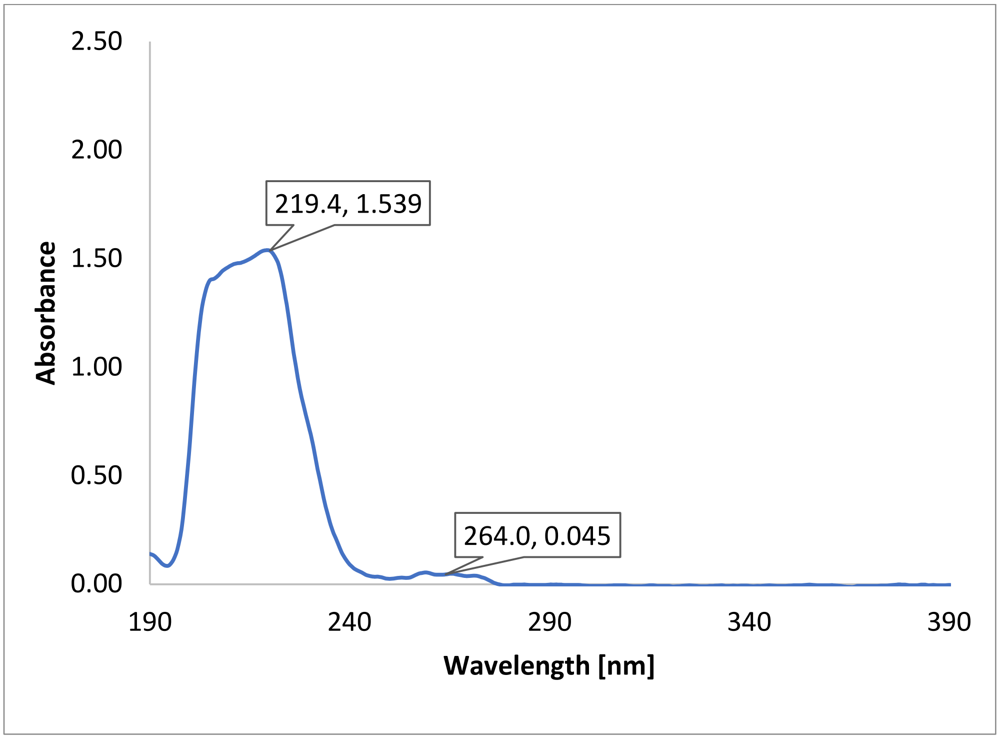

Figure S53. UV-Vis spectra of [L-Asp(OiPr)$_2$][IBU].

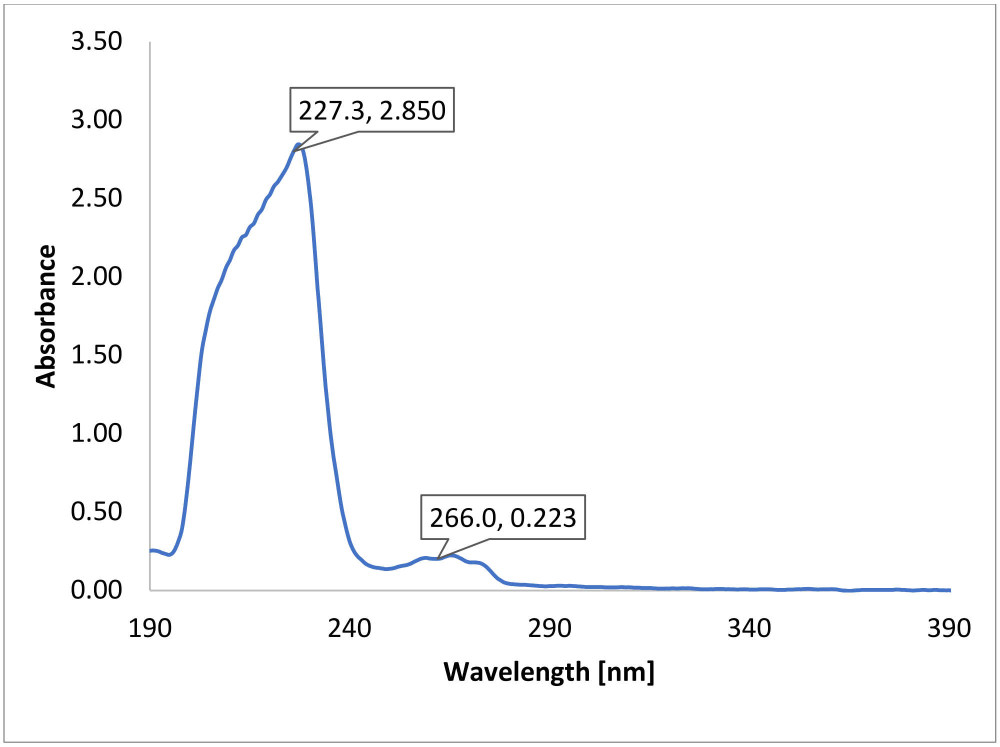

Figure S54. UV-Vis spectra of [L-LysOiPr][IBU].

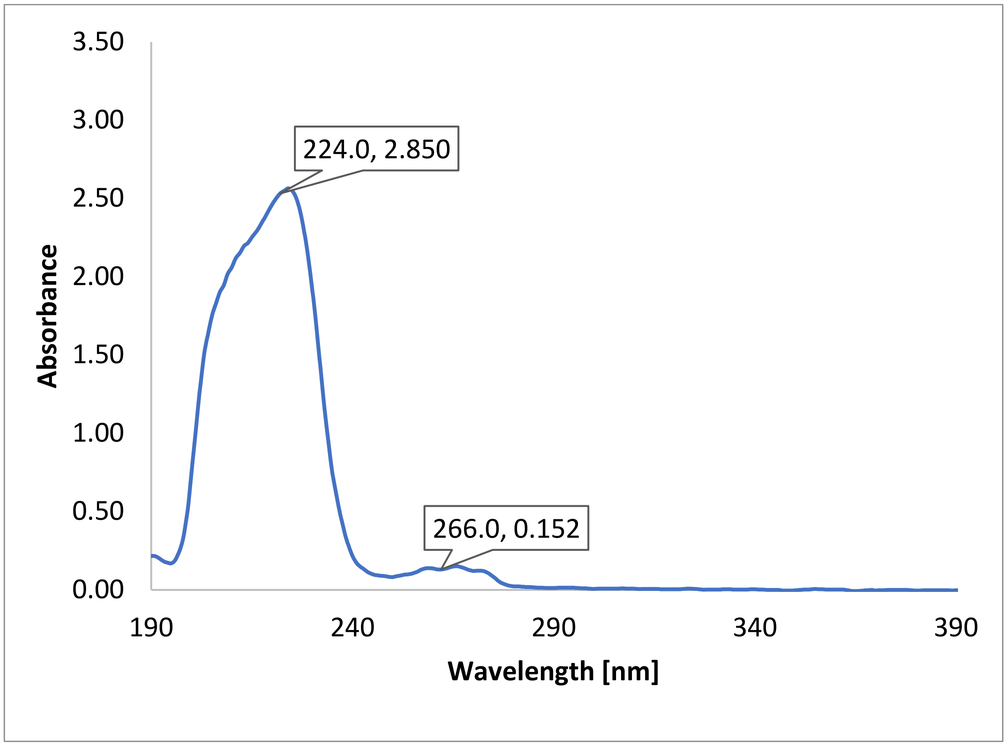

Figure S55. UV-Vis spectra of [L-LysOiPr][IBU]2.

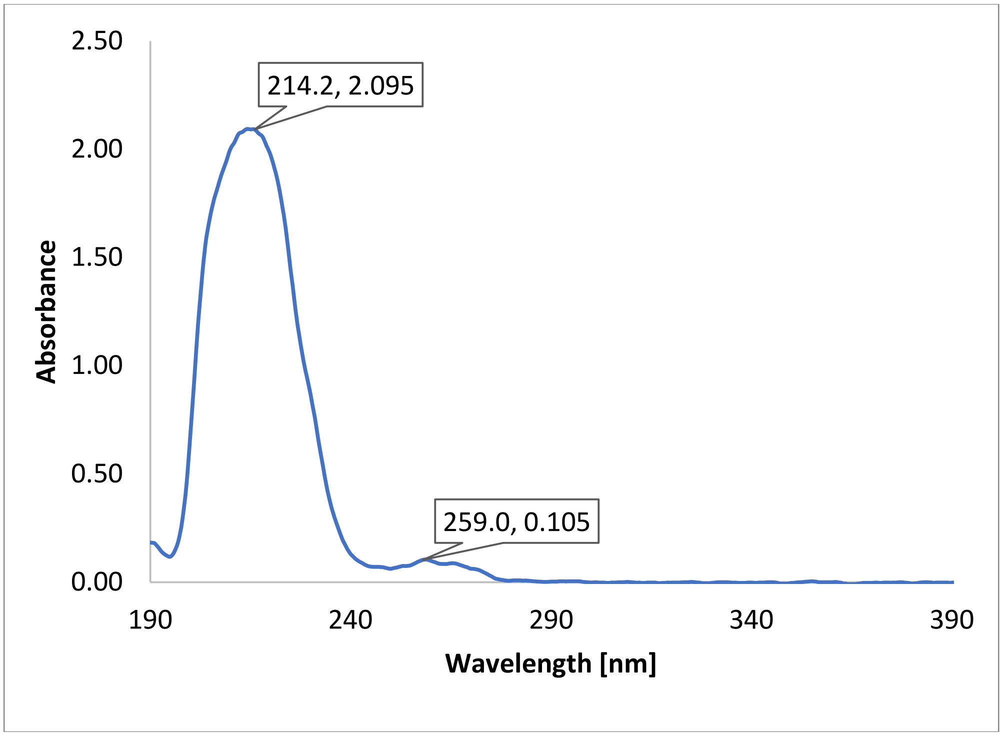

Figure S56. UV-Vis spectra of [L-PheOiPr][IBU].

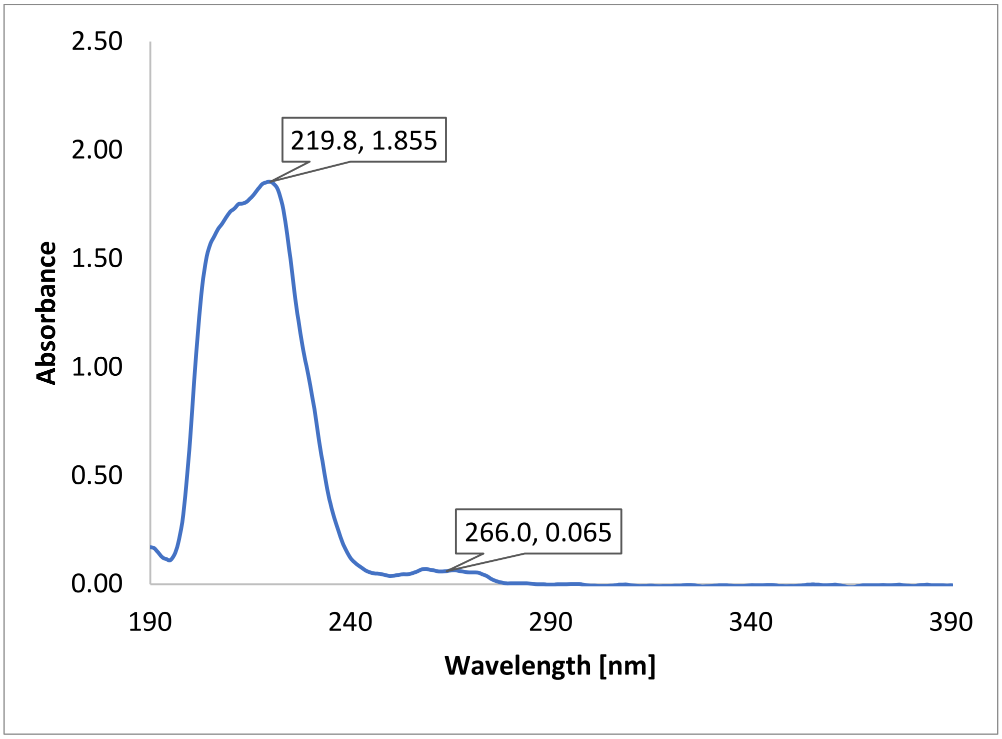

Figure S57. UV-Vis spectra of [L-ProOiPr][IBU].

X-ray diffraction (XRD) patterns of [AAOiPr][IBU]

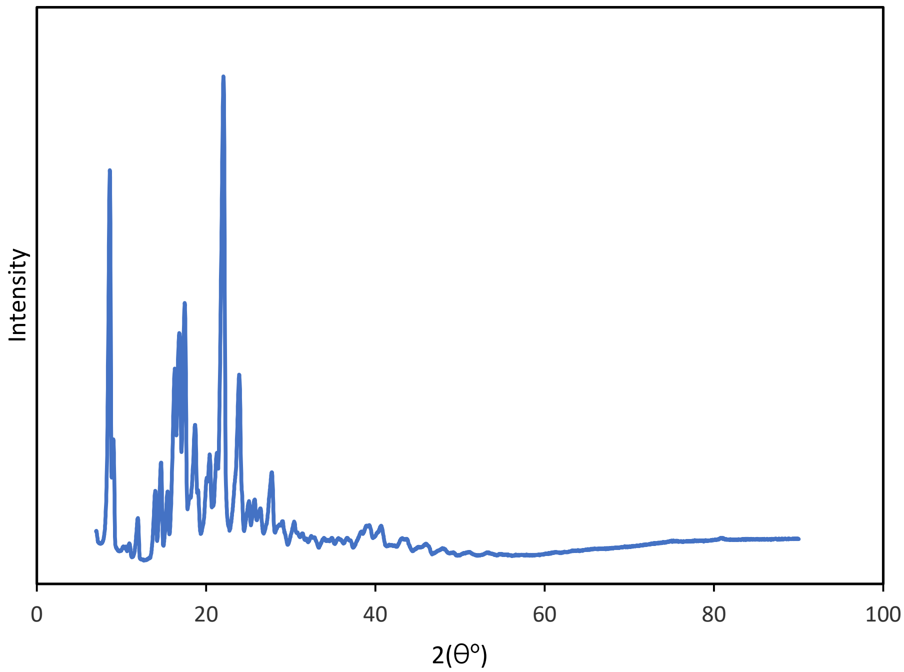

Figure S58. XRD pattern of [GlyOiPr][IBU].

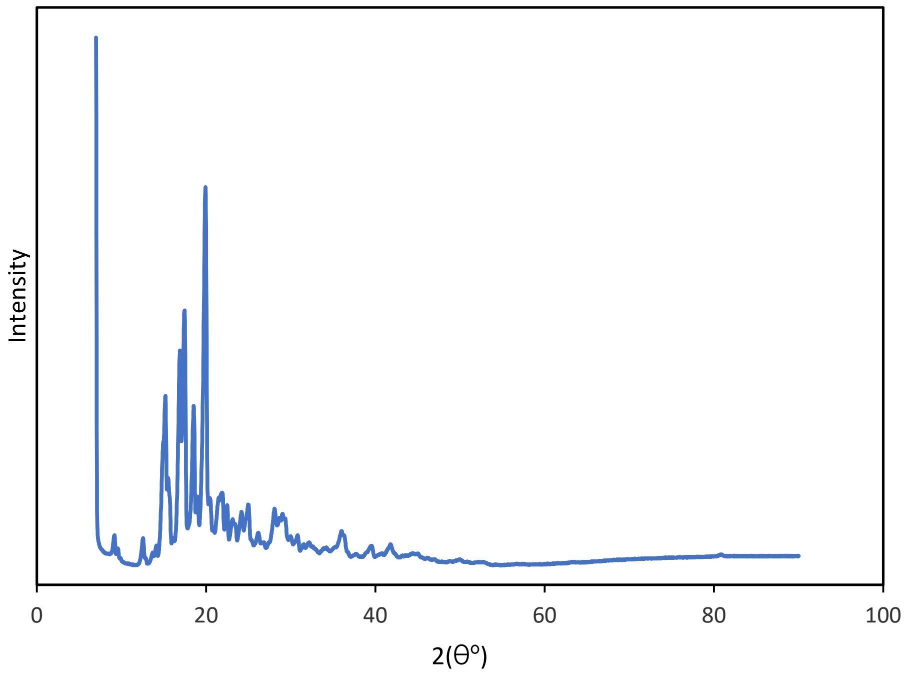

Figure S59. XRD pattern of [L-AlaOiPr][IBU].

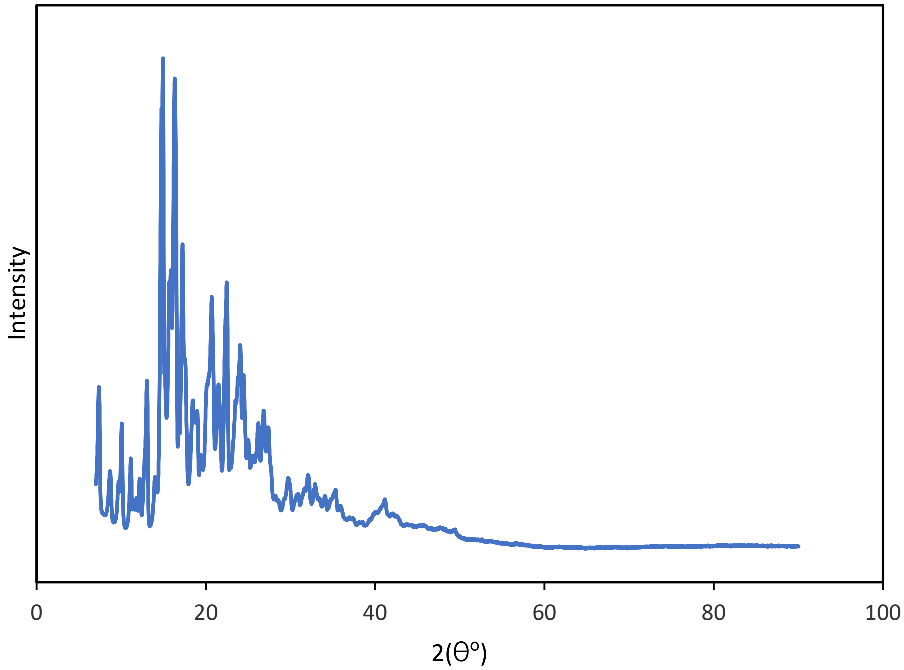

Figure S60. XRD pattern of [L-ValOiPr][IBU].

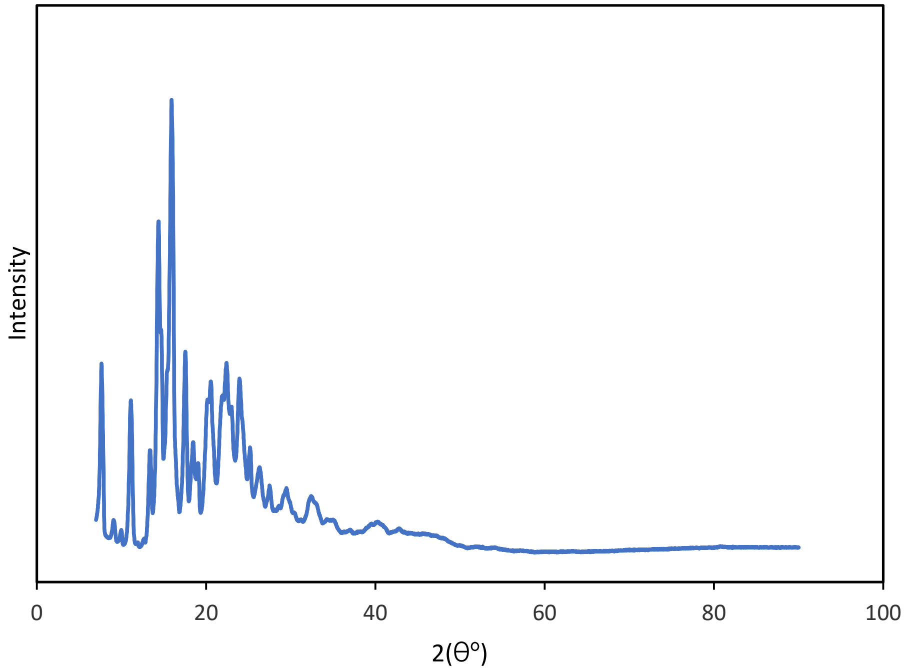

Figure S61. XRD pattern of [L-IleOiPr][IBU].

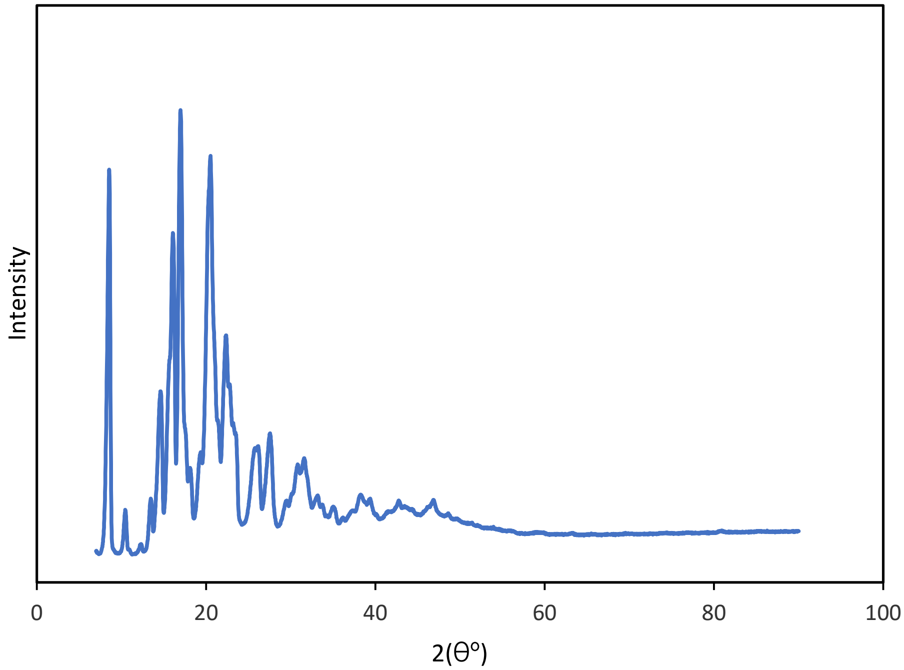

Figure S62. XRD pattern of [L-LeuOiPr][IBU].

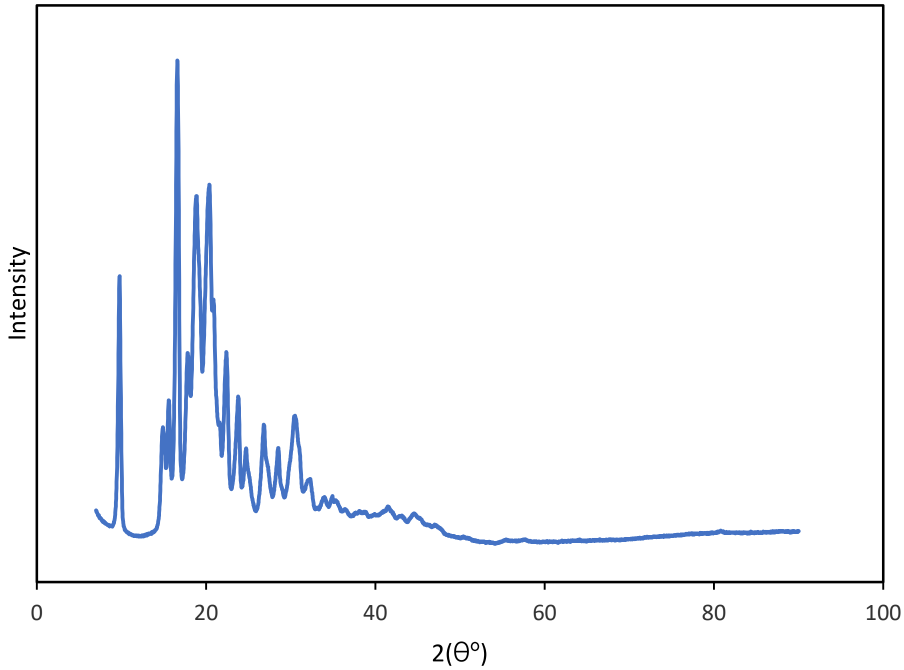

Figure S63. XRD pattern of [L-SerOiPr][IBU].

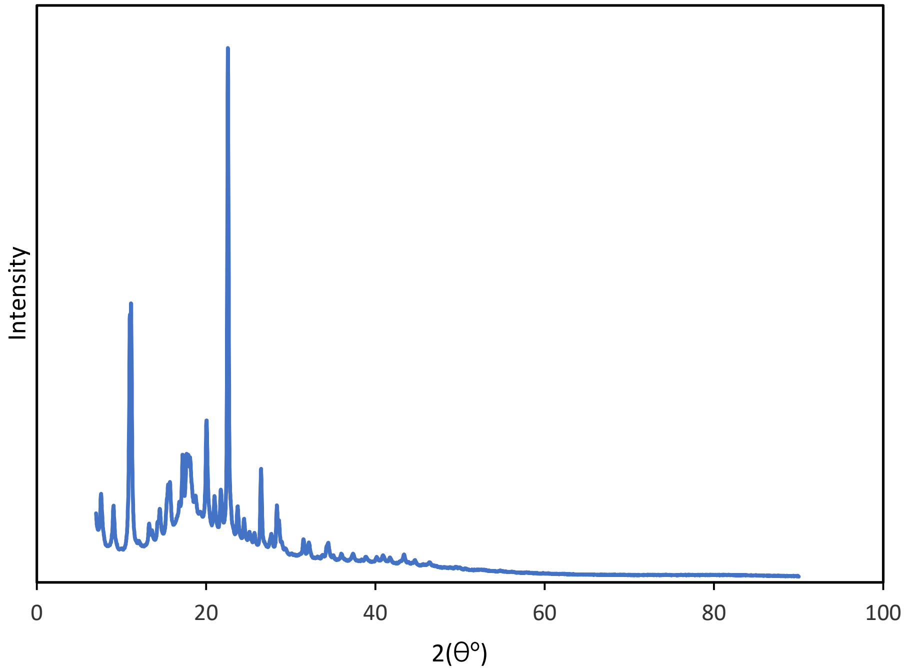

Figure S64. XRD pattern of [L-ThrOiPr][IBU].

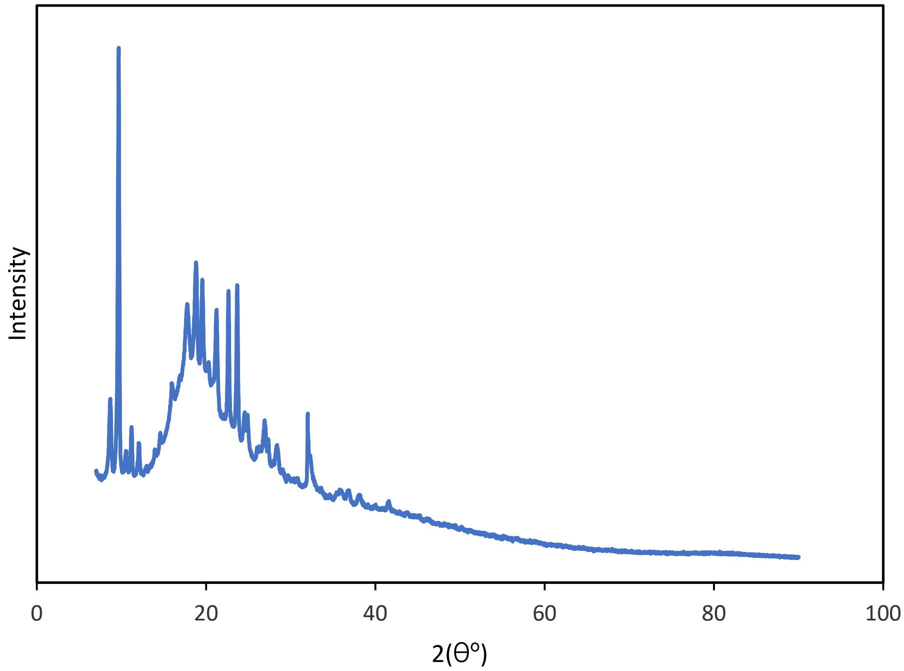

Figure S65. XRD pattern of [L-CysOiPr][IBU].

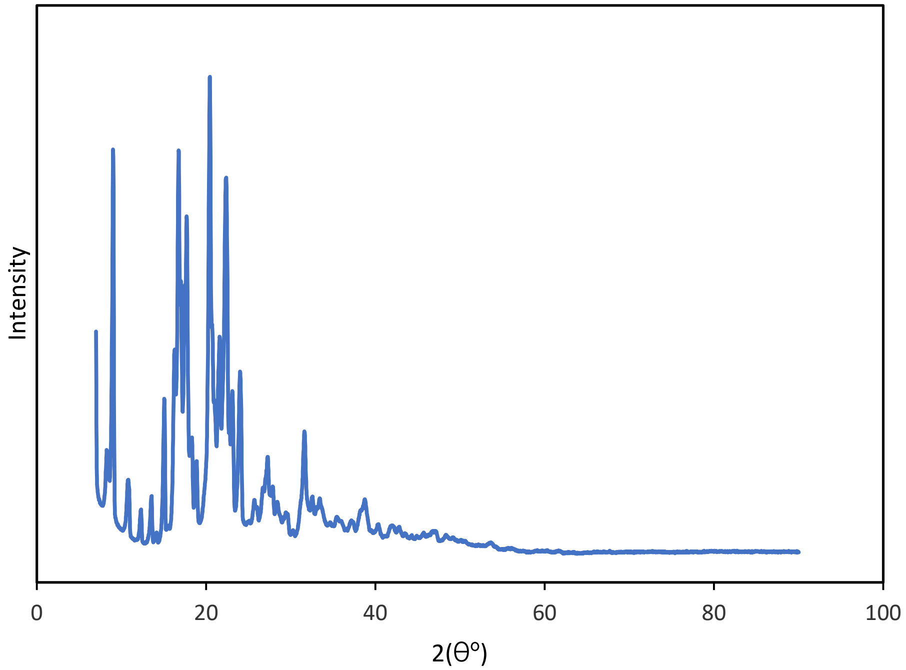

Figure S66. XRD pattern of [L-MetOiPr][IBU].

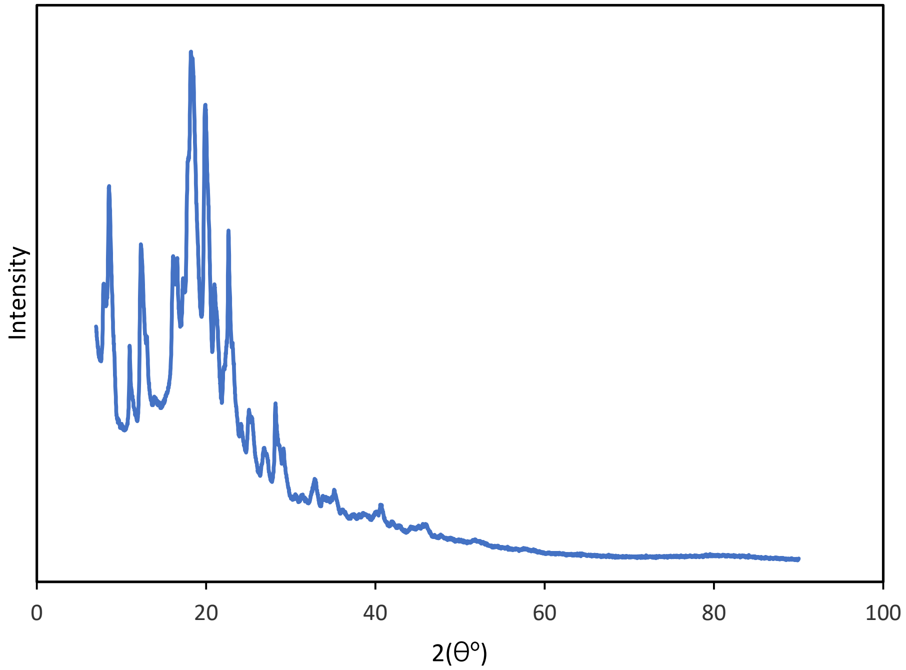

Figure S67. XRD pattern of [L-Asp(OiPr)$_2$][IBU].

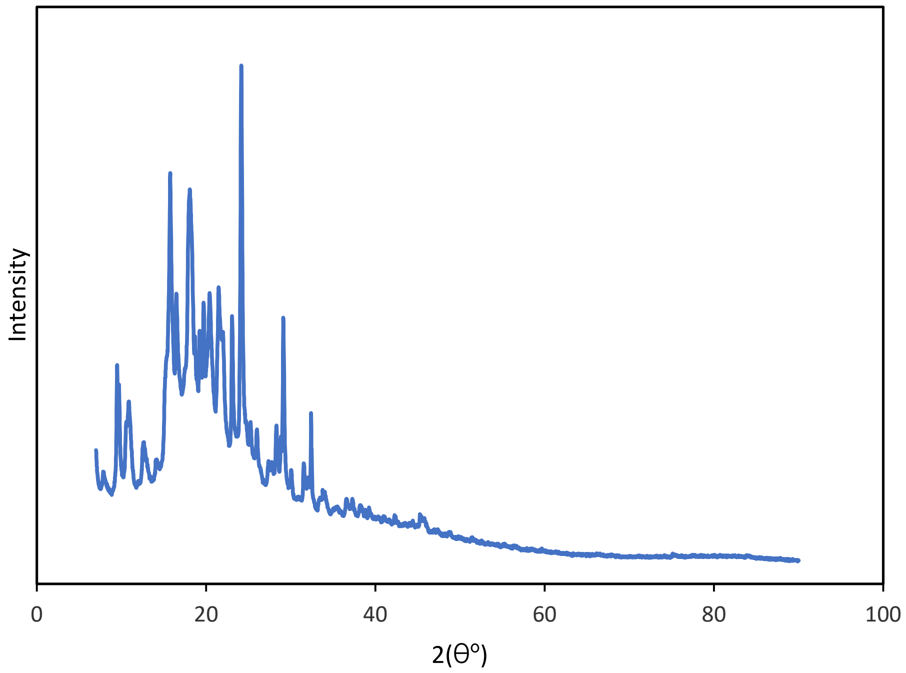

Figure S68. XRD pattern of [L-LysOiPr][IBU].

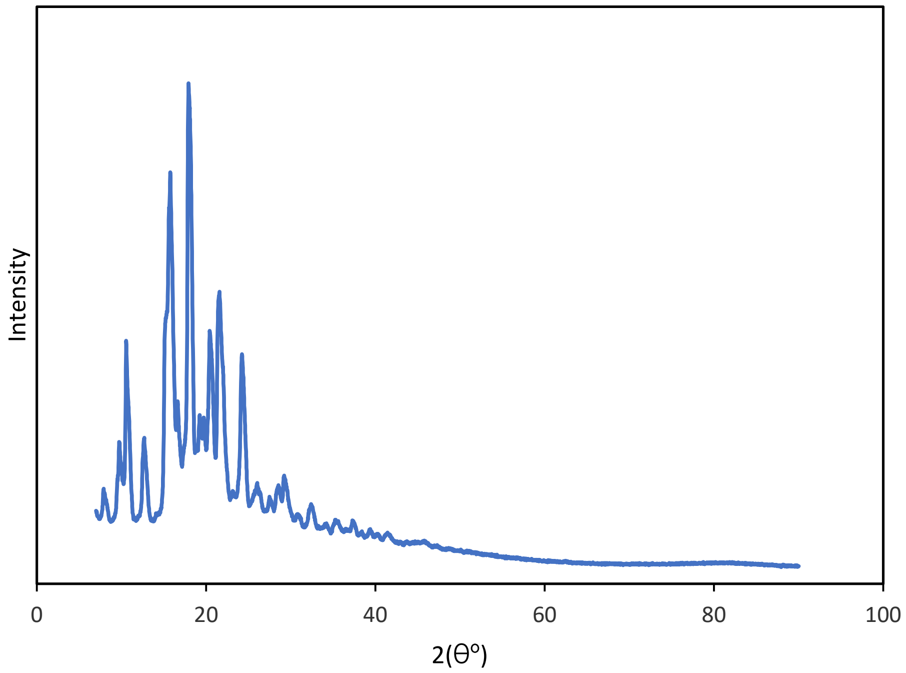

Figure S69. XRD pattern of [L-LysOiPr][IBU]$_2$.

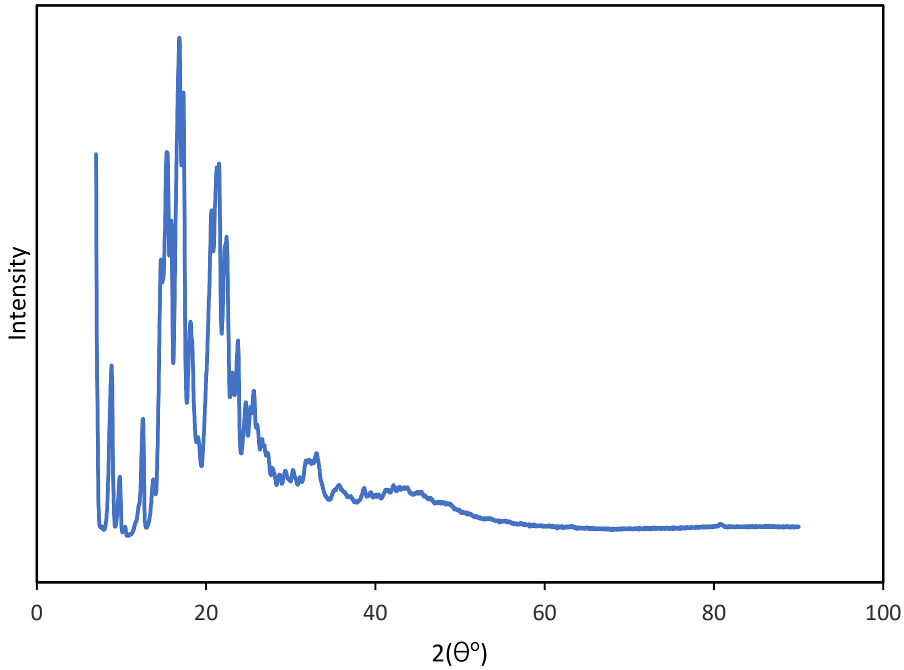

Figure S70. XRD pattern of [L-PheOiPr][IBU].

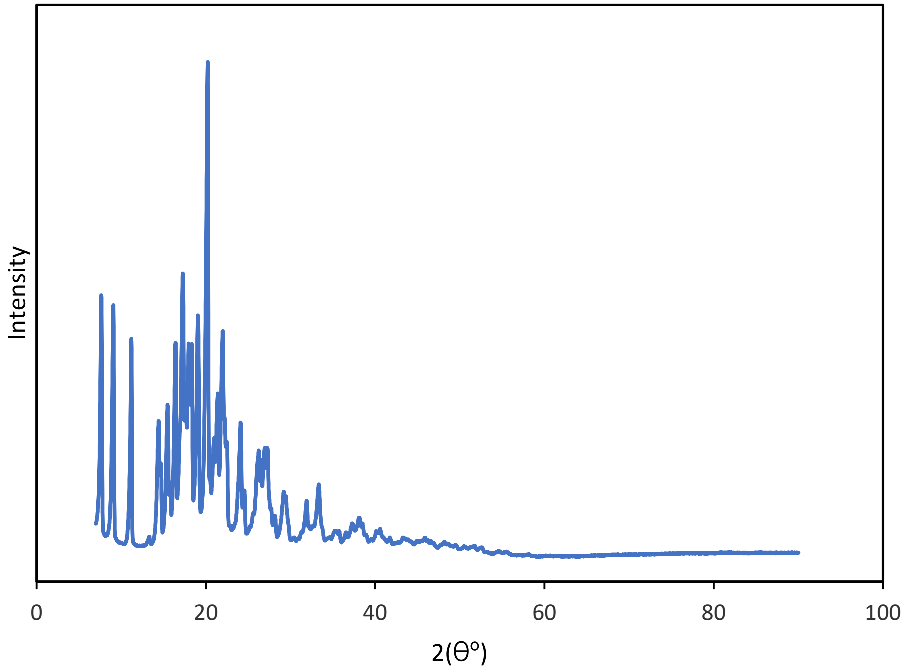

Figure S71. XRD pattern of [L-ProOiPr][IBU].

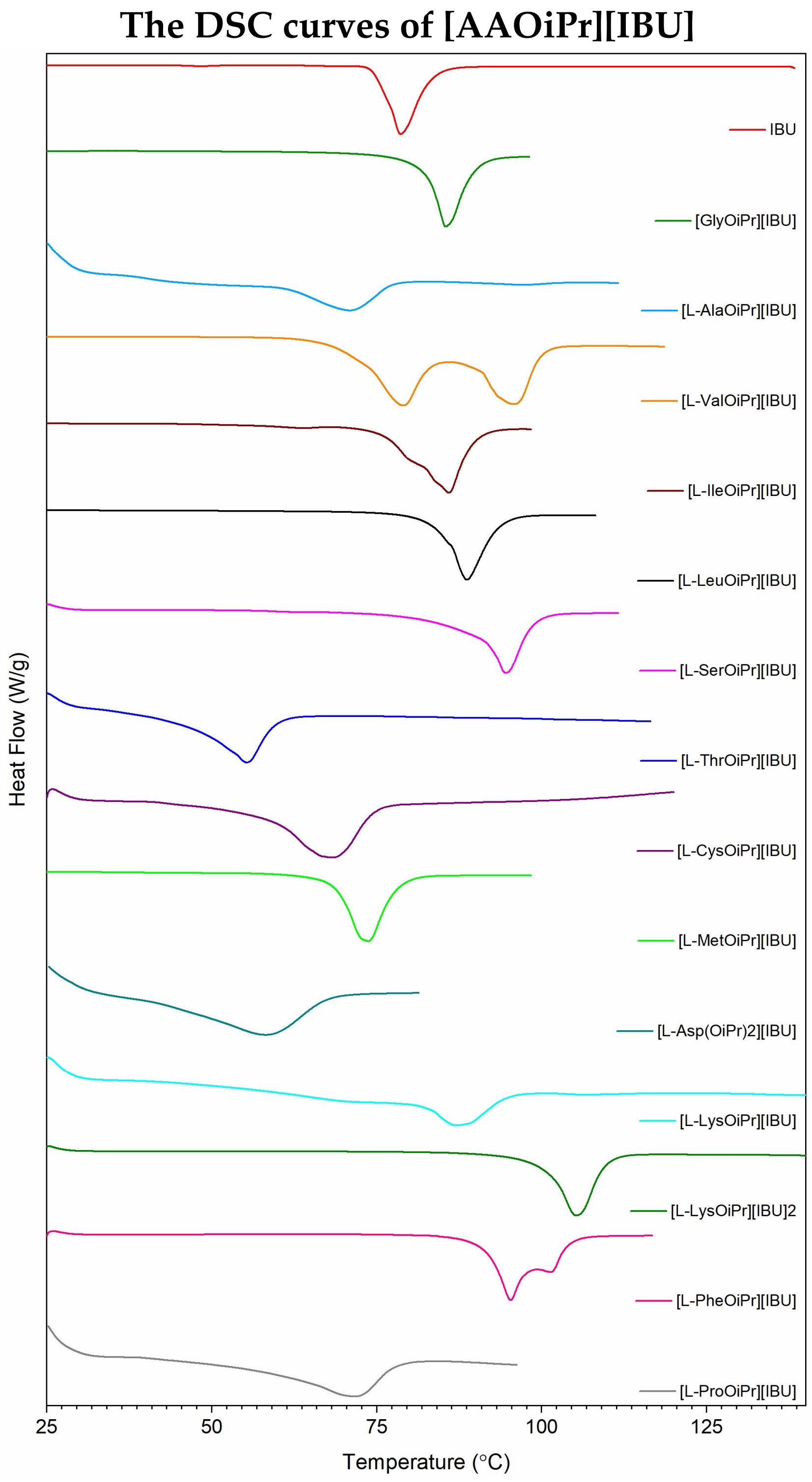

Figure S72. The DSC curves of ibuprofen and ibuprofen salts, from the top: IBU (red), [GlyOiPr][IBU] (green), [L-AlaOiPr][IBU] (blue), [L-ValOiPr][IBU] (orange), [L-IleOiPr][IBU] (maroon), [L-LeuOiPr][IBU] (black), [L-SerOiPr][IBU] (purplish red), [L-ThrOiPr][IBU] (dark blue), [L-CysOiPr][IBU] (purple), [L-MetOiPr][IBU] (yellow-green), [L-Asp(OiPr)$_2$][IBU] (cyan-green), [L-LysOiPr][IBU] (cyan), [L-LysOiPr][IBU]$_2$ (yellowish-green) [L-PheOiPr][IBU] (pink), [L-ProOiPr][IBU] (gray).

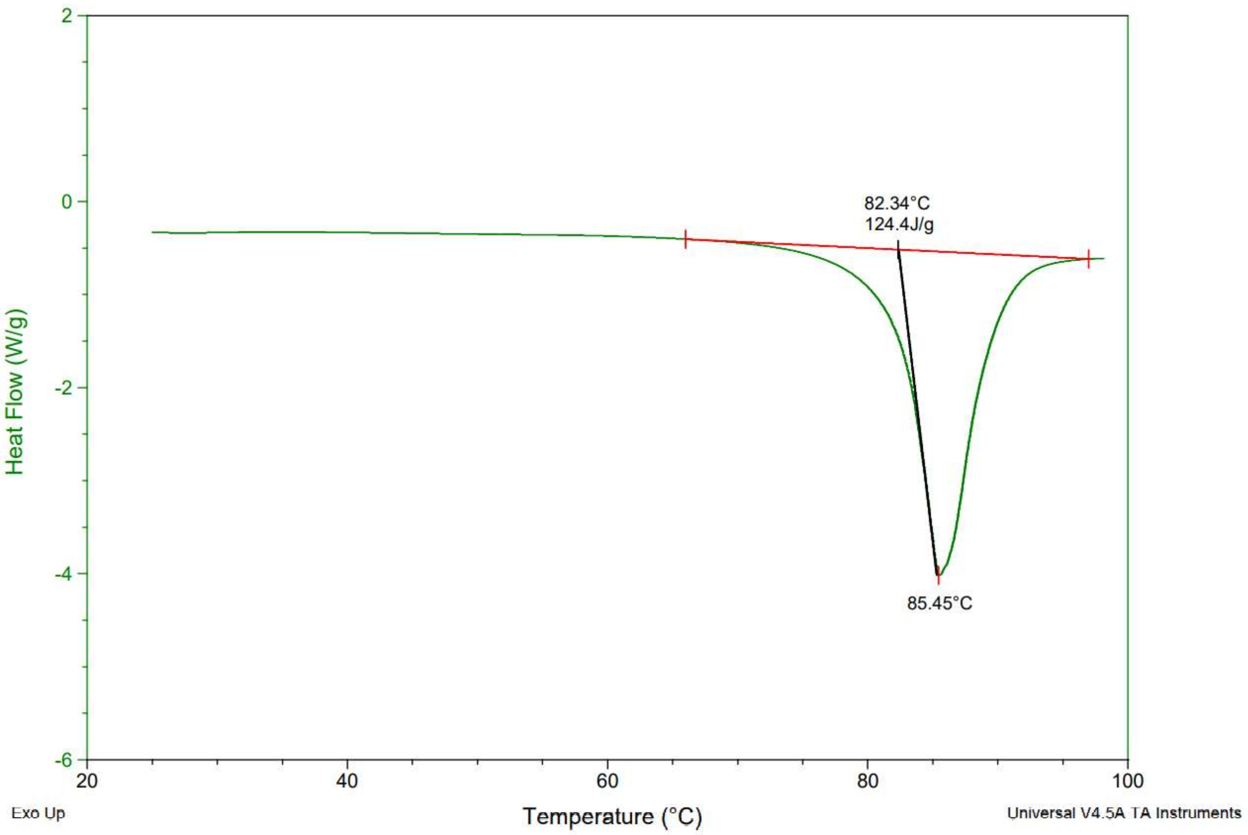

Figure S73. The DSC curves of [GlyOiPr][IBU].

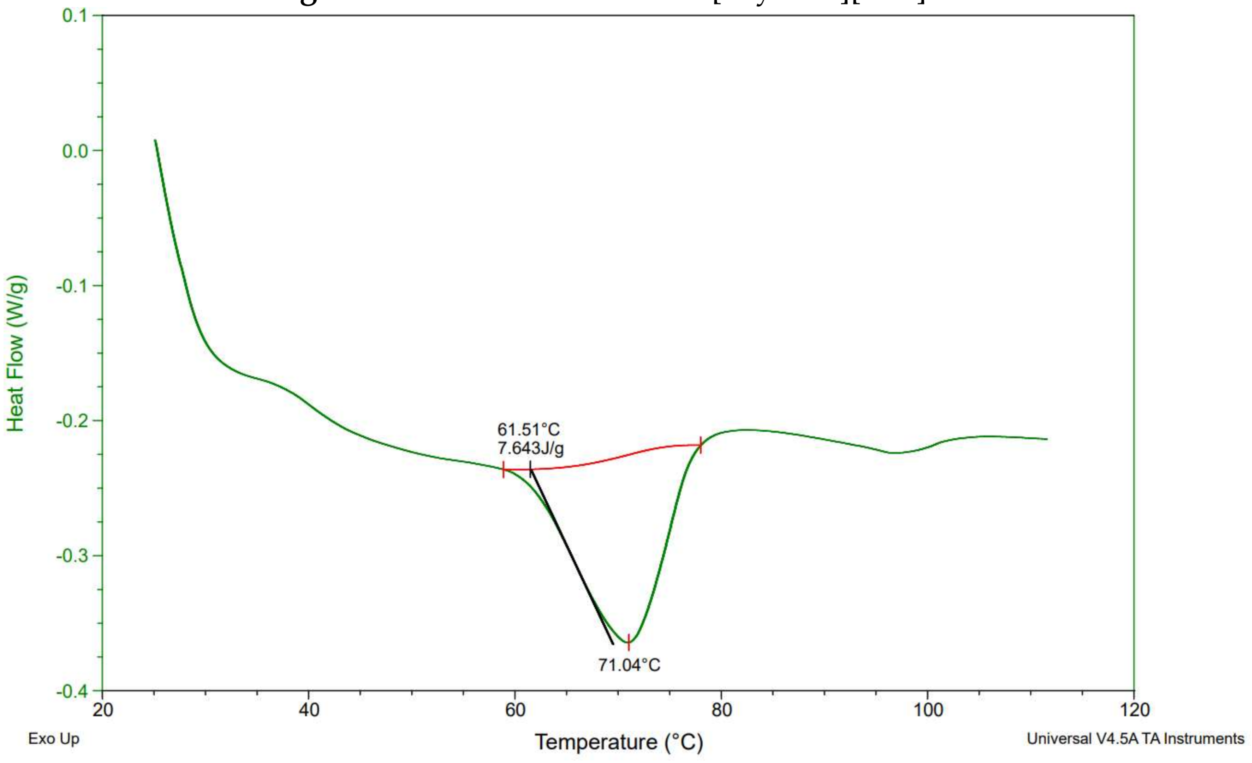

Figure S74. The DSC curves of [L-AlaOiPr][IBU].

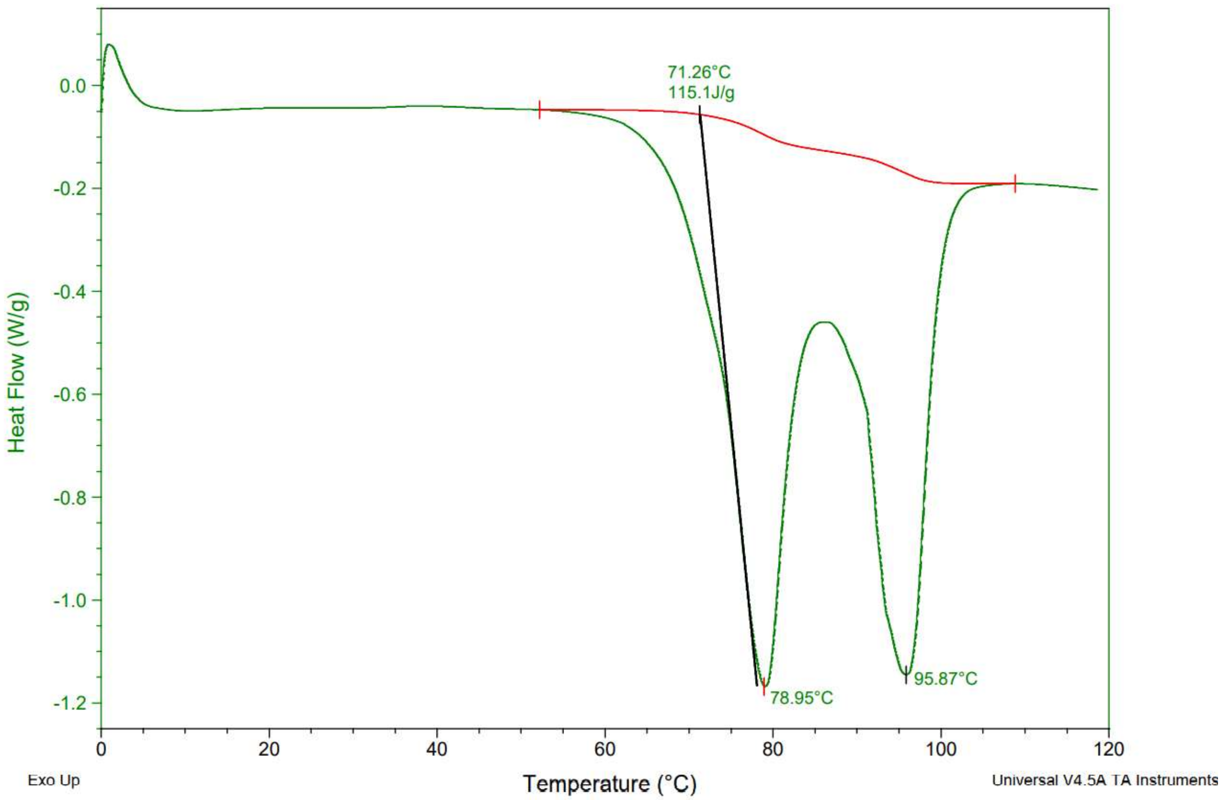

Figure S75. The DSC curves of [L-ValOiPr][IBU].

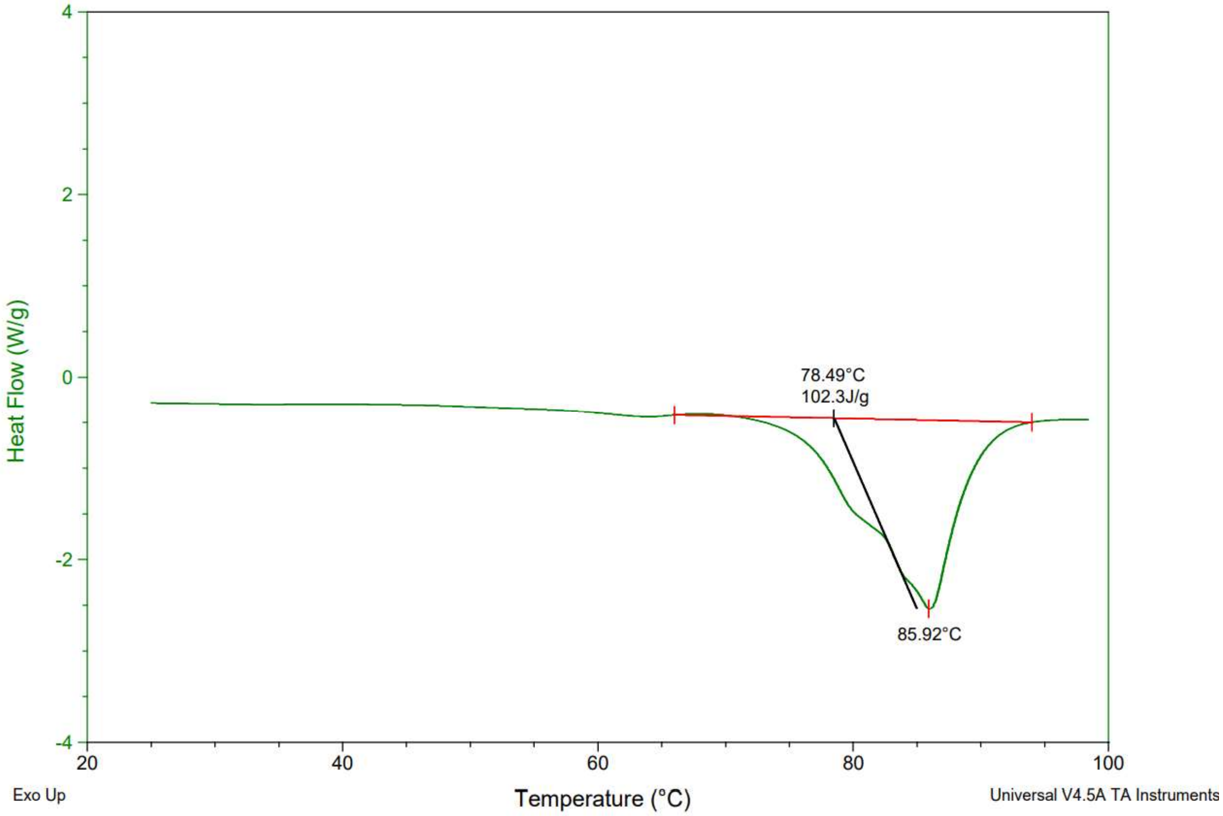

Figure S76. The DSC curves of [L-IleOiPr][IBU].

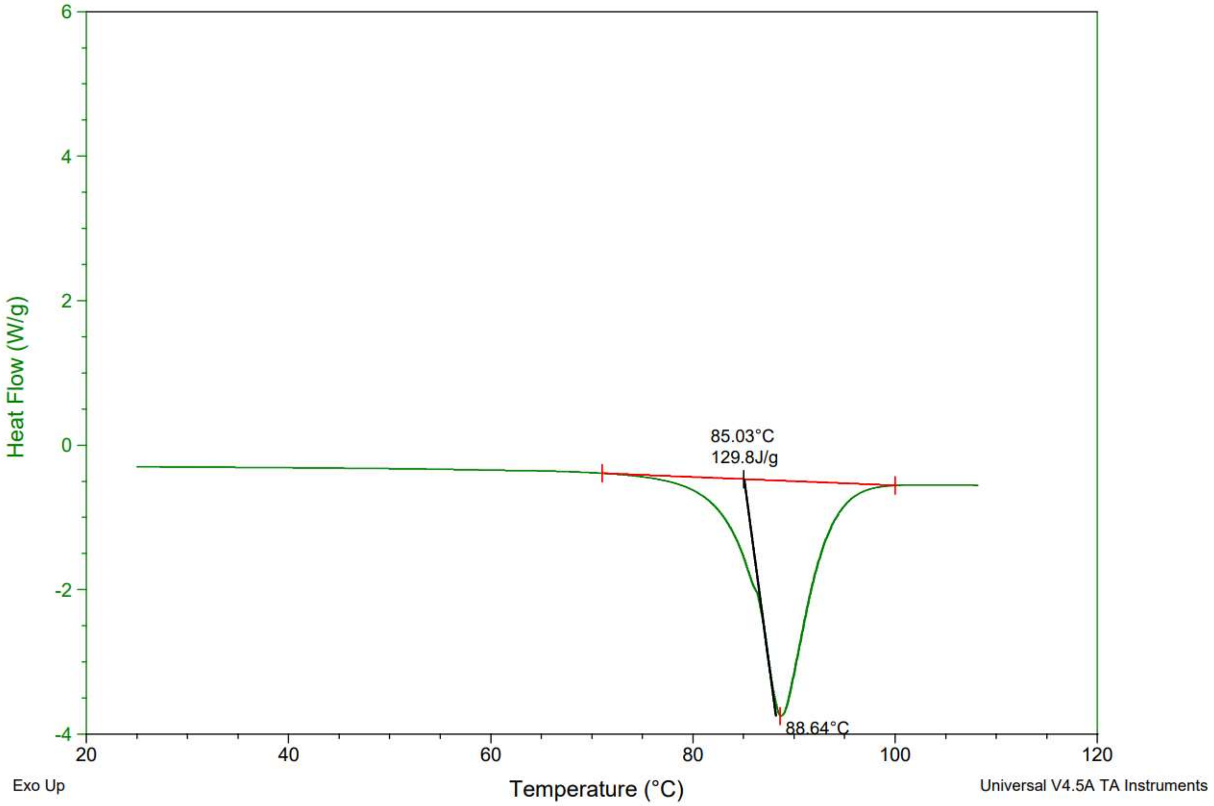

Figure S77. The DSC curves of [L-LeuOiPr][IBU].

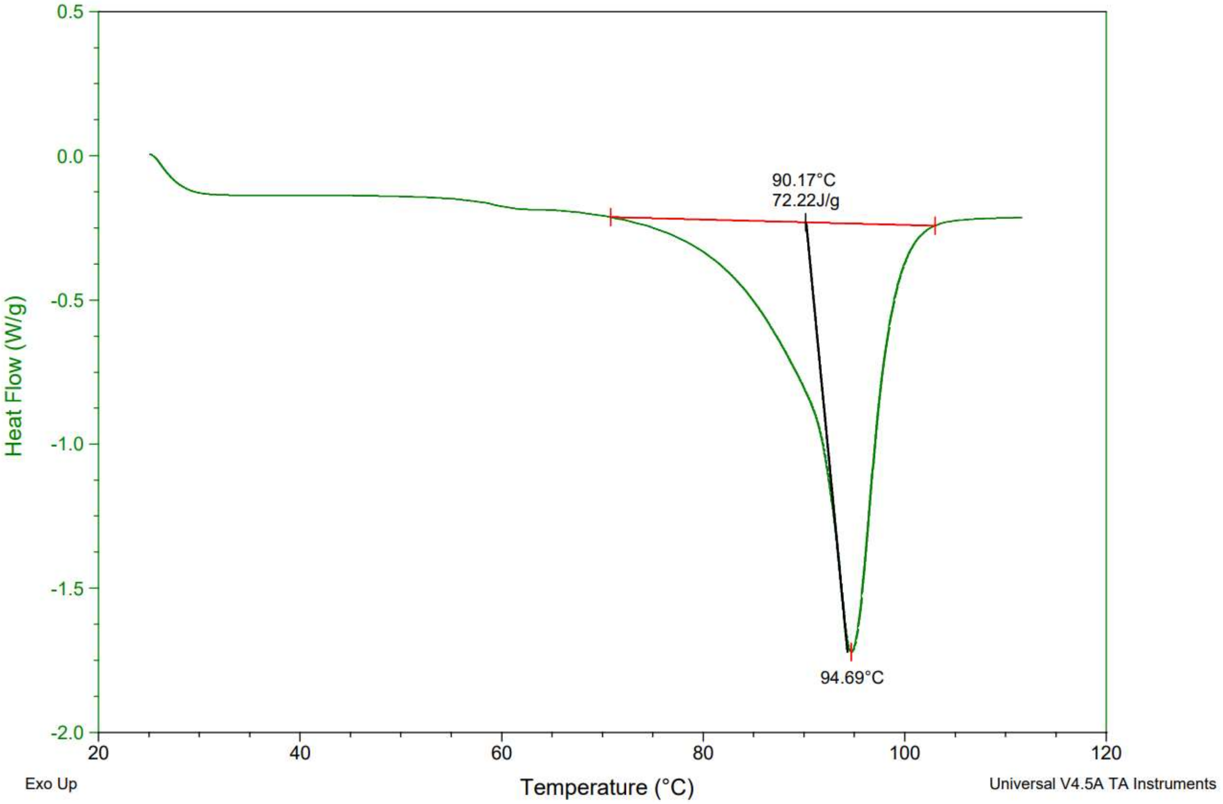

Figure S78. The DSC curves of [L-SerOiPr][IBU].

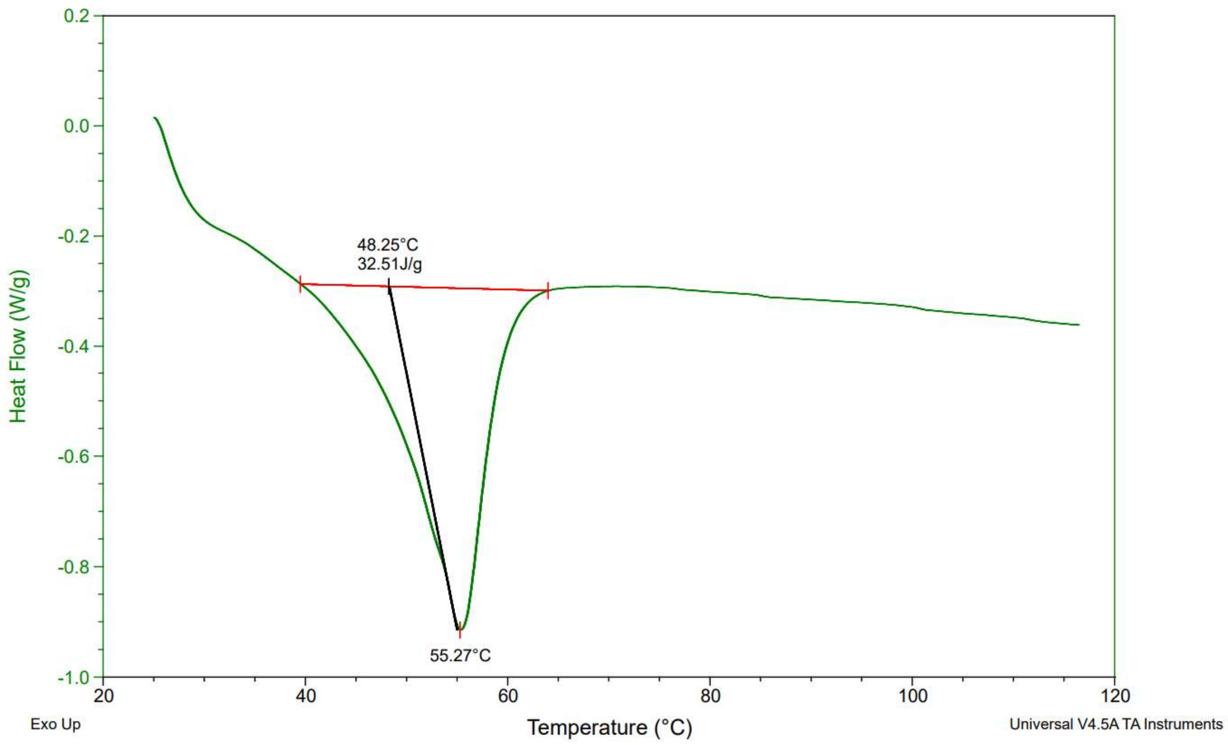

Figure S79. The DSC curves of [L-ThrOiPr][IBU].

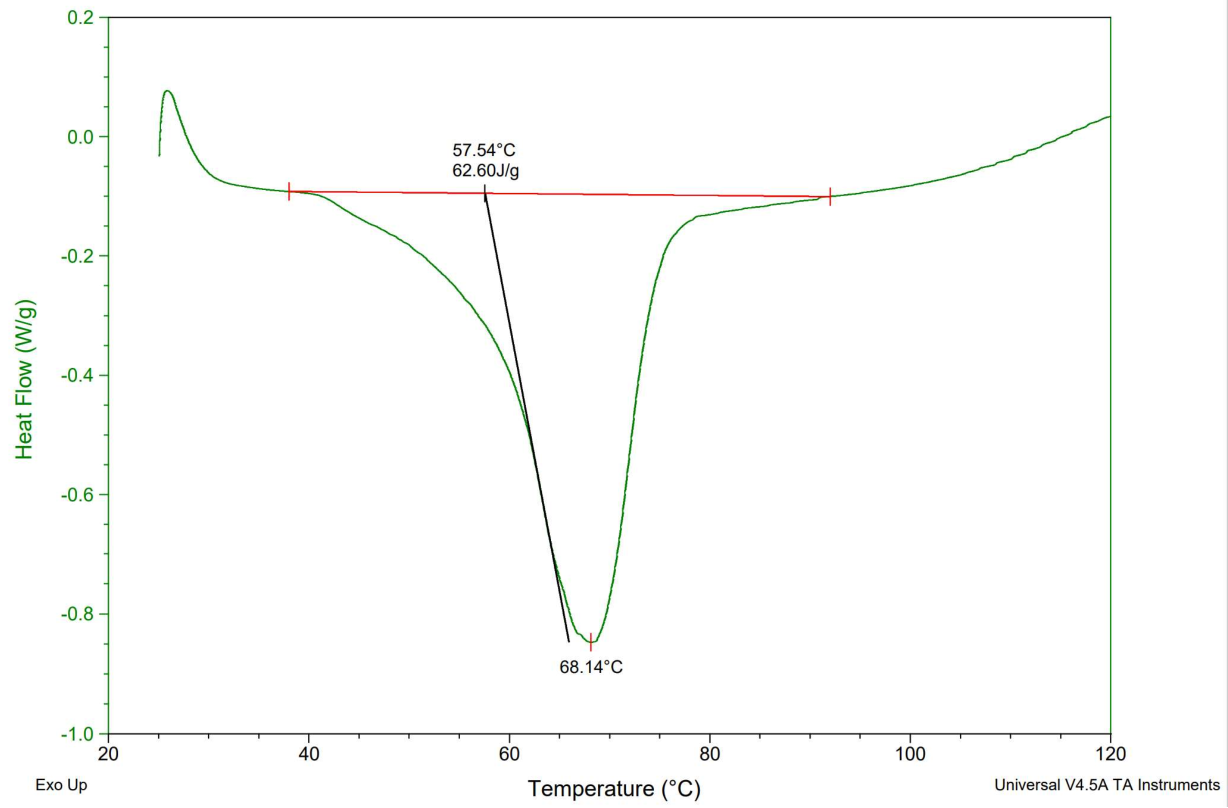

Figure S80. The DSC curves of [L-CysOiPr][IBU].

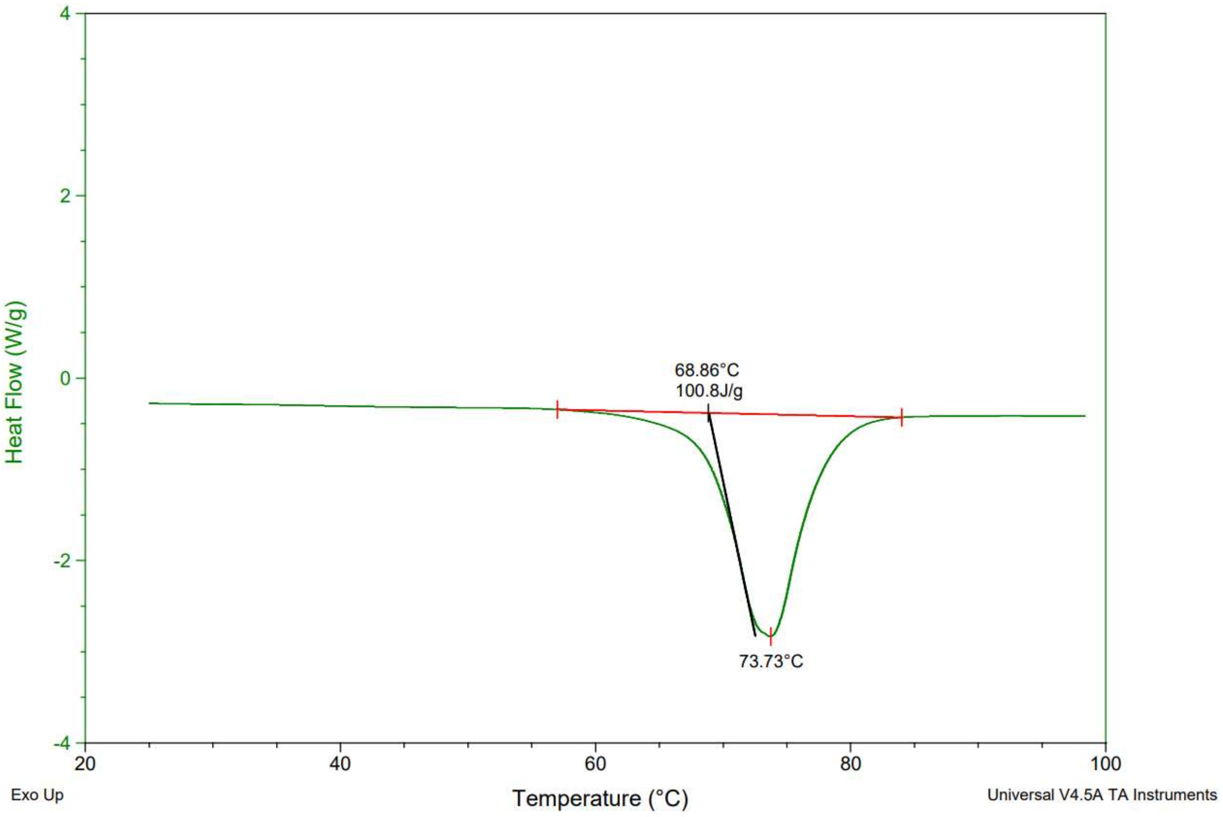

Figure S81. The DSC curves of [L-MetOiPr][IBU].

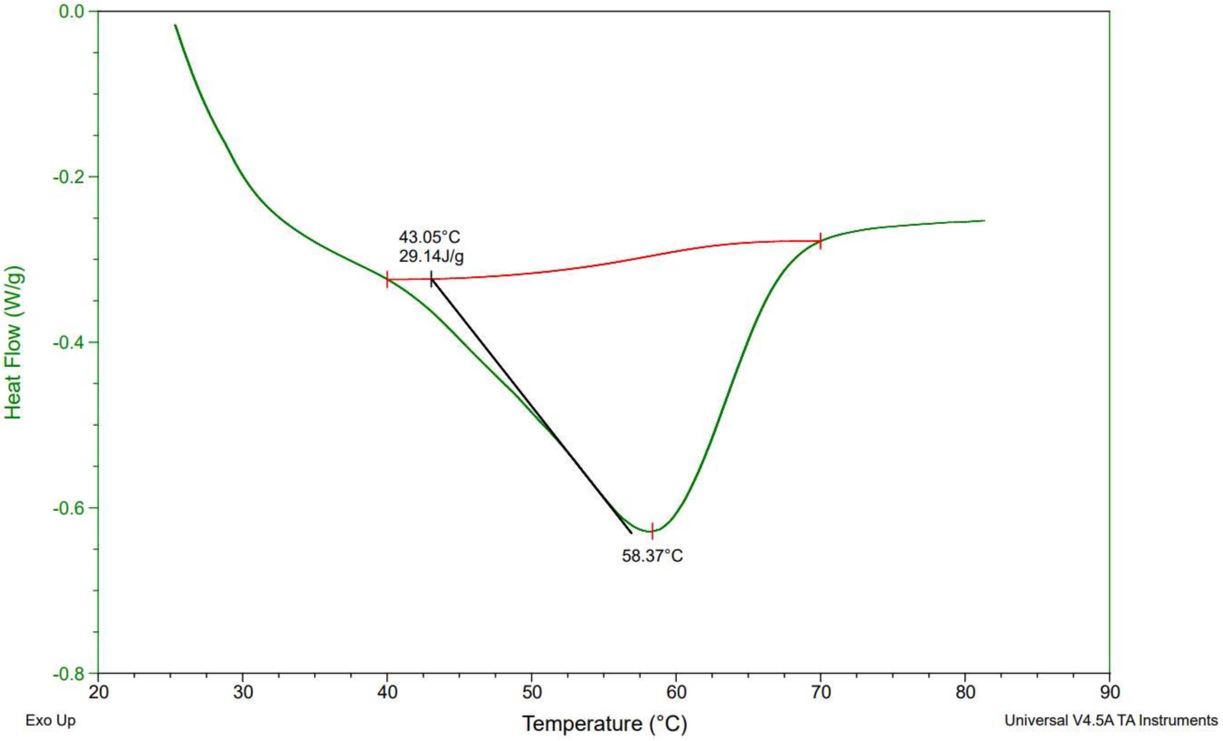

Figure S82. The DSC curves of [L-Asp(OiPr)$_2$][IBU].

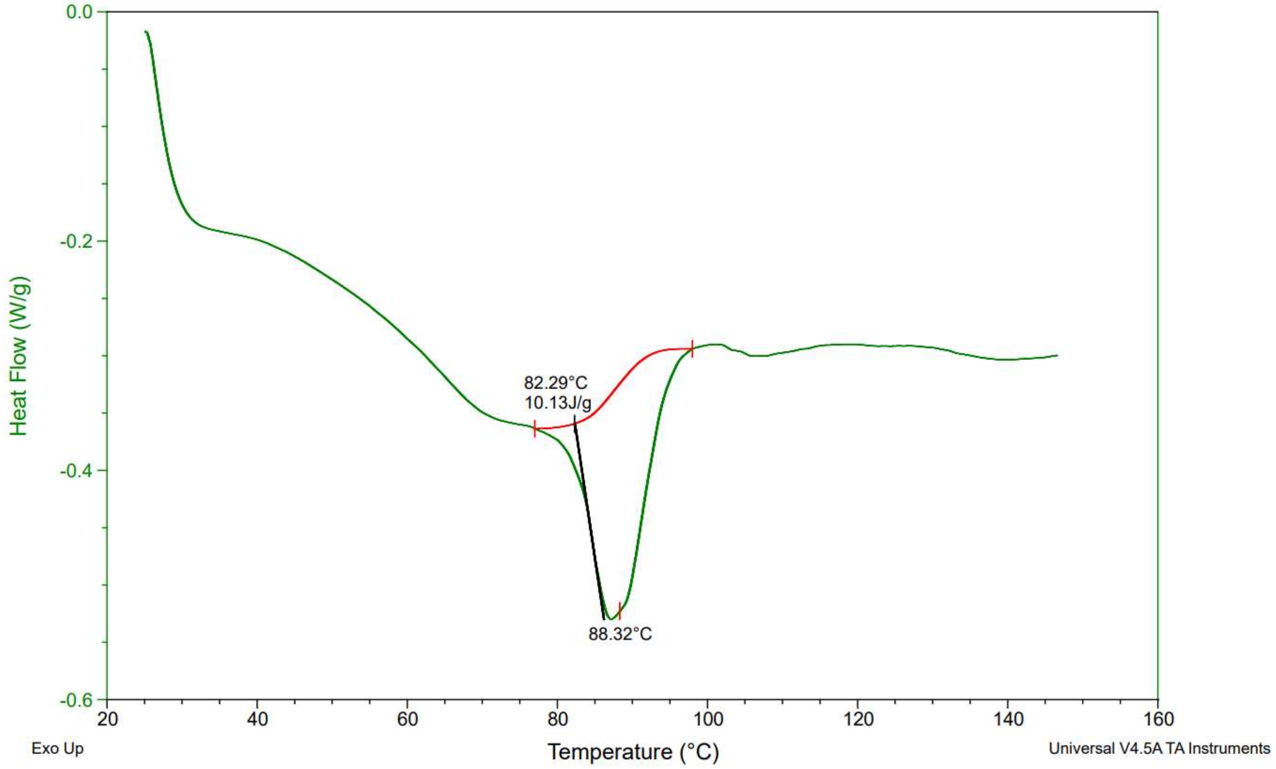

Figure S83. The DSC curves of [L-LysOiPr][IBU].

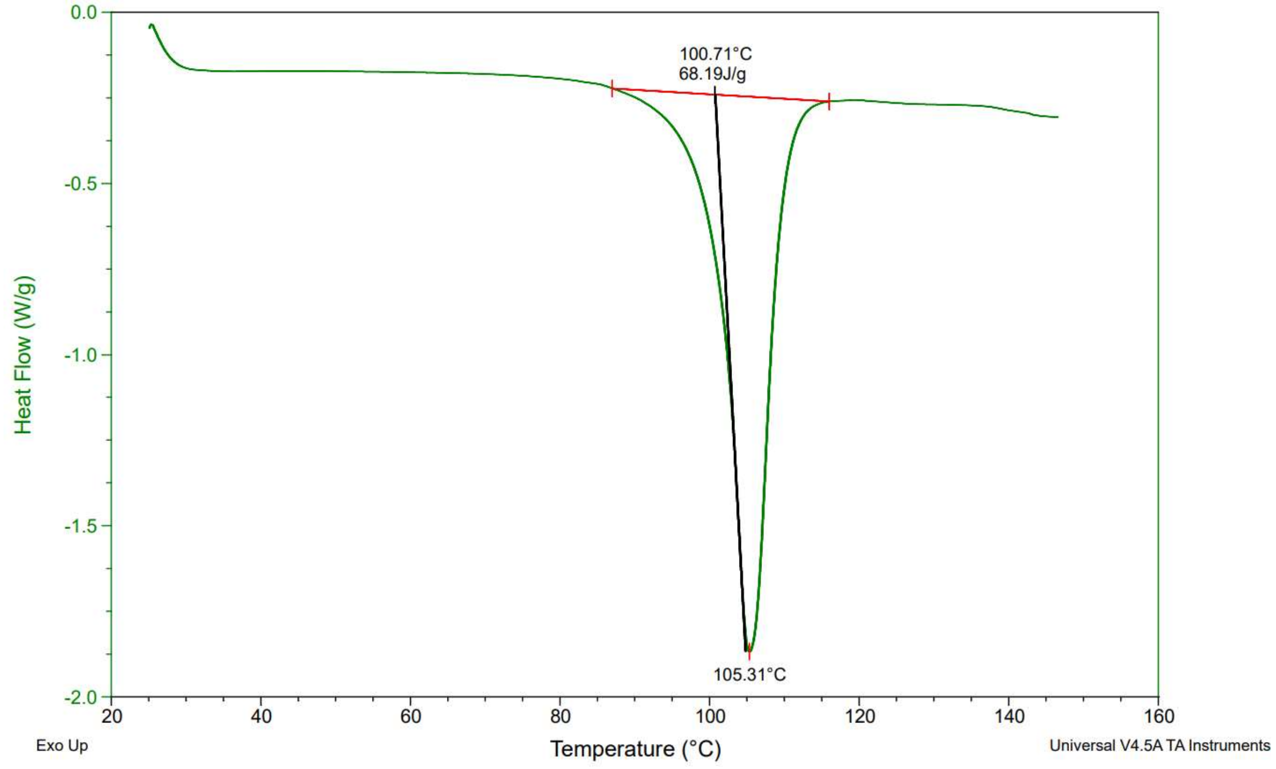

Figure S84. The DSC curves of [L-LysOiPr][IBU]$_2$.

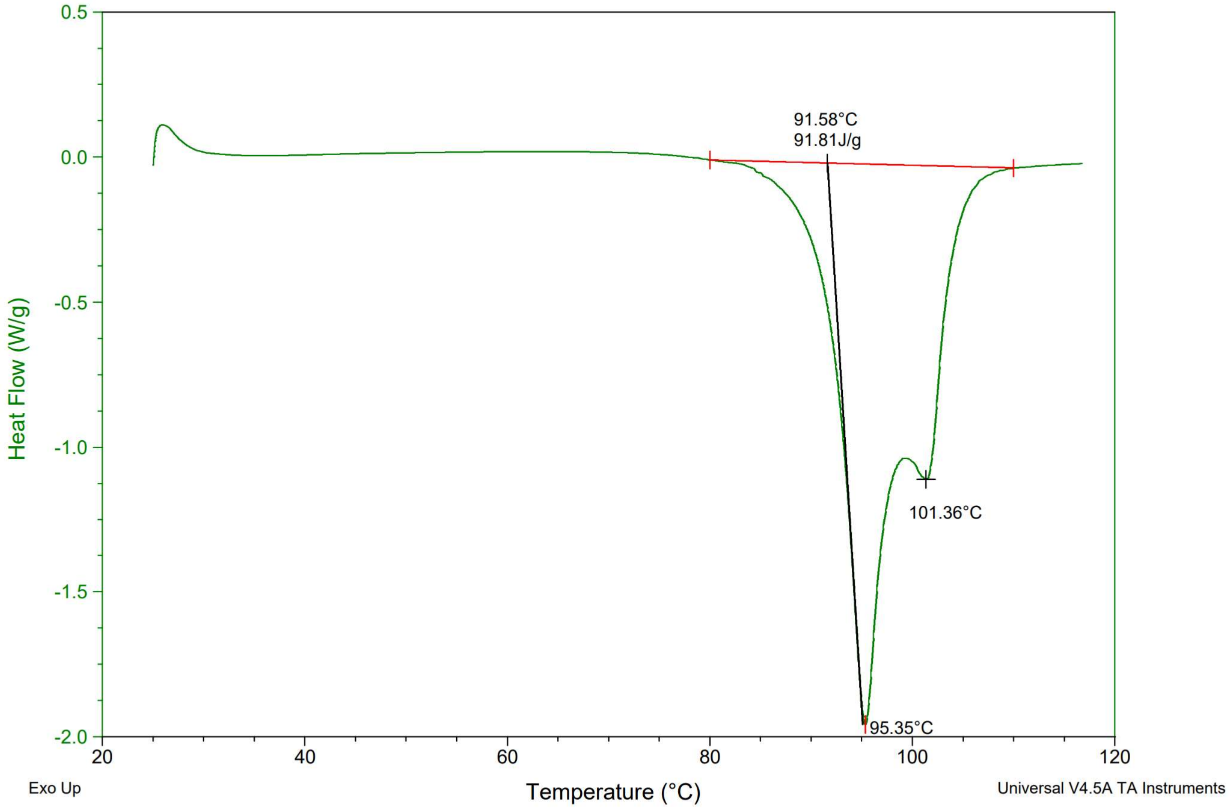

Figure S85. The DSC curves of [L-PheOiPr][IBU].

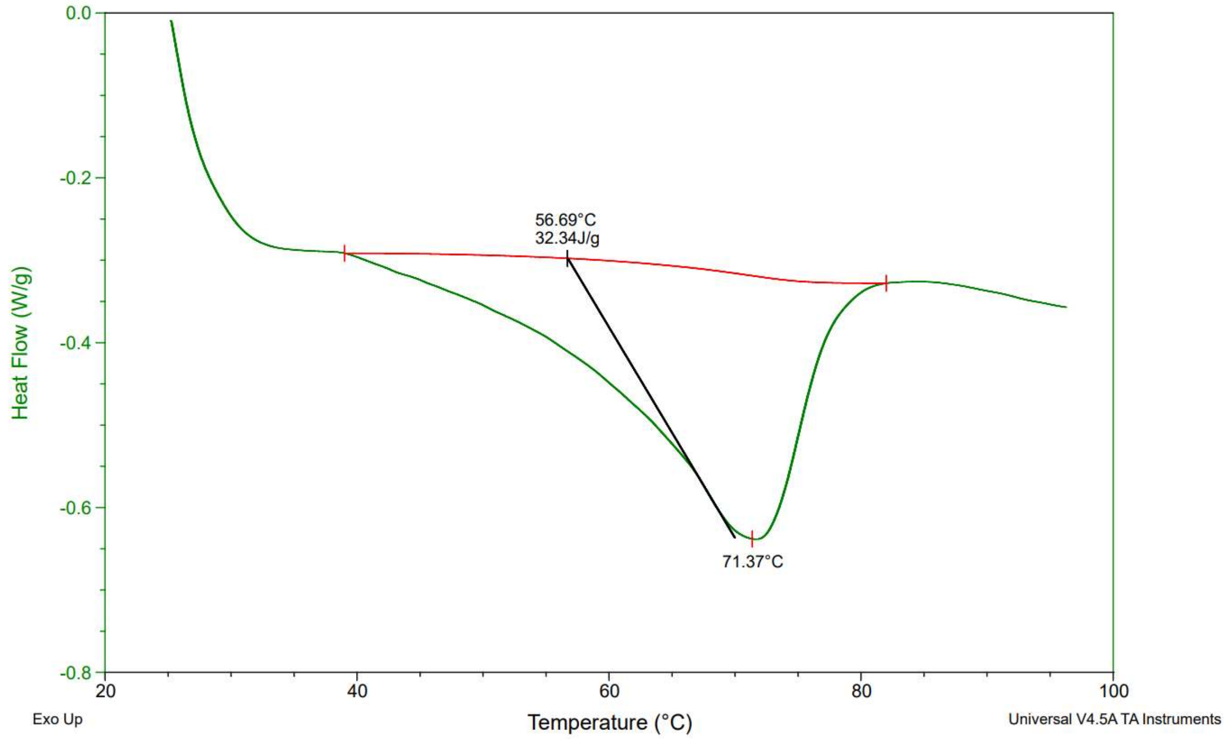

Figure S86. The DSC curves of [L-ProOiPr][IBU].

The TG curves of [AAOiPr][IBU]

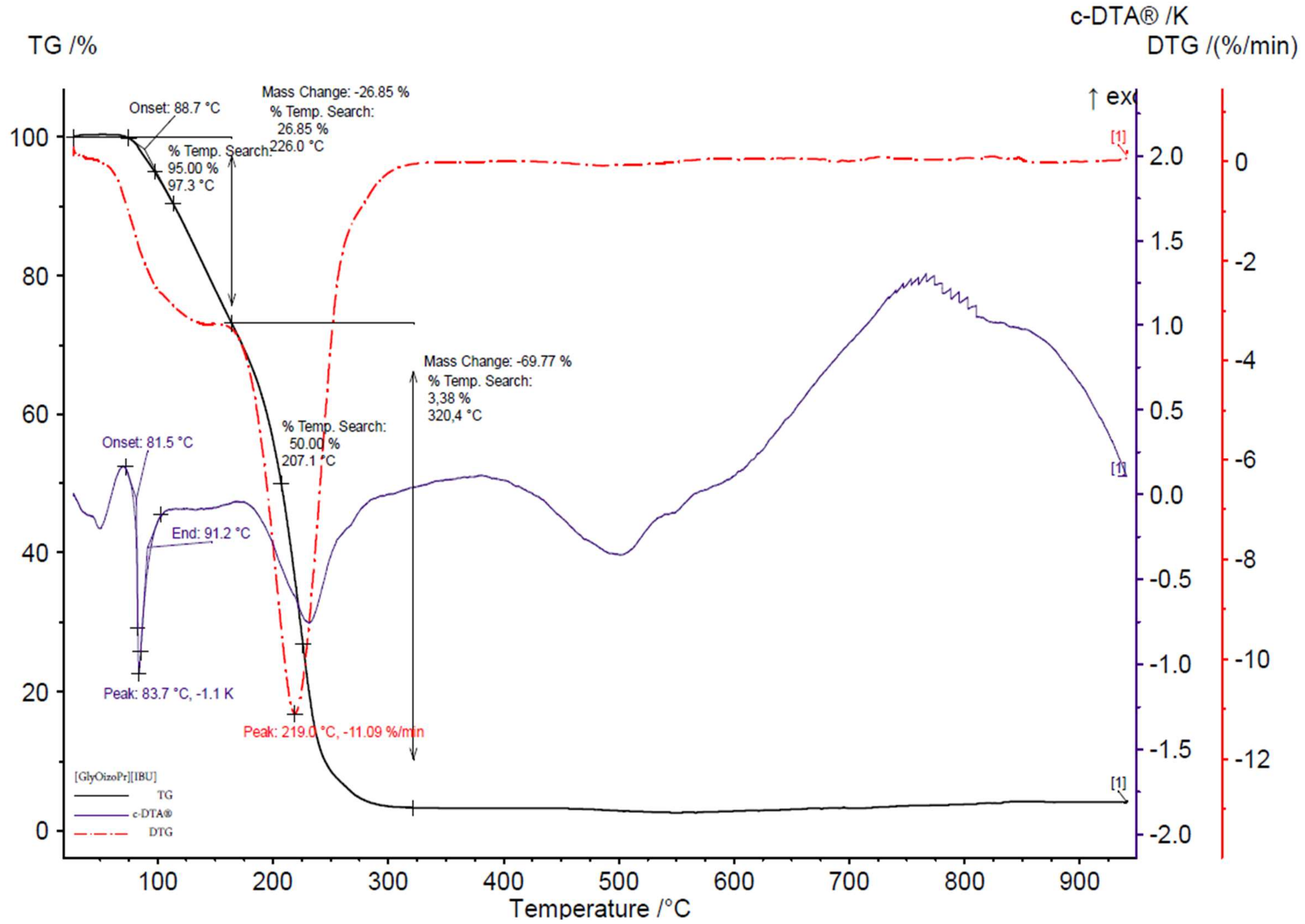

Figure S87. The TG, DTG and c-DTA curves [GlyOiPr][IBU].

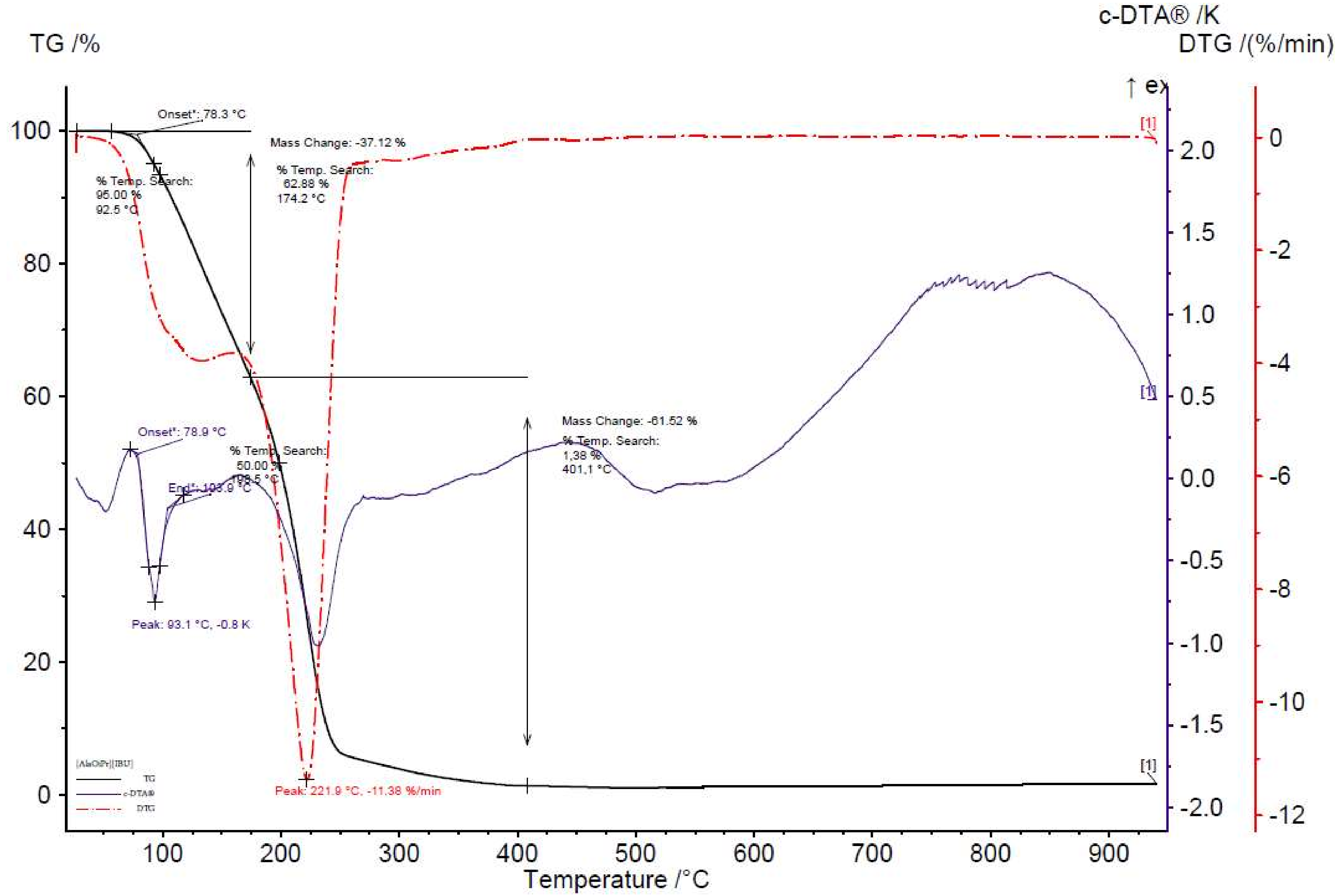

Figure S88. The TG, DTG and c-DTA curves of [L-AlaOiPr][IBU].

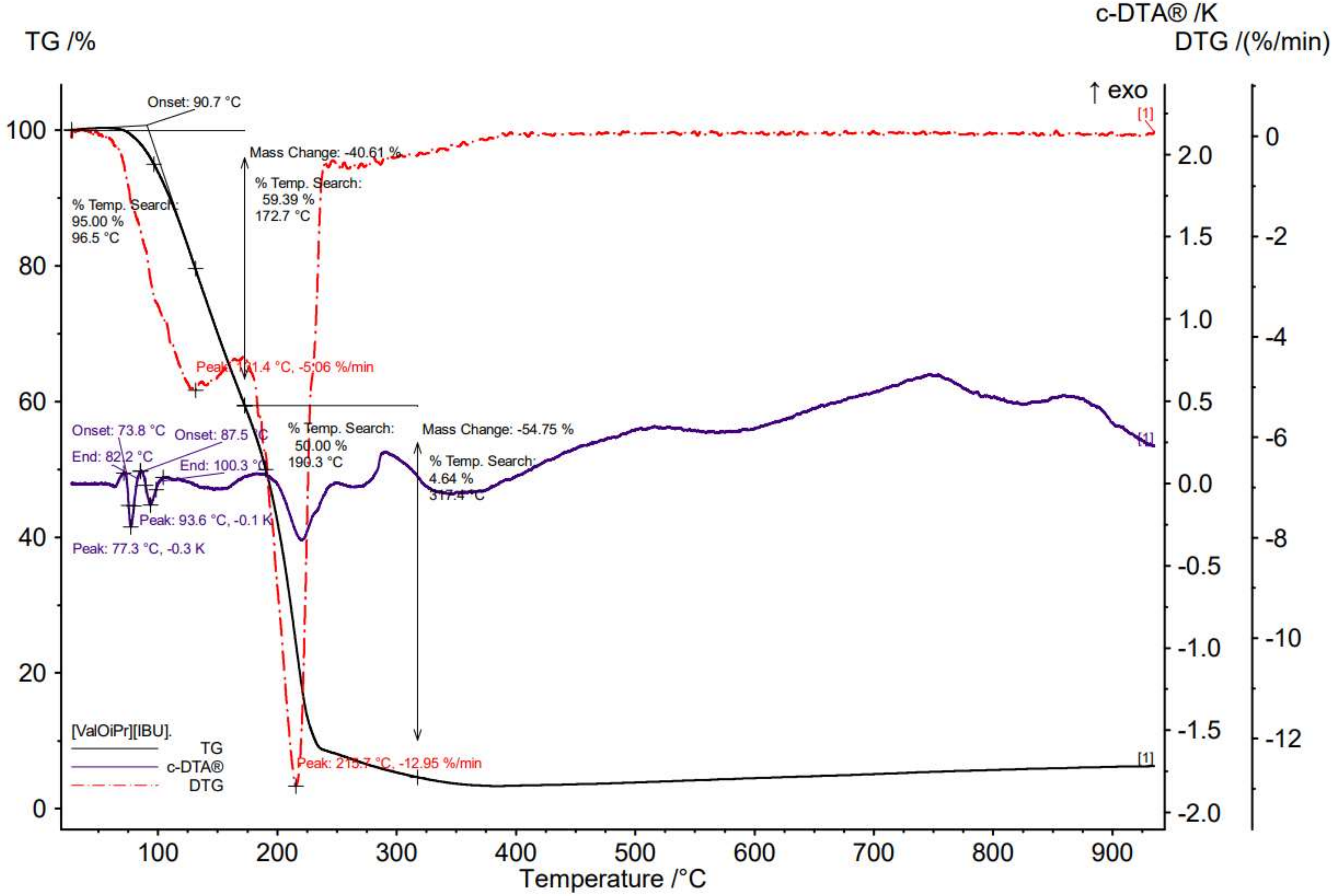

Figure S89. The TG, DTG and c-DTA curves of [L-ValOiPr][IBU].

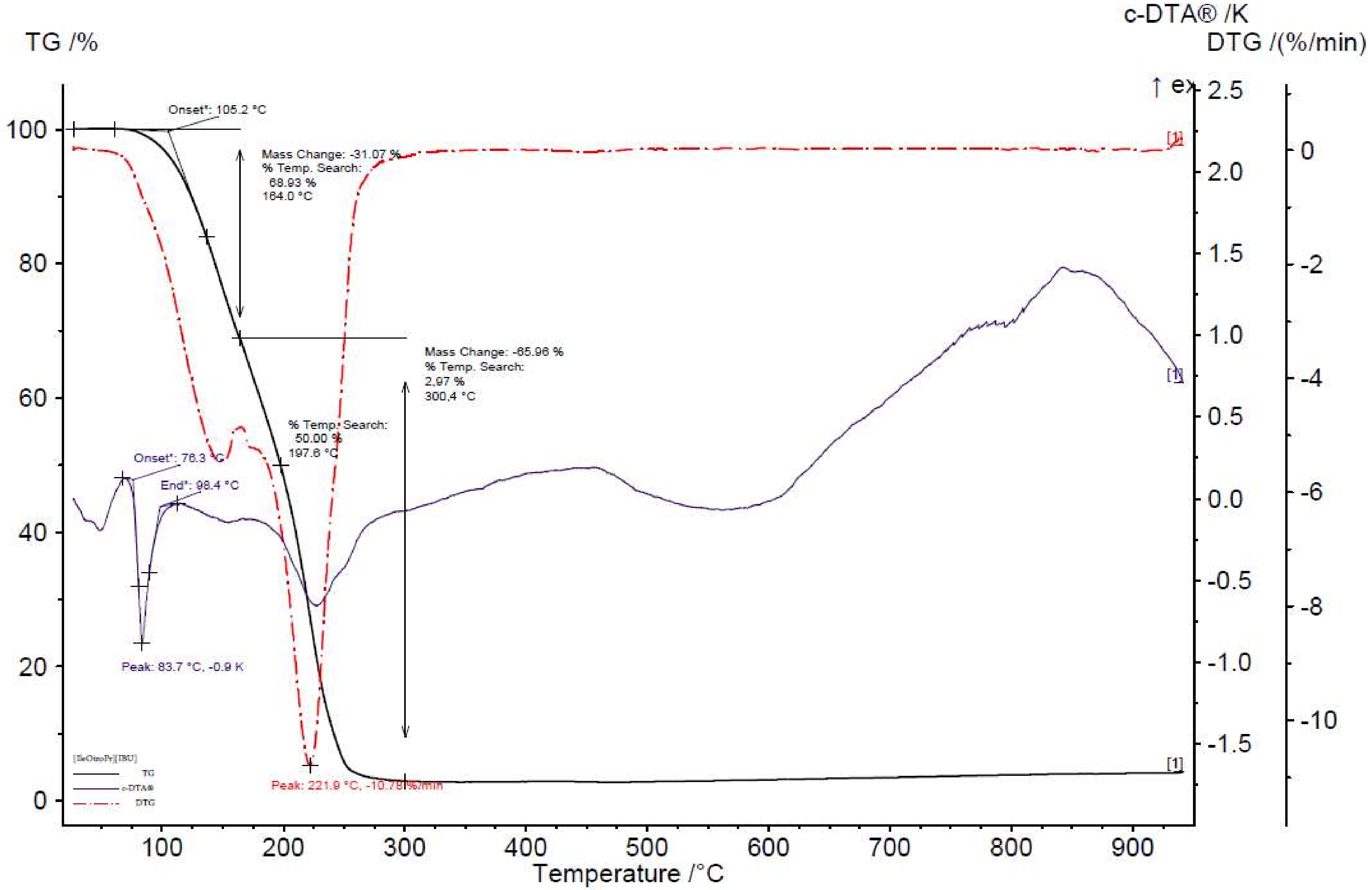

Figure S90. The TG, DTG and c-DTA curves of [L-IleOiPr][IBU].

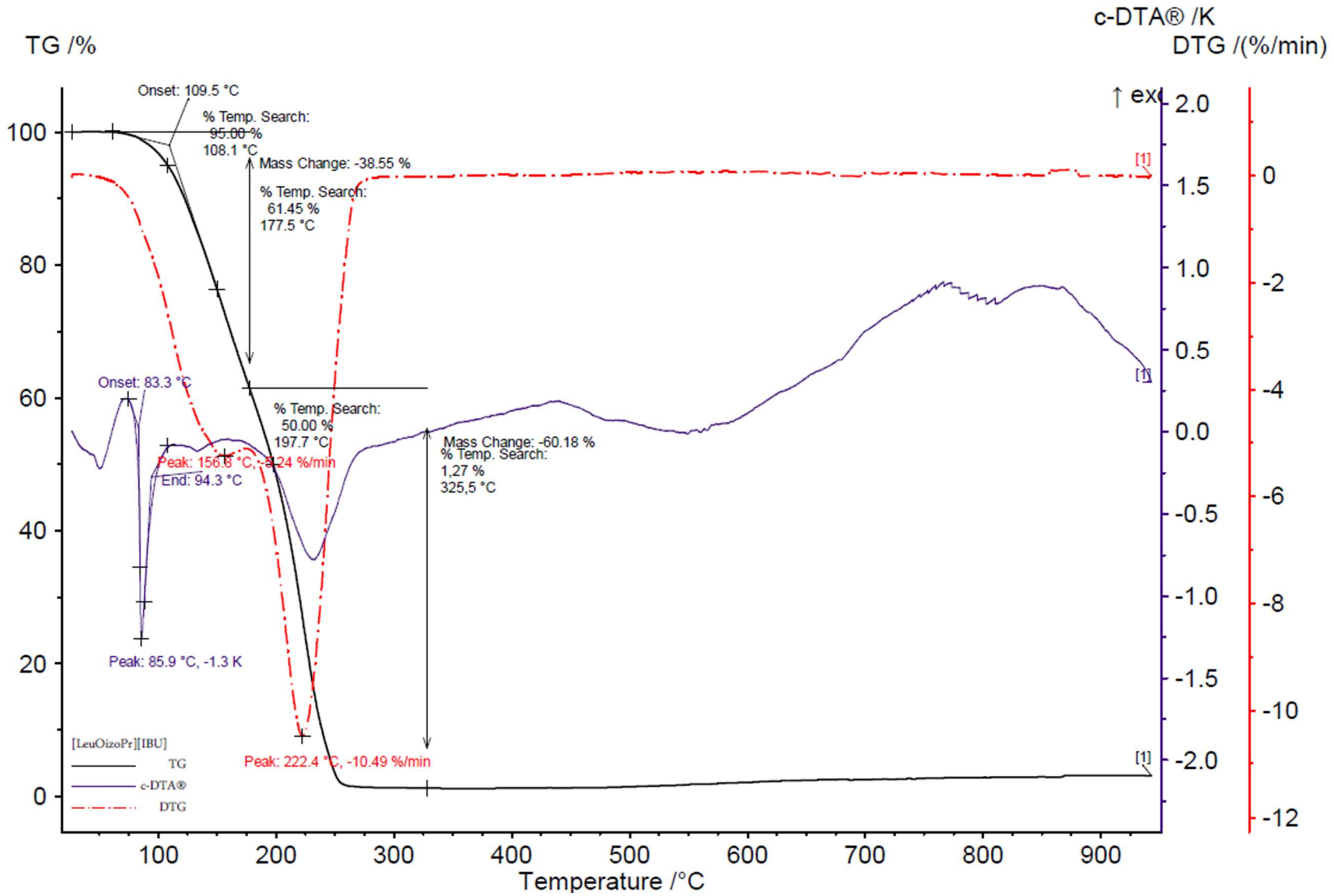

Figure S91. The TG, DTG and c-DTA curves of [L-LeuOiPr][IBU].

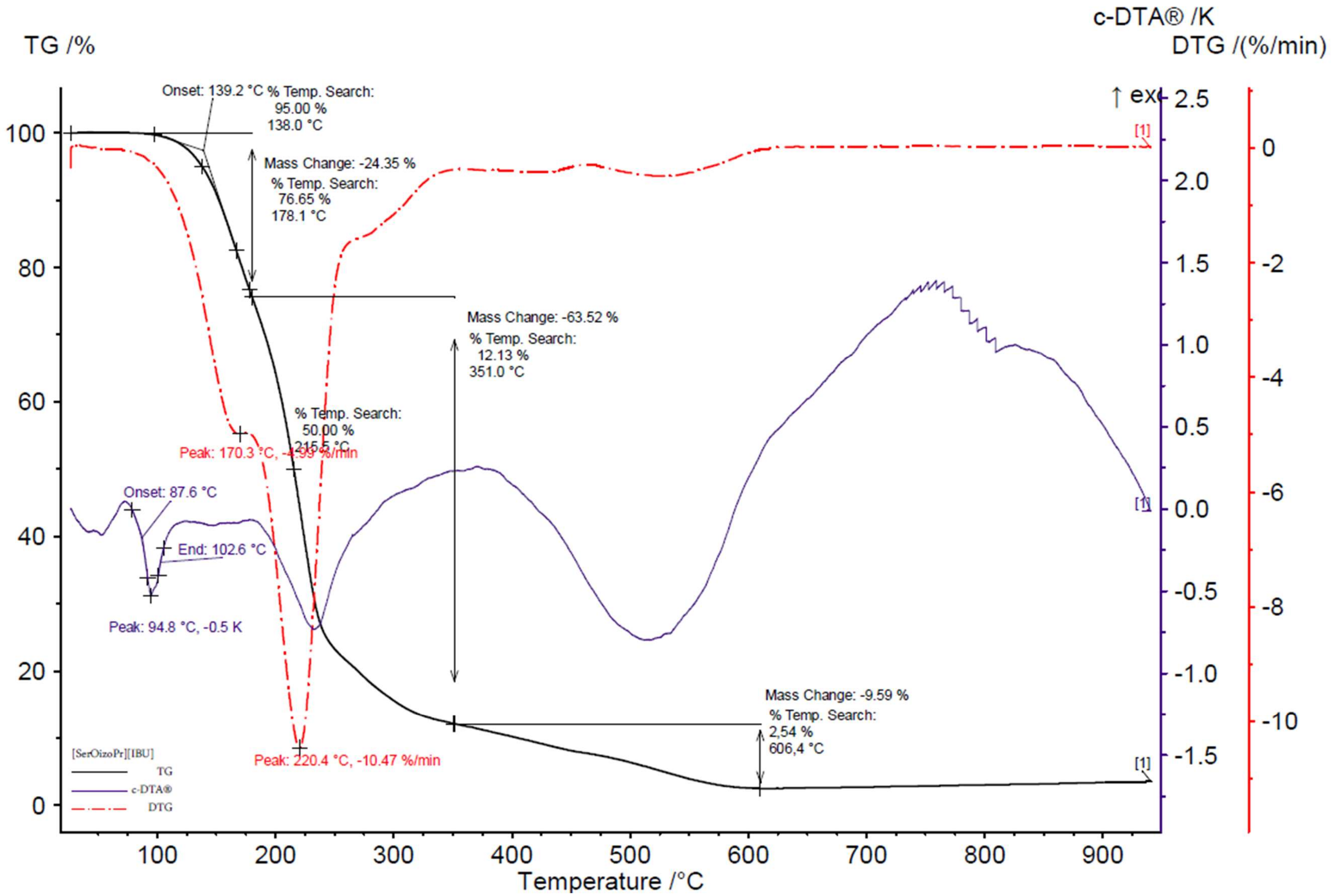

Figure S92. The TG, DTG and c-DTA curves of [L-SerOiPr][IBU].

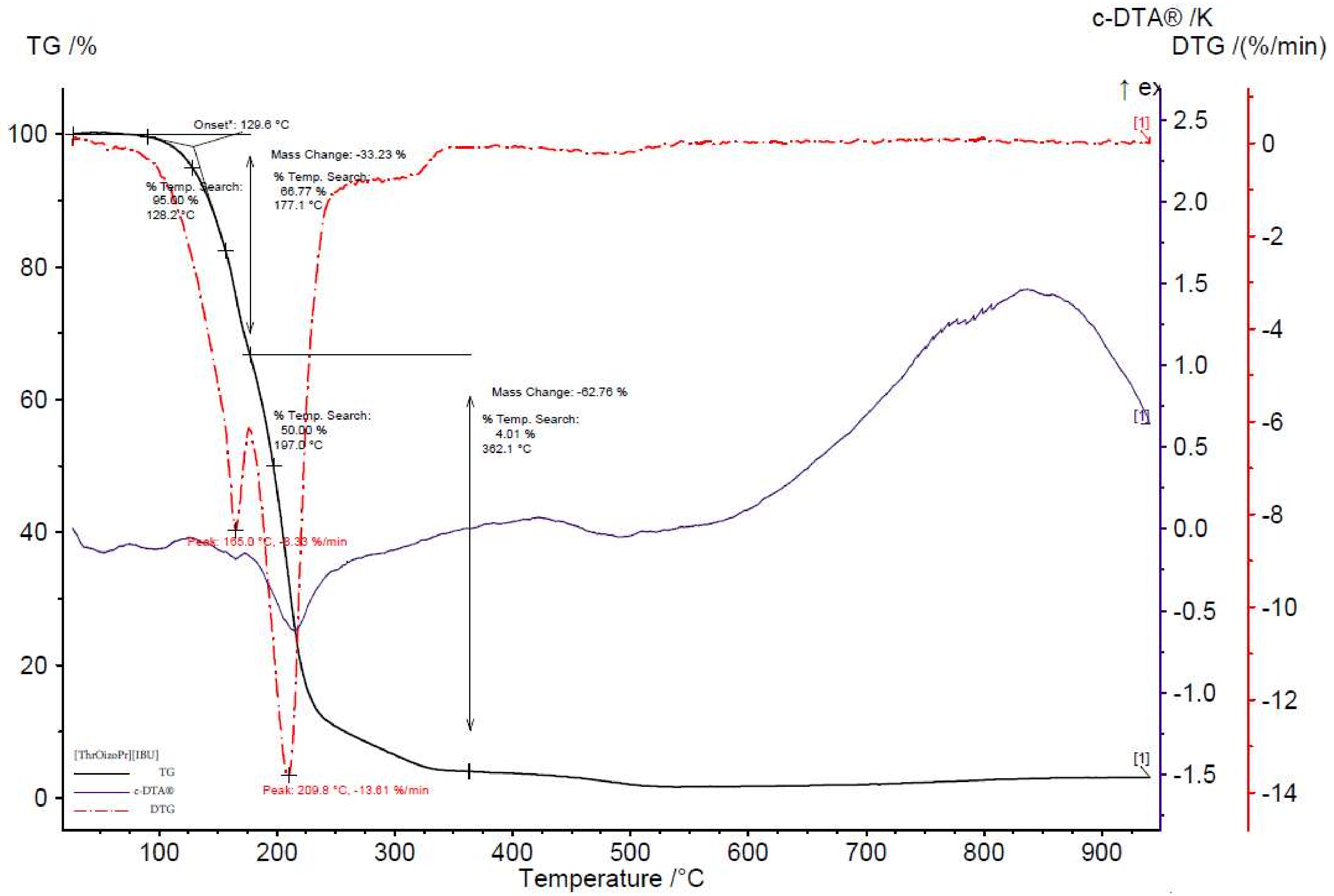

Figure S93. The TG, DTG and c-DTA curves of [L-ThrOiPr][IBU].

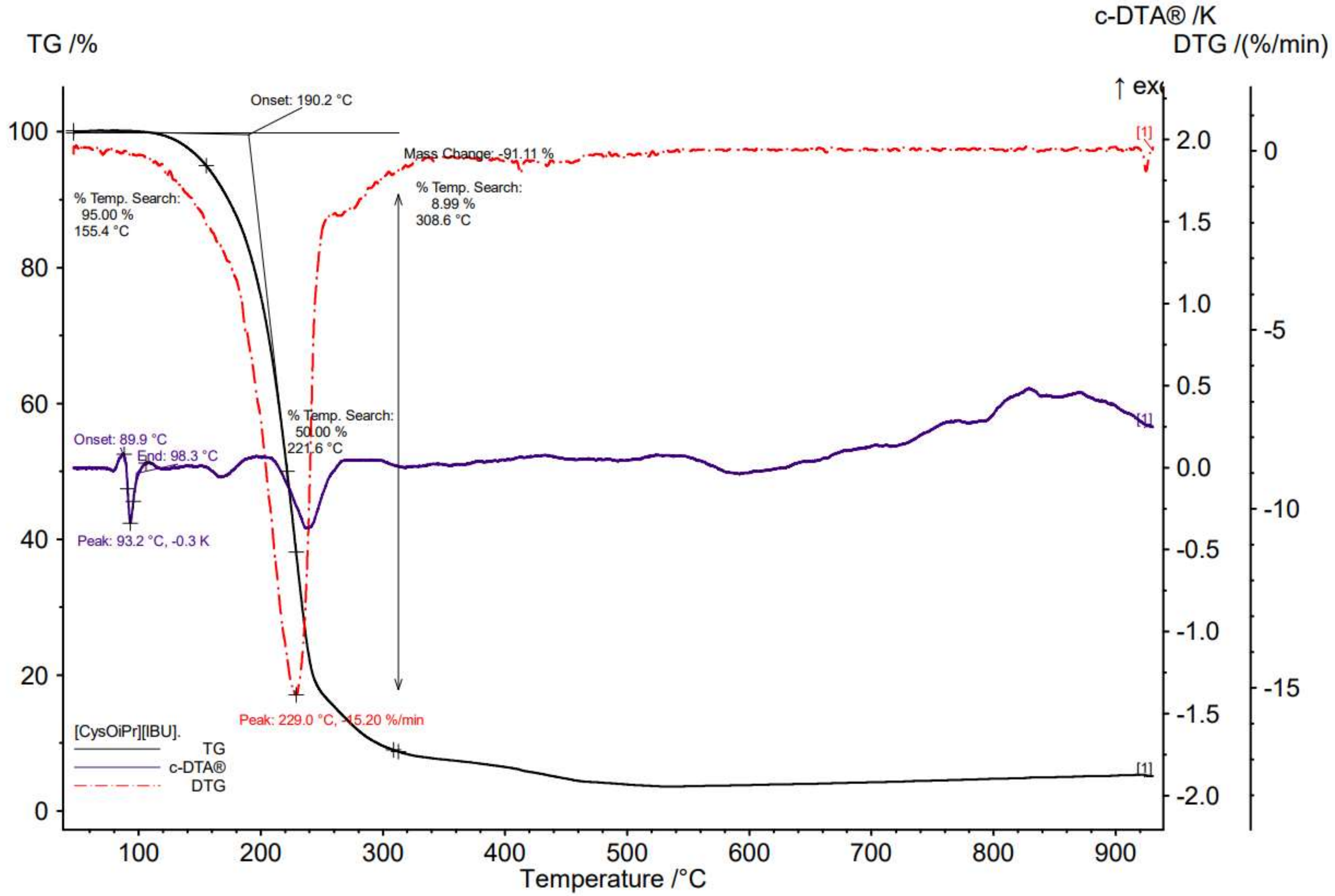

Figure S94. The TG, DTG and c-DTA curves of [L-CysOiPr][IBU].

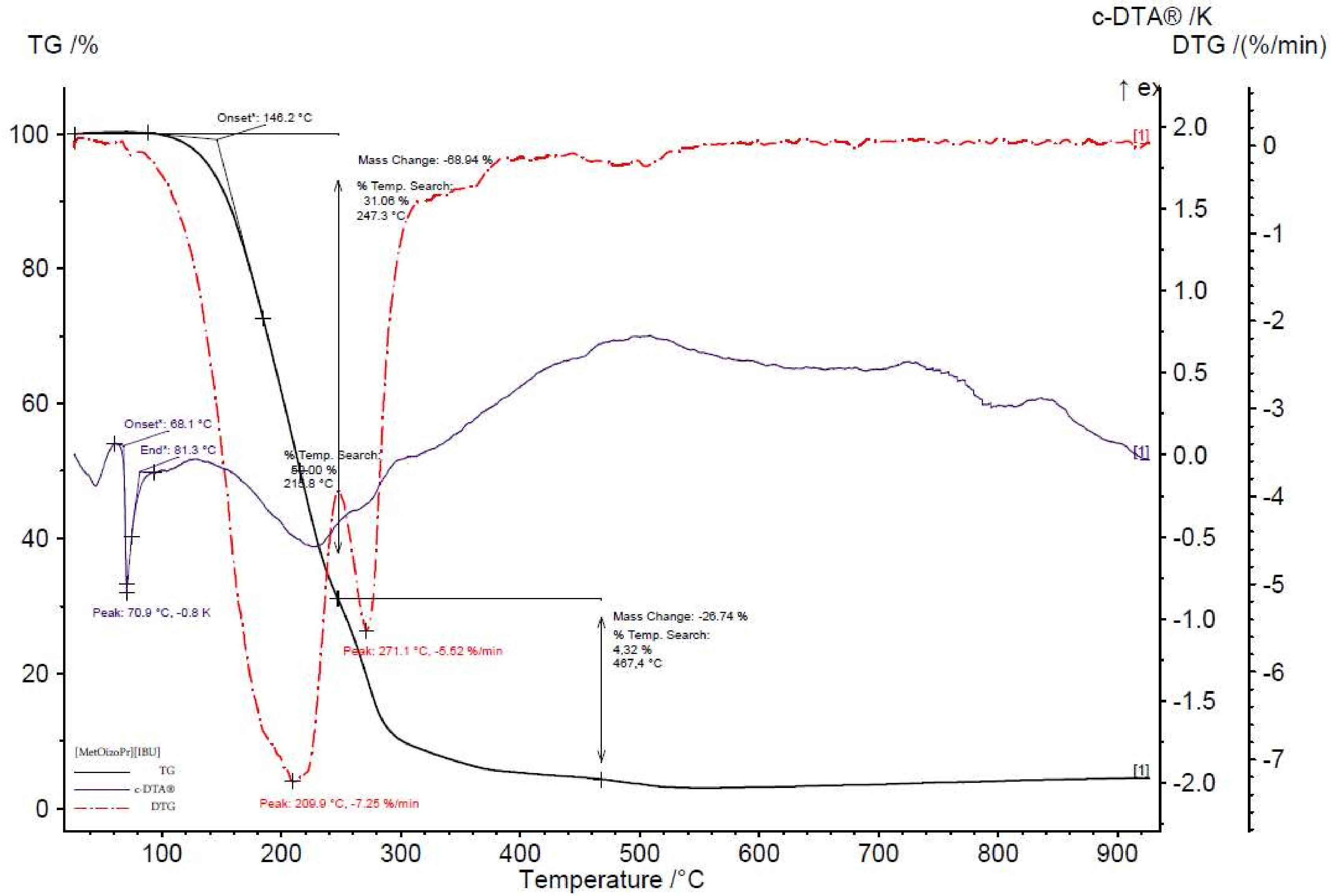

Figure S95. The TG, DTG and c-DTA curves of [L-MetOiPr][IBU].

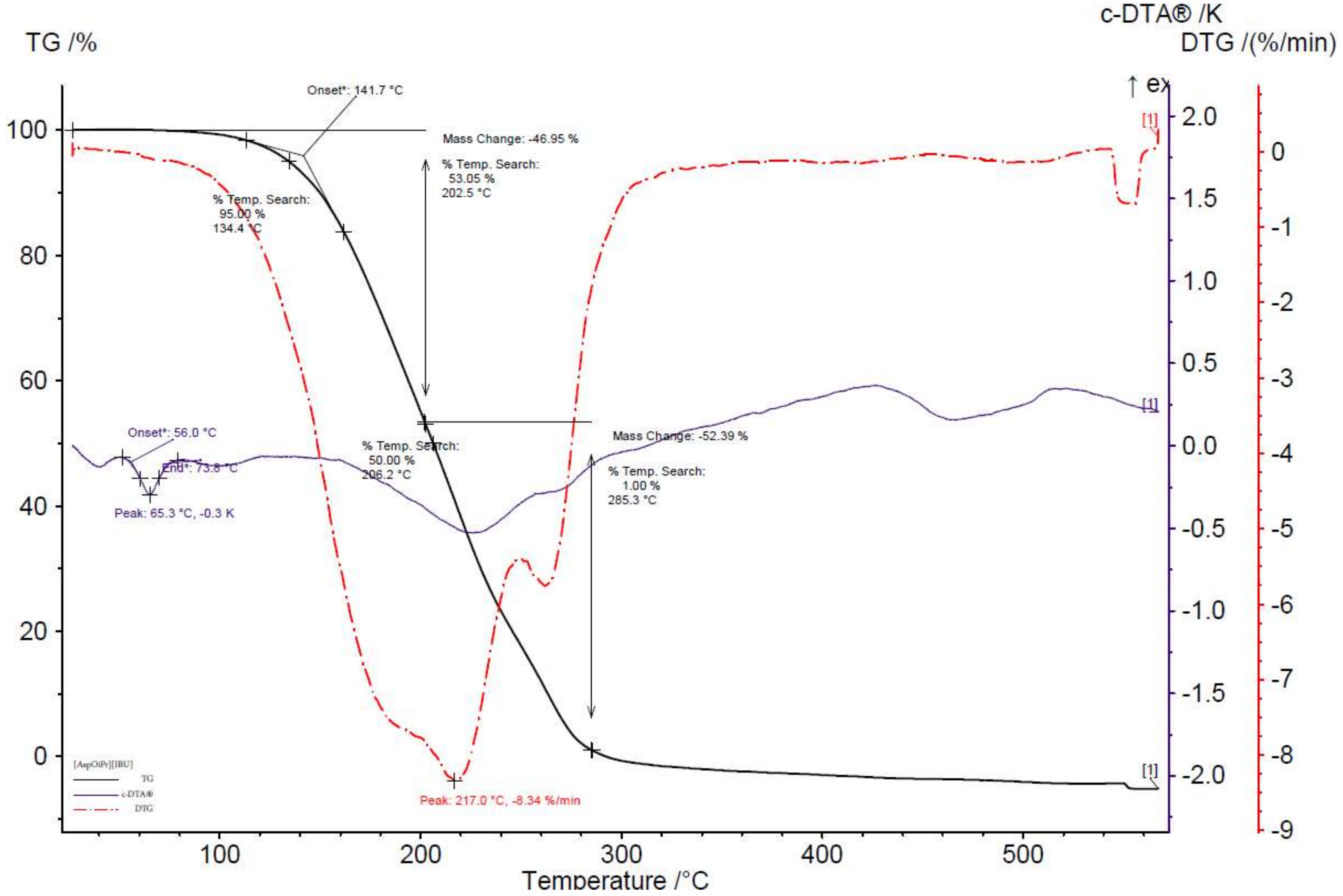

Figure S96. The TG, DTG and c-DTA curves of [L-Asp(OiPr)$_2$][IBU].

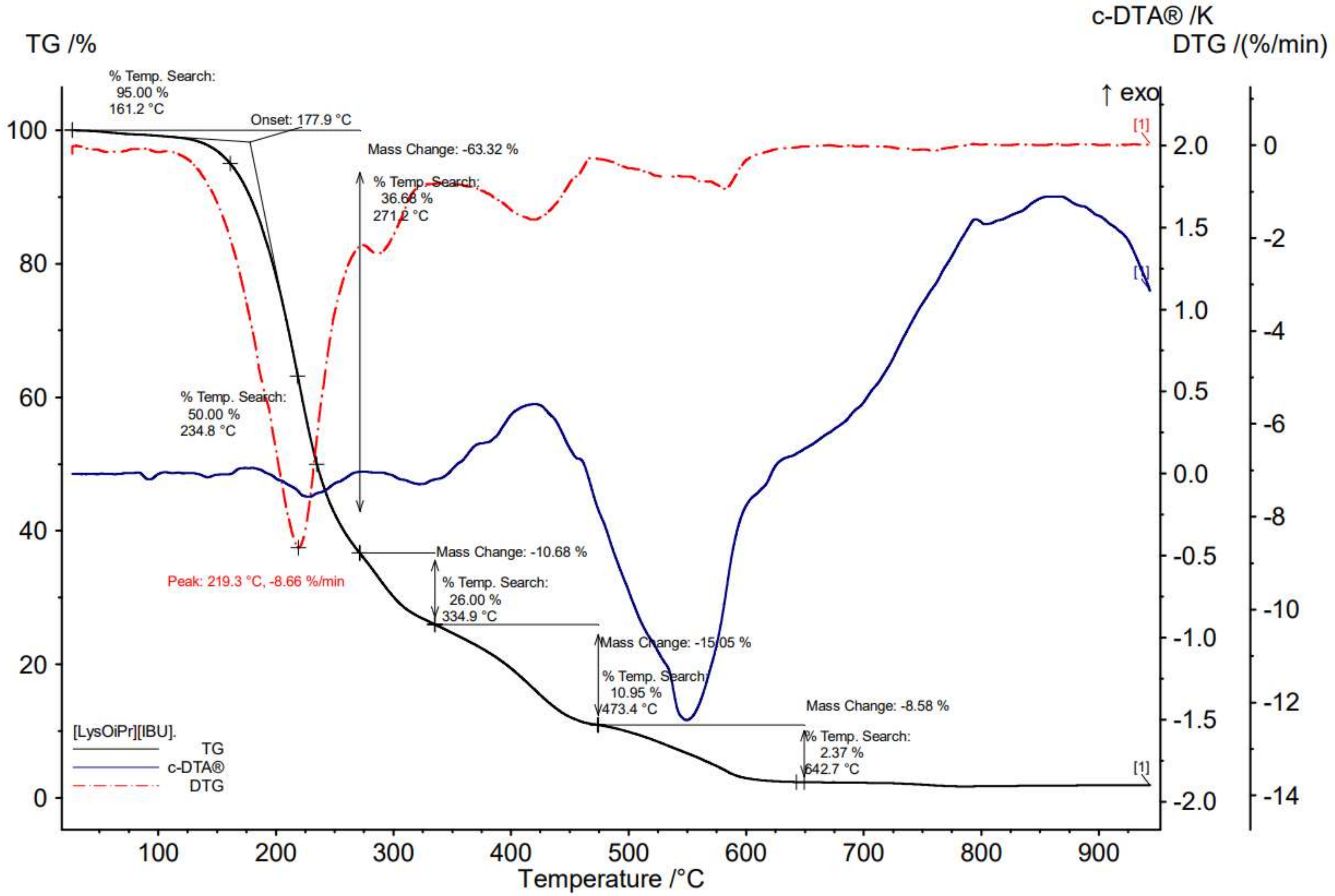

Figure S97. The TG, DTG and c-DTA curves of [L-LysOiPr][IBU].

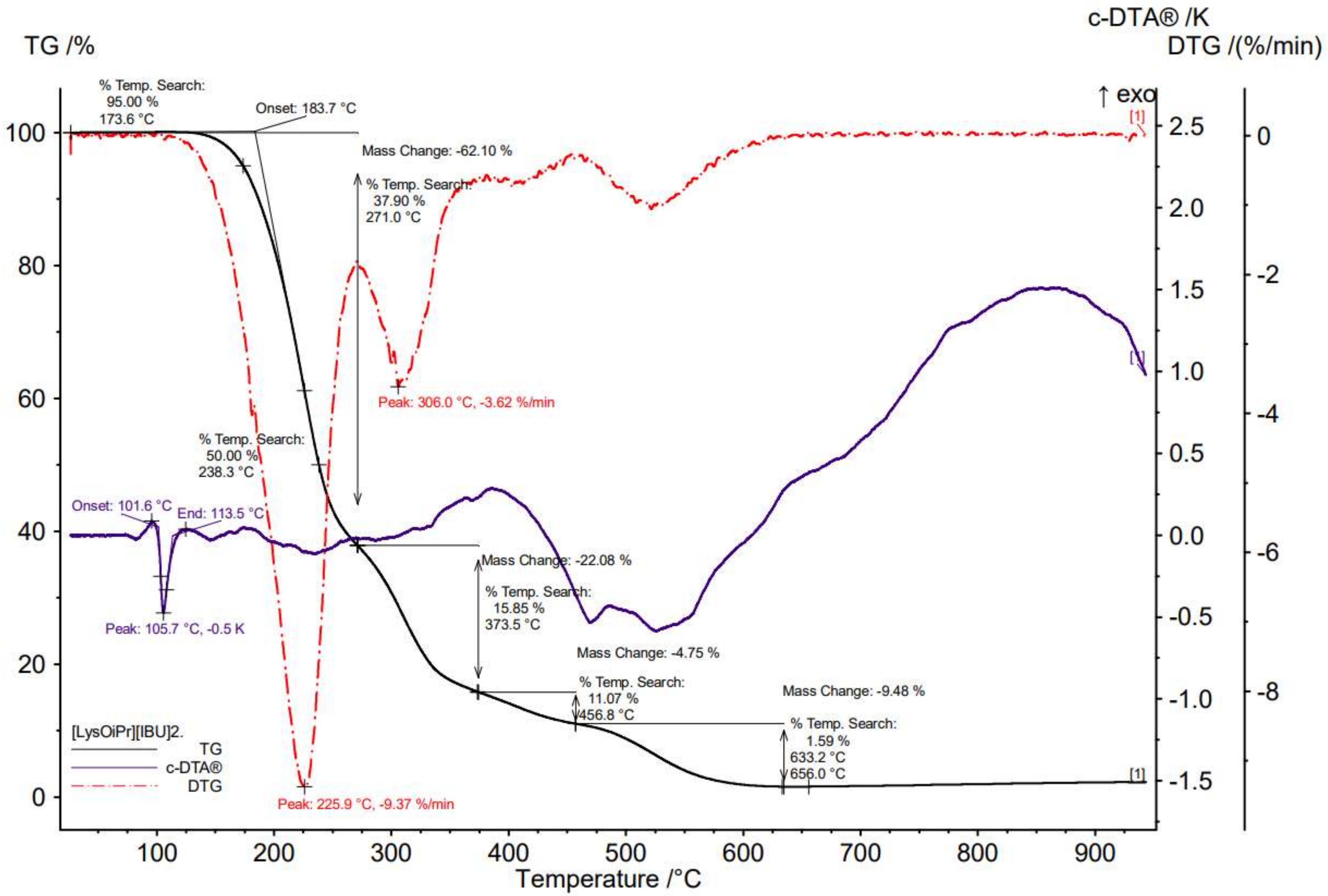

Figure S98. The TG, DTG and c-DTA curves of [L-LysOiPr][IBU]$_2$.

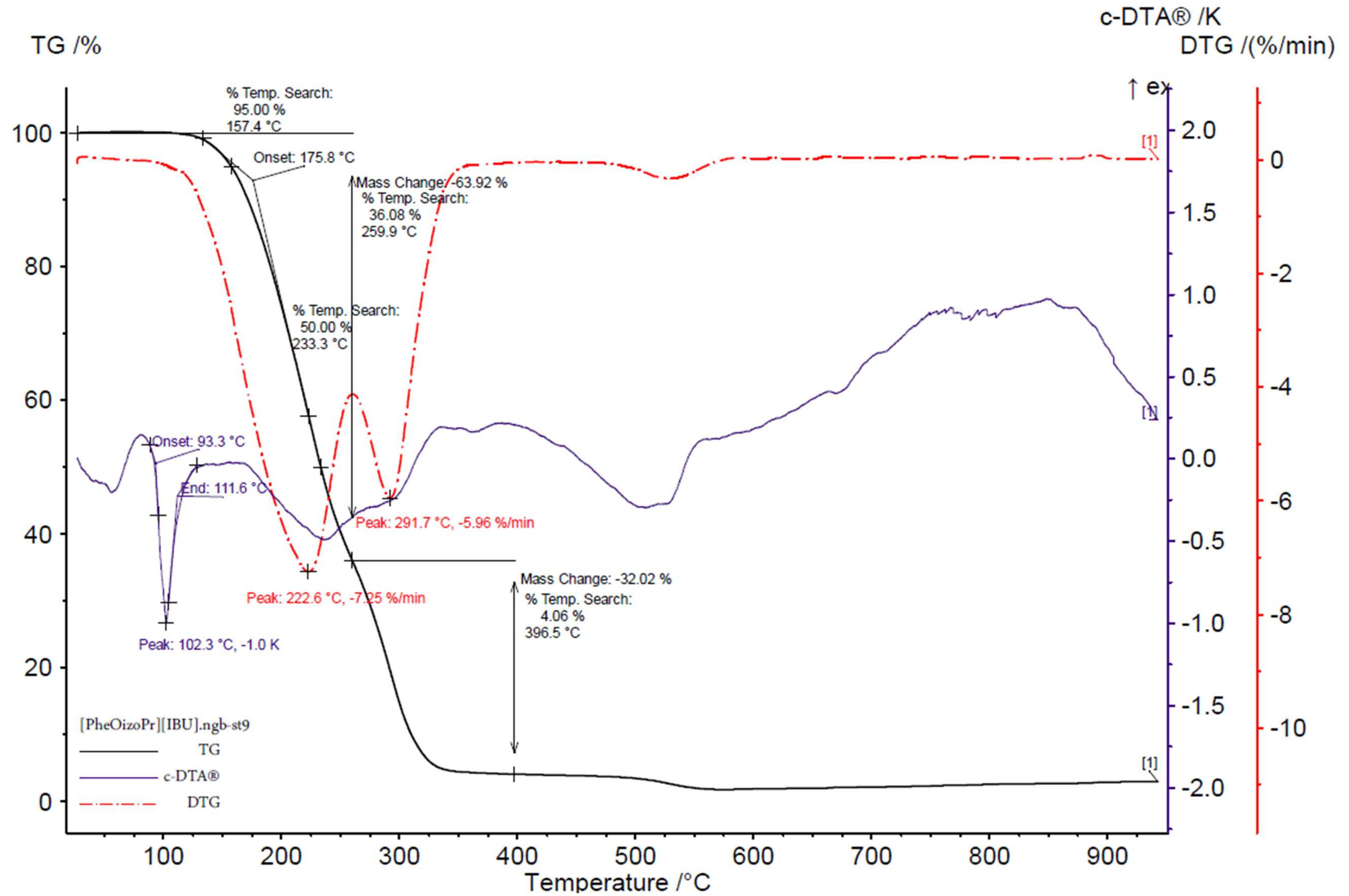

Figure S99. The TG, DTG and c-DTA curves of [L-PheOiPr][IBU].

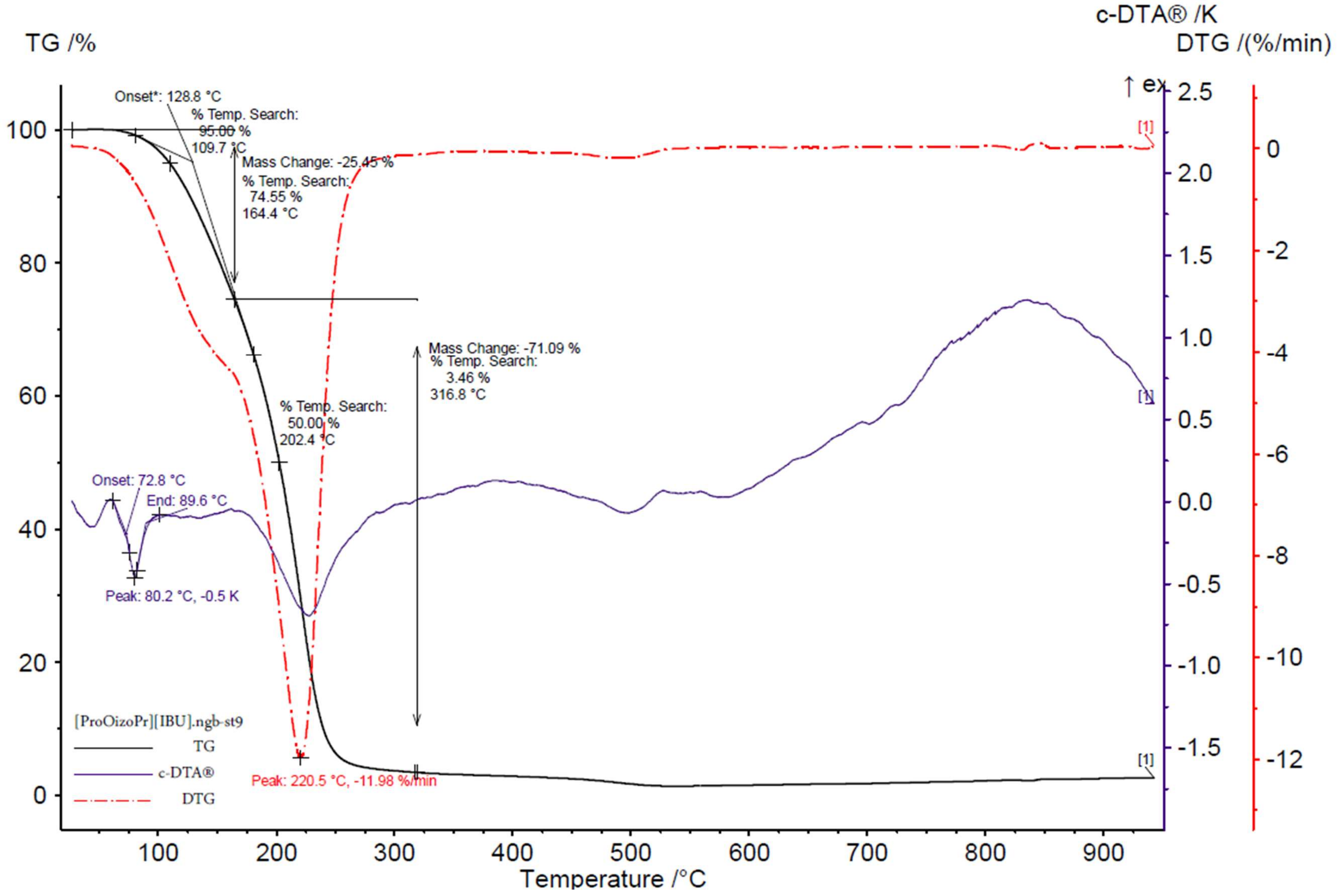

Figure S100. The TG, DTG and c-DTA curves of [L-ProOiPr][IBU].

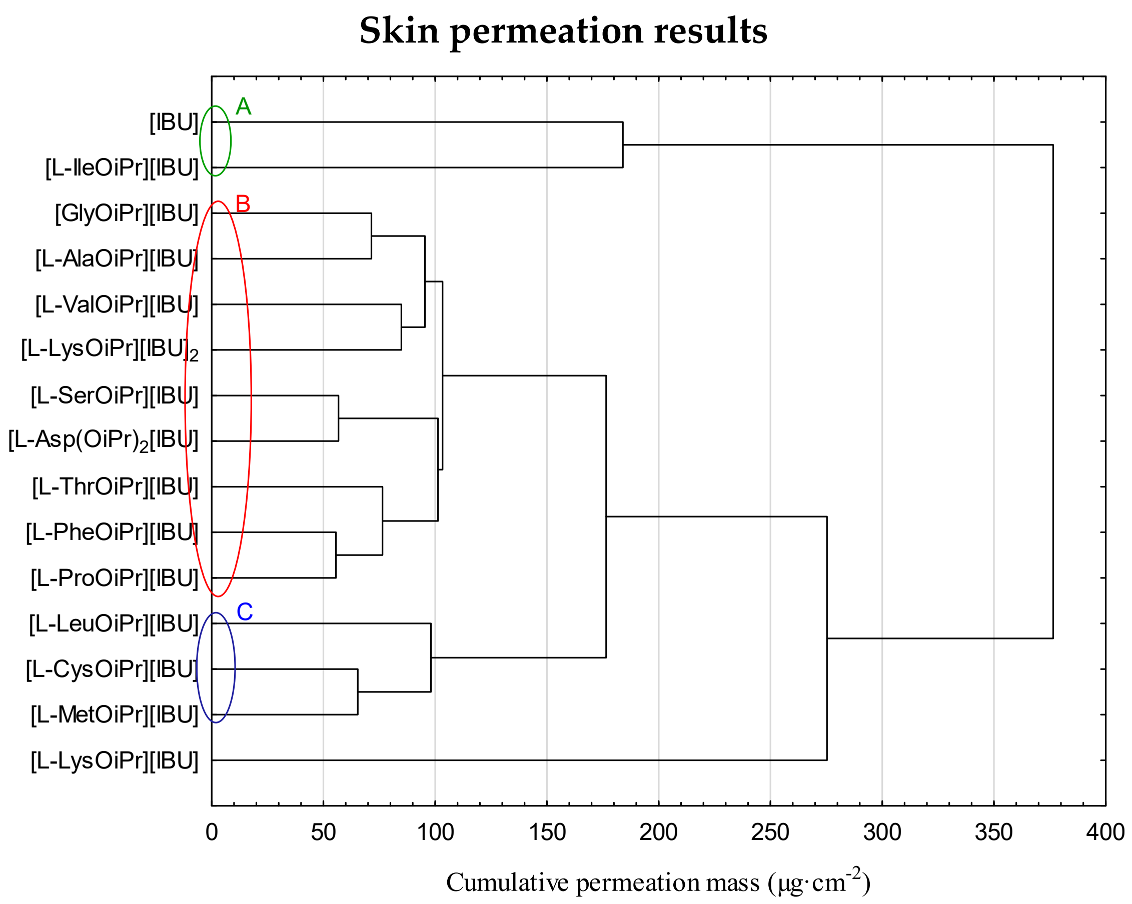

Figure S101. Cluster analysis graph for the mean accumulated mass of ibuprofen and amino acid isopropyl ester ibuprofenates during the entire 24 h study. Compounds with similar values are marked with a green, red or blue circle.

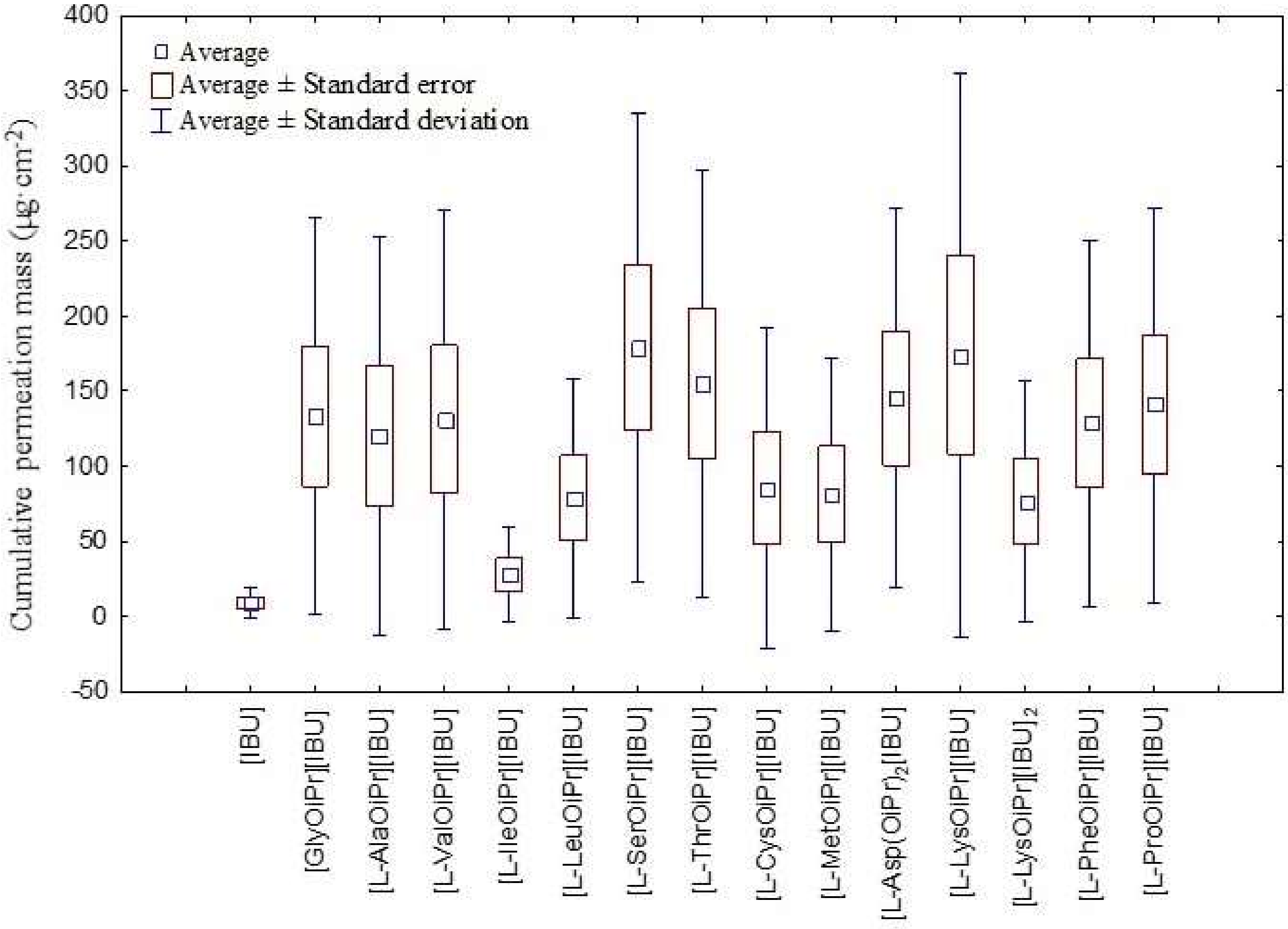

Figure S102. The box-plot of cumulative mass for ibuprofen and amino acid isopropyl ester ibuprofenates after 24 h permeation.

Table S1. Significant differences in the cumulative mass between all analysed compounds, taking into account all time points during the entire 24 h permeation, were estimated by the Mann–Whitney's test; * - significant differences between individual compounds ($p < 0.05$).

	[IBU]	[GlyOiPr] [IBU]	[L-AlaOiPr] [IBU]	[L-ValOiPr] [IBU]	[L-IleOiPr] [IBU]	[L-LeuOiPr] [IBU]	[L-SerOiPr] [IBU]	[L-ThrOiPr] [IBU]	[L-CysOiPr] [IBU]	[L-MetOiPr] [IBU]	[L-Asp(OiPr)$_2$] [IBU]	[L-LysOiPr] [IBU]	[L-LysOiPr] [IBU]$_2$	[L-PheOiPr] [IBU]	[L-ProOiPr] [IBU]
[IBU]		Z = -2.7830 p = 0.0053*	Z = -2.2579 p = 0.0239*	Z = -2.4679 p = 0.0039*	Z = 1.3127 p = 0.1892	Z = -2.1529 p = 0.0313*	Z = -2.8880 p = 0.0038*	Z = 2.07830 p = 0.0053*	Z = 2.257 p = 0.0239*	Z = -2.2579 p = 0.0239*	Z = -2.8880 p = 0.0038*	Z = 2.7830 p = 0.0019*	Z = 2.1529 p = 0.0313*	Z = -2.8880 p = 0.0038*	Z = -2.7830 p = 0.0053*
[GlyOiPr] [IBU]			Z = -0.8926 p = 0.3720	Z = 0.0525 p = 0.9581	Z = 2.0479 p = 0.0405*	Z = -0.3675 p = 0.7131	Z = -1.3652 p = 0.1721	Z = -1.1552 p = 0.2479	Z = 0.6826 p = 0.4948	Z =-0.4725 p = 0.6365	Z = -1.4702 p = 0.1414	Z = -0.4726 p = 0.6365	Z = 0,0525 p = 0,9581	Z = -1.0502 p = 0.2936	Z = -1.0502 p = 0.2936
[L-AlaOiPr] [IBU]				Z = 0.3678 p = 0.7131	Z = -1.5228 p = 0.1278	Z = 0.3675 p = 0.7131	Z = -1,5228 p = 0.1278	Z = -1,4177 p = 0.1562	Z = 0.2625 p = 0.7929	Z = -0.5251 p = 0.9581	Z = -1.6278 p = 0.1035	Z = -0.6826 p = 0.4948	Z = -0,3675 p = 0,7132	Z = -1,3127 p = 0,1892	Z = -1,2077 p = 0.2271
[L-ValOiPr] [IBU]					Z =1,7328 p = 0.0831	Z =1,62782 p = 0.1035	Z = -0.4725 p = 0.6365	Z = -0.4725 p = 0.6365	Z = 0.5776 p = 0.5635	Z = -0.4725 p = 0.6365	Z = -0.6826 p = 0.4948	Z = -0.4725 p = 0.6365	Z = 0.0525 p = 0.9581	Z = -0.2625 p = 0.7928	Z = -0.1575 p = 0.8748
[L-IleOiPr] [IBU]						Z = 1,2077 p = 0.2271	Z = -2.2579 p = 0.0239*	Z = -2.1529 p = 0.0313*	Z = 1.2077 p = 0.2271	Z =1.4177 p = 0.1562	Z = -2.2579 p = 0.0239*	Z = -2,0479 p = 0,0406*	Z = -1,3127 p = 0.1892	Z = -2.1529 p = 0,0313*	Z = -2.0479 p = 0.0405*
[L-LeuOiPr] [IBU]							Z = -1.3127 p = 0.1892	Z = -1.2077 p = 0.2271	Z = 0.0525 p = 0.9581	Z = 0.0525 p = 0.9581	Z = -1.3127 p = 0.1892	Z = -0.9977 p = 0,3184	Z = -0.5776 p = 0.5635	Z = -0.9976 p = 0.3184	Z = -0.8926 p = 0.3720
[L-SerOiPr] [IBU								Z = -0.2625 p = 0.7928	Z = 1.4178 p = 0.1562	Z = -1.3127 p = 0.1892	Z = -0.8926 p = 0.3720	Z = 0.1575 p = 0.8749	Z = 0.5776 p = 0.5635	Z = -0.2625 p = 0.7928	Z = -0.9976 p = 0.3184
[L-ThrOiPr] [IBU]										Z = -0.8926 p = 0.3720	Z= -0.2625 p = 0.7928	Z = -1.3327 p = 0.1892	Z = 0.3675 p = 0.7131	Z = -0.4725 p = 0.6365	Z = -0.9976 p = 0.3184
[L-CysOiPr] [IBU]											Z = -1.3127 p = 0.1892	Z = 0.6365 p = 0.2701	Z = -0.5776 p = 0.5635	Z = 0.8927 p = 0.3720	Z = 0.8927 p = 0.3720
[L-MetOiPr] [IBU]												Z = -0.8926 p = -0.8926	Z = -0.4726 p = 0.6365	Z = -0.9976 p = 0.3184	Z = -0.7876 p = 0.4308
[L-Asp(OiPr)$_2$] [IBU]												Z = -0,4726 p = 0,6365	Z = 0.6826 p = 0.4948	Z = -0.2625 p = 0.7928	Z = -0.9976 p = 0.3184
[L-LysOiPr] [IBU]													Z = 0.4726 p = 0,6365	Z = -0.0525 p = 0.9581	Z = -0.4725 p = 0.6365
[L-LysOiPr] [IBU]$_2$														Z = 0.3675 p = 0.7131	Z = 0.3675 p = 0.7132
[L-PheOiPr] [IBU]															Z = -0.7876 p = 0.4308
